# Wnt signaling and polarity in freshwater sponges

**DOI:** 10.1186/s12862-018-1118-0

**Published:** 2018-02-02

**Authors:** Pamela J. Windsor Reid, Eugueni Matveev, Alexandra McClymont, Dora Posfai, April L. Hill, Sally P. Leys

**Affiliations:** 1 Department of Biological Sciences, University of Alberta, Edmonton, AB Canada; 2 Department of Biological Sciences, MacEwan University, Edmonton, AB Canada; 3 Department of Biology, University of Richmond, Richmond, VA USA; 4 Department of Molecular Genetics and Microbiology, Duke University, Durham, NC USA

**Keywords:** Wnt, β-catenin, Porifera, Body polarity, Metazoan evolution, Osculum

## Abstract

**Background:**

The Wnt signaling pathway is uniquely metazoan and used in many processes during development, including the formation of polarity and body axes. In sponges, one of the earliest diverging animal groups, Wnt pathway genes have diverse expression patterns in different groups including along the anterior-posterior axis of two sponge larvae, and in the osculum and ostia of others. We studied the function of Wnt signaling and body polarity formation through expression, knockdown, and larval manipulation in several freshwater sponge species.

**Results:**

Sponge Wnts fall into sponge-specific and sponge-class specific subfamilies of Wnt proteins. Notably *Wnt* genes were not found in transcriptomes of the glass sponge *Aphrocallistes vastus. Wnt* and its signaling genes were expressed in archaeocytes of the mesohyl throughout developing freshwater sponges. Osculum formation was enhanced by GSK3 knockdown, and Wnt antagonists inhibited both osculum development and regeneration. Using dye tracking we found that the posterior poles of freshwater sponge larvae give rise to tissue that will form the osculum following metamorphosis.

**Conclusions:**

Together the data indicate that while components of canonical Wnt signaling may be used in development and maintenance of osculum tissue, it is likely that Wnt signaling itself occurs between individual cells rather than whole tissues or structures in freshwater sponges.

**Electronic supplementary material:**

The online version of this article (10.1186/s12862-018-1118-0) contains supplementary material, which is available to authorized users.

## Background

One of the most intriguing transitions in evolution is that of the origin of the first multicellular animals. We now know that transition involved a massive expansion of gene families (e.g. [1–3]), and while the ancestry of many developmental pathways can be traced to pre-metazoan eukaryotes (e.g. [4–6]), a select few, such as the Wnt pathway are uniquely metazoan.

Wnts are one of the most widely used ligands in the development of many animal phyla [7], but one of their central roles is that of axial patterning. Canonical Wnt/β-catenin (β-cat) signaling allows β-catenin to enter the nucleus to bind TCF-LEF and activate downstream genes. Evidence for the canonical Wnt/β-cat signaling pathway in cnidarians include polarized expression patterns of Wnt and Wnt pathway components [8], experiments to activate Wnt using glycogen synthase kinase-3 (GSK-3) inhibitors such as lithium chloride, alsterpaullone or BIO [9–12], and localization of β-catenin within the nuclei of developing embryos [10]. Classical transplantation experiments have also shown that the blastoporal region in the cnidarian *Nematostella* can induce a second axis, indicating that in both cnidarians and vertebrates canonical Wnt signaling establishes body axes [13].

The pre-history of the Wnt pathway is intriguing. GSK-3 is known from the choanoflagellate *Monosiga brevicollis* [6], and the slime mold *Dictyostelium* has GSK-3, which interacts with a β-catenin homolog Aardvark (*Aar*), as well as several homologs of the Frizzled (fz) receptor [14]. If the Wnt pathway in its entirety is unknown in pre-metazoans, when did canonical Wnt signaling arise and for which developmental functions?

As the earliest diverging groups, both ctenophores and sponges should shed light on these questions. The ctenophore *Pleurobrachia pileus* has four *Wnt* genes, all of which are expressed around the mouth [15]. Four *Wnt*s were also found in *Mnemiopsis leidyi* but their expression is associated with the aboral pole and tentacle apparatus [16]. Pharmacological experiments to activate the Wnt pathway produced no effect in *M. leidyi* [16]; experiments have not been reported in *P. pileus.* So, despite the presence of core Wnt pathway components in ctenophores, it is as yet unclear whether canonical signaling functions to polarize the ctenophore body plan.

The four classes of sponges (Porifera) unsurprisingly have distinct larval development and adult body plans. The demosponge *Amphimedon queenslandica* has three *Wnts*, one of which (*AquwntA*) is expressed at the posterior pole of the larva [17, 18], but few other genes in the Wnt pathway show polarized expression in the larva. In the homoscleromorph *Oscarella carmela* eight *Wnts* have been found, and in *O. lobularis* only three, one of which shows expression around the ostium or incurrent openings for the water filtration system [19]. A calcareous sponge, *Sycon ciliatum,* has a surprising 21 *Wnt* genes, many of which show polarized expression, but pharmacological experiments showed no effect on the expression patterns or polarity of the sponge [20]. Another demosponge, *Halisarca dujardini*, has a larger complement of *Wnt* genes (ten), and two of them are expressed at the posterior end of the larvae while others are expressed in the adult osculum and along the body axis. Their role in axial polarity however, remains unclear [21].

Two other studies have suggested a role for canonical Wnt signaling in formation of the sponge feeding canals. Treatments with GSK3 inhibitors caused ectopic oscula, the vent of the aquiferous system in the freshwater demosponge *Ephydatia muelleri*, [22] and more ostia – water intake pores – to form in the homoscleromorph sponge *Oscarella lobularis*, where *OlowntII* is expressed [19]. In *E. muelleri* transplantation of the osculum induced canals to grow towards it, suggesting the osculum has organizer properties [22]. Together these data suggest that Wnt has some role in the establishment of overall body polarity in a sponge – either swimming polarity in the larva and/or the polarity of the aquiferous canal (feeding) system.

The evidence for canonical Wnt/β-cat signaling in sponges and ctenophores is therefore still equivocal. The apparent absence of components of the Wnt pathway in some sponges (e.g. axin in *Aphrocallistes vastus* and *Sycon coactum* and Wnt and dsh in *A. vastus*; [3]), the inability of pharmacological experiments to affect polarized development in *Sycon* [20], and the failure to pull down axin bound to β-catenin in *Ephydatia muelleri* [23] leave doubt, as do the different expression patterns and inability to activate the pathway using pharmacology in the ctenophore *M. leidyi*. It is possible that canonical Wnt signaling is used in defining axial polarity only in eumetazoans (Cnidaria + Bilateria), and expression at poles of larvae and adult sponges is coincidental. In this scenario components of the Wnt pathway would have been used for other functions in unicellular eukaryotes and pre-eumetazoan animals. For example the genome of *Dictyostelium* contains several elements of Wnt/β-catenin signaling (*Fz*, *GSK3β*, a *β-catenin* homolog and a putative *dkk*; [11, 14]); it is hypothesized that the origin of Wnt and dsh in early metazoans was key in connecting the pieces of this pathway together [7]. Canonical Wnt signaling would have arisen for some function of coordinating grander cell movements, but not necessarily involved in determining the location of polarized structures in pre-eumetazoan animals. Here we examine this hypothesis using a diversity of approaches – in situ expression patterns, RNAi knockdowns, larval manipulations, and pharmacological inhibition experiments – in a diversity of freshwater sponge model systems.

## Results

### Phylogenetic relationships of sponge Wnts

We compiled *wnt* genes from 17 species of sponge from all 4 Poriferan classes, either by searches of our own transcriptomes or of those available online (see Additional file 1), and on NCBI (http://www.ncbi.nlm.nih.gov). *Wnt* genes are in all sponge classes with the notable exception of the glass sponge *Aphrocallistes vastus*. We identified up to 3 Wnts in demosponges (*Ephydatia muelleri*, *Spongilla lacustris*, *Eunapius fragilis*, *Ircinia fasciculata*, *Chondrilla nucula*, *Petrosia ficiformis, Pseudospongosorites suberitoides*), 5 Wnts in the homoscleromorph *Corticium candelabrum* and 13 in the calcareous sponge *Sycon coactum*. Sponge Wnts had the typical 23–24 conserved cysteine residue positions along the protein and a semi-conserved RWNC motif towards the N terminal typical to the Wnt protein sequence (Additional file 2).

**Fig. 1:**
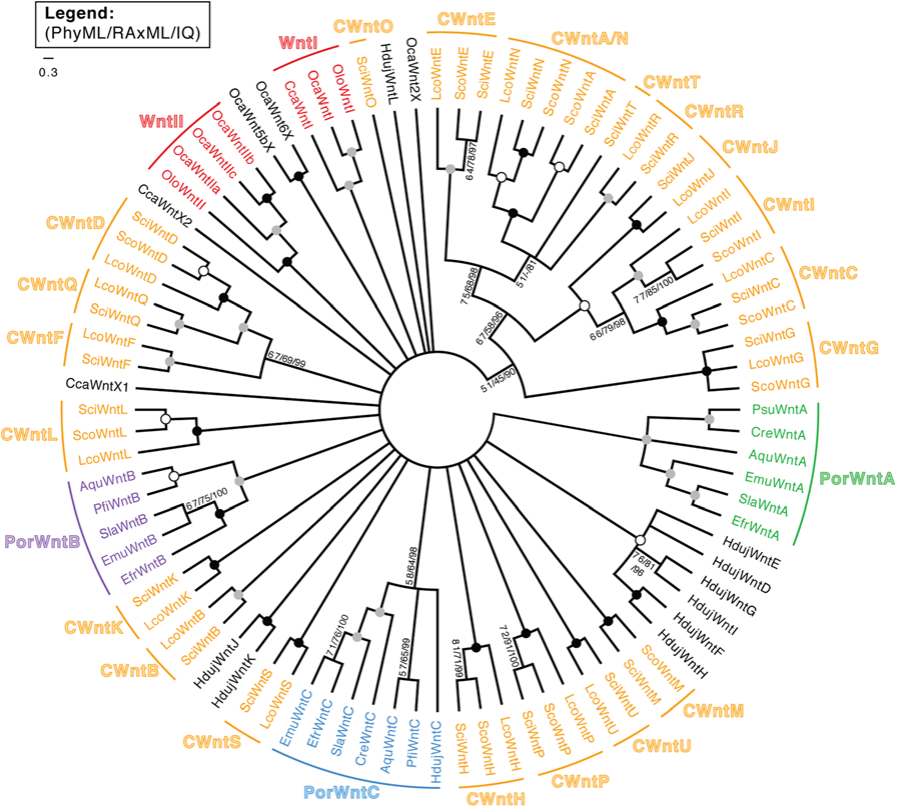
Poriferan Wnt relationships. Consensus tree of Wnt relationships from sponges based on PhyML, RAxML and IQ-TREE analyses. Support values indicated are from PhyML/RAxML/IQ-TREE bootstrap support. A closed circle (●) bootstrap support of 100 for all analyses, a grey circle () indicates support over 90 for all analyses, and an open circle (○) is support of over 80 for all analyses. Taxon abbreviations: Aqu, *Amphimedon queenslandica*; Ava, *Aphrocallistes vastus*; Cca, *Corticium candelabrum*; Cre, *Crella elegans*; Emu, *Ephydatia muelleri*; Efr, *Eunapius fragilis*; Hduj, *Halisarca dujardini*; Lco, *Leucosolenia complicata*, Oca, *Oscarella carmela*; Olo, *Oscarella lobularis*; Pfi, *Petrosia ficiformis*; Psu, *Pseudospongosorites suberitoides*; Sla, *Spongilla lacustris*; Sci, *Sycon ciliatum*; Sco, *Sycon coactum*

To determine the subfamily relationships within sponge Wnts we conducted maximum likelihood analyses of full-length aligned sponge Wnt sequences as shown in the unrooted consensus tree in Fig. 1 (all raw trees are included in Additional file 3). We consistently recovered 3 subfamilies of demosponge Wnts with moderate to high support: PorWntA (95/93/100), PorWntB (98/100/100) and PorWntC (58/67/94). We also recovered the previously reported WntI (92/95/100) and WntII (100/100/100) subfamilies from homoscleromorph sponges, although some Wnt sequences from homoscleromorphs did not appear to group with other Wnts (CcaWntX1, CcaWntX2, OcaWnt2X, OcaWnt5X and OcaWnt6X). The transcriptome of *O. carmela* also has alternate transcripts of WntII, here named OcaWntIIa, b and c. We were able to assign some Wnts from *Sycon coactum* and *Leucosolenia complicata* to the families described in [20], which are here denoted as CWntA through U. These subfamilies were highly supported (98–100 in all analyses), except CWntA and N, which appear to form a highly-supported clade of paralogues (100/100/100) with less support for the internal nodes (CWntA-86/88/100; CWntN-84/73/100). Support in the backbone and deeper nodes of the tree was consistently low, and thus much of the tree was collapsed into a polytomy (Fig. 1). The assignment of sponge Wnts to subfamilies of previously unpublished or unanalyzed sequences is given in Additional file 4.

Sponge Wnts do not fall into the Wnt subfamilies of other metazoans (Additional file 5). Vertebrate Wnt sequences fall with high support into distinct, well-defined families, however backbone support throughout the tree was weak and thus no conclusions can be made about the branching order of Wnt subfamilies and the relationships between them.

### Wnt pathway gene expression during development from the gemmule

We examined the expression patterns of Wnt/β-catenin pathway genes (3 *wnt*, 3 *fz*, 1 *dsh*, 1 *GSK3*, 1 *β-cat*, and 1 *tcf/lef*) in 2 and 5 days post hatch (dph) juveniles of *E. muelleri* using both colourimetric (NBT/BCIP) and fluorophore (FISH) based in situ hybridization detection methods.

**Fig. 2:**
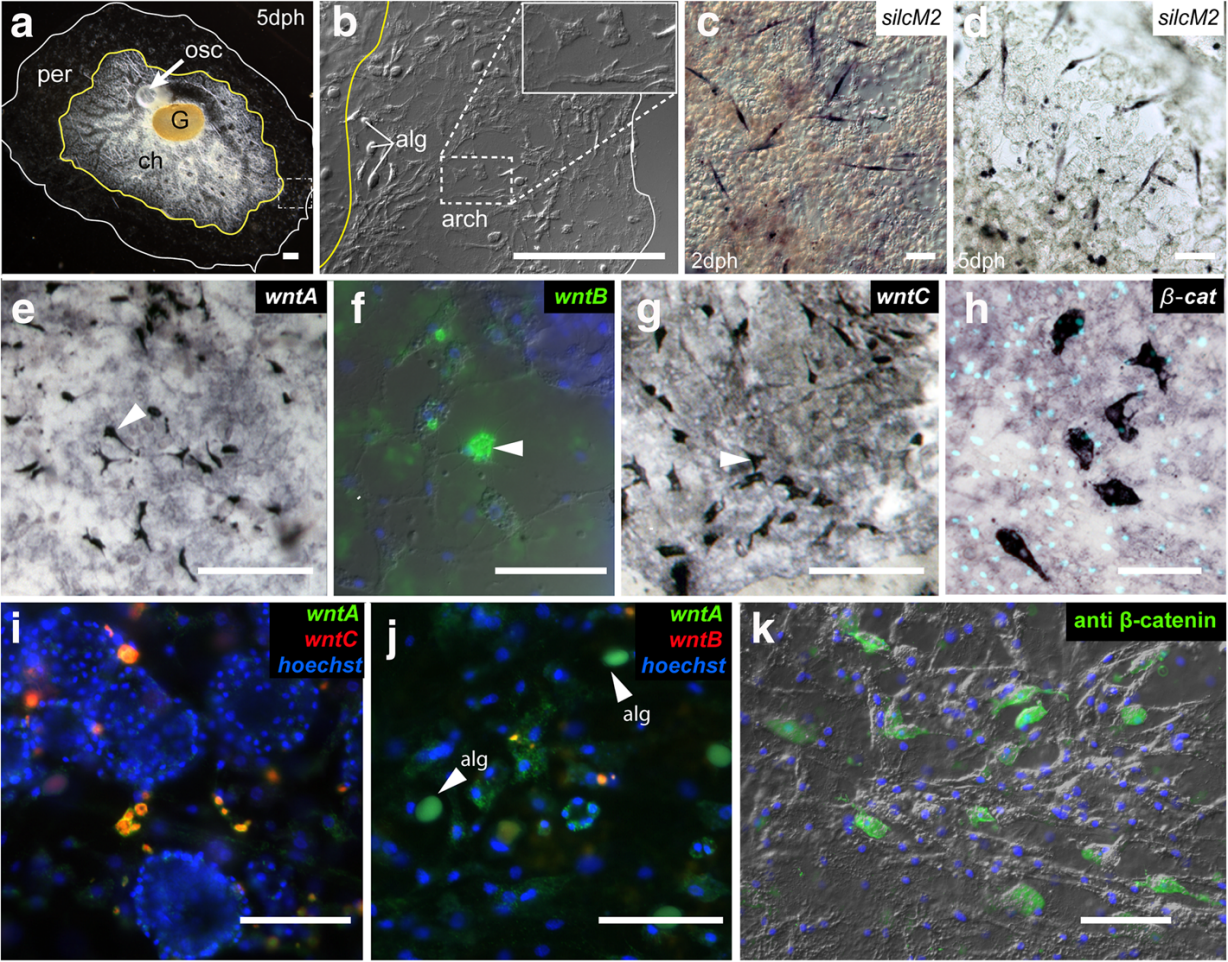
Expression of *wnts* and *β-catenin* reveal cellular and not regional expression patterns. **a**) Whole sponge at 5 dph showing regions of the body including the gemmule, G, osculum, osc, choanosome, ch with choanocyte chambers and canals and bordered in yellow – and the peripheral region, per, shown by the white border. **b**) Expanded view of boxed area in **a**). Archaeocytes (arch) and algal cells (alg) in the peripheral region shown by DIC. Inset shows amoeboid archaeocytes. **c**), **d**) *silicatein M2* (positive control) in situ hybridization in 2 and 5 dph sponges, respectively (whole mount top; inset bottom). Sclerocytes are spindle-like in shape (arrowheads). **e**) - **h**) Expression of *EmuwntA*, *EmuwntB*, *EmuwntC*, and *Emuβ-catenin* mRNA in archaeocytes of the peripheral region. Double in situ hybridizations of **i**) *wntA* and *C* in the choanosome near the choanocyte chambers and **j**) *wntA* and *B* in the peripheral region*.* **c**) - **e**) and **g**) - **h**) NBT/BCIP, **f**) Fluorescent in situ hybridizations with *wnt* labels in green and/or red and nuclei in blue (Hoechst). **k**) Anti-β-catenin staining (green) in peripheral tissue of *Spongilla lacustris*, nuclei are indicated in blue (Hoechst), and merged with DIC. Arrowheads in **e**), **f**) and **g**) indicated labeled archaeocytes, arrowheads in **j**) indicate algal symbiont auto-fluorescence (alg). Scales: 50 μm

Expression of all genes was detected in single cells in the mesohyl, either in the choanosome or in a region peripheral to the choanosome (Fig. 2a, b). Where the sponge tissues are thin at the periphery, archaeocytes can be seen crawling in the collagenous mesohyl (Fig. 2b, inset).

We used *silicatein M2,* a gene expressed in sclerocytes in the growing sponge as previously described in [24] as a positive control. At 2 and 5 dph expression of *silicatein M2* was detected in sclerocytes, which could be identified by their elongate shape (Fig. 2c, d), but never in amoeboid cells at the periphery. In 5 dph sponge, *silicatein M2* probe was also detected elsewhere, however it is unclear whether it is cellular or extracellular, or if these were cells fated to become sclerocytes (Fig. 2c, d). In contrast, *wnt* mRNAs were only detected in cells of 5 dph sponges that had a complete aquiferous system. In these sponges *wntA*, *B,* and *C* mRNA was detected in amoeboid cells in the mesohyl at the periphery of the sponge and throughout the choanosome (Fig. 2e-g). Probe targeting *β-cat* was detected in similarly-shaped amoeboid cells along the periphery of 5 dph sponges (Fig. 2h), however these cells lacked the thin filopodial-like projections seen in cells labelled by *wnt* probes. In all cases *wnt*s and *β-catenin* were detected in only a subset of amoeboid cells at the periphery. Double in situ hybridizations suggest that *wntA* and *C* might be co-localized in cells but *wntA* and *B* are not (Fig. 2i, j). We made a custom antibody to a fusion protein of a conserved region of sponge β-catenin (Additional file 6). Interestingly this custom antibody also labelled amoeboid cells in the mesohyl in *Spongilla lacustus* (Fig. 2k). The antibody labelled both the cytoplasm, regions around the cell membrane, as well as distinct filopodia. Images from Fig. 2f–k are shown as separate channels in Additional file 7.

**Fig. 3:**
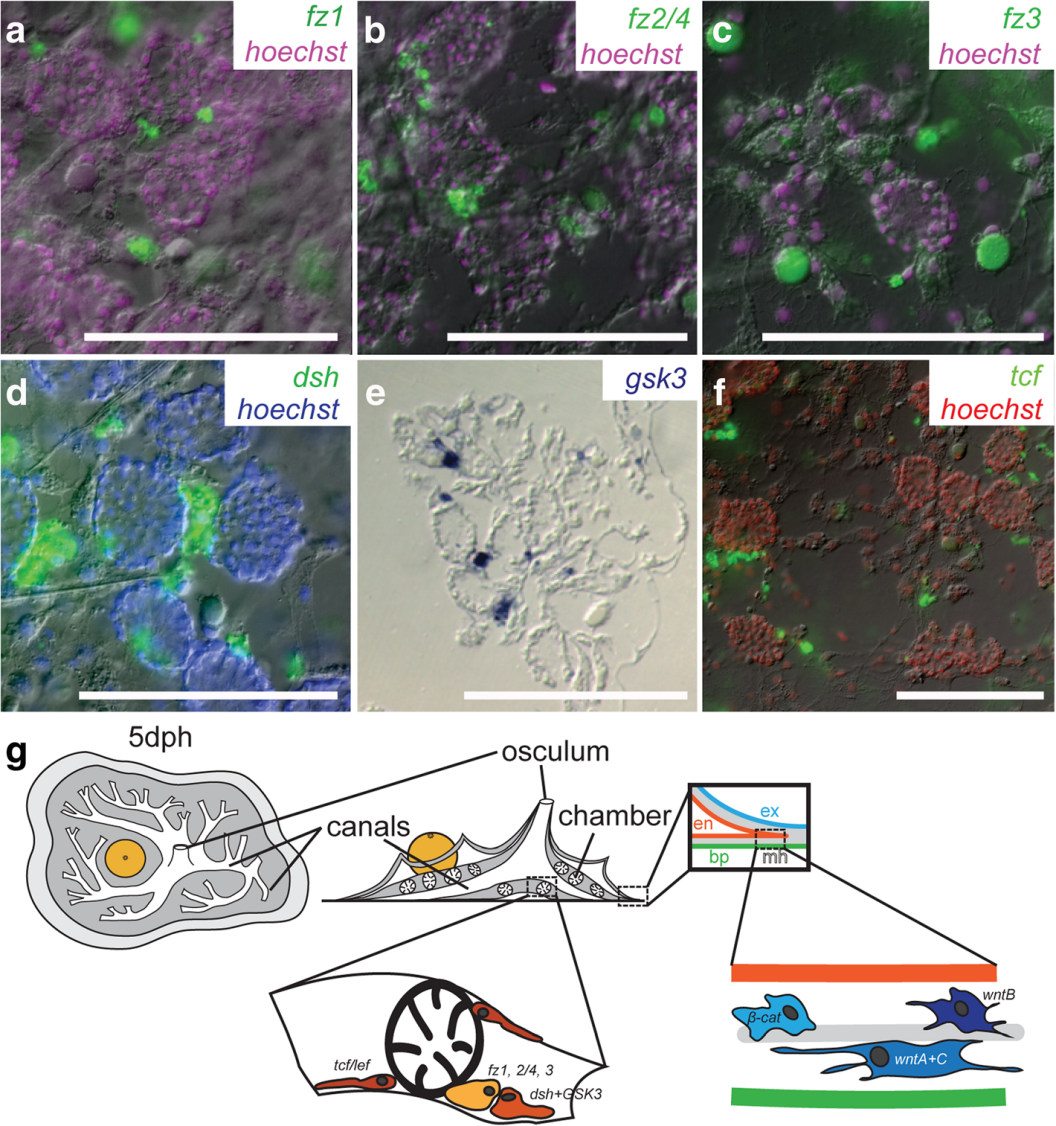
Expression of other Wnt pathway genes: *fz1, fz2/4, fz3, dsh, gsk3, tcf.* **a**-**d**), **f**) *fz1*, *fz2/4*, *fz3*, *dsh* and *tcf* mRNA expression, respectively (green), from fluorescent in situ hybridization with nuclear counterstain (magenta, blue or red, Hoechst) near choanocyte chambers. **e**) *gsk3* expression in the same region, visualized with NBT/BCIP and sectioned in epoxy. **g**) Summary diagram of the sponge body plan (top view, left; side view, right) at 5 dph with cell types and regions shown where Wnt pathway gene expression was detected. The small inset on the periphery shows layers of different cell types and linings of sponge regions: the exopinacoderm (ex, blue), the endopinacoderm (en, red) and basopinacoderm (bp, green), between these layers is the mesohyl (mh, gray) which contains many cell types including archaeocytes. In the choanosome (inset, right), we find expression of *fz*, *dsh*, *gsk3* and *tcf*. In the periphery (inset, left) we find *wnts* and *β-cat*. Modified from [61]. Scales: 100 μm

Probes targeting other Wnt pathway genes (*fz*, *dsh*, *GSK-3*, and *tcf/lef*) labelled cells in the mesohyl around the choanocyte chambers (Fig. 3a-f), but it was not possible to determine exactly what cells these were. The relationships between various cell types and regions is illustrated in Fig. 3g. We detected *wnt* and *β-cat* mRNA in amoeboid cells of the periphery, and *fz*, *dsh*, *GSK3* and *tcf/lef* mRNAs were detected in cells within the choanosome.

Interestingly, we found that in all sponges with a fully developed aquiferous system (5 dph) every probe, including non-sponge controls such as *Danio rerio hemoglobin* and *engrailed* as well as sense probe controls, labelled a region adjacent to choanocyte chambers (Additional file 8). Because no probe negative controls showed no label, the non-specific label above cannot be due to a lack of blocking endogenous alkaline phosphatases or peroxidase activity (Additional file 8).

### RNAi downregulation of GSK-3

**Fig. 4:**
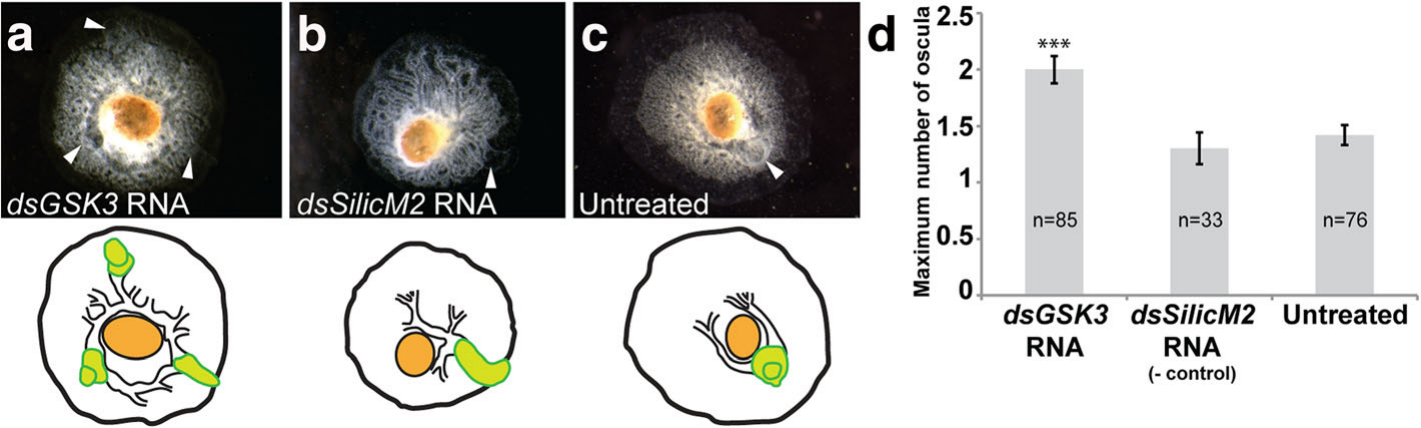
GSK3 knockdown with dsRNA causes multiple oscula in *Ephydatia muelleri*. **a** Treatment with 10 μg/ml *GSK3* dsRNA causes multiple oscula (arrowheads) to arise. **b** *silicatein M2* dsRNA treated sponges (negative control) develop with a single osculum. **c** Untreated sponges grown in 1× M-medium alone typically develop 1 osculum. Lower panel show illustrations to clarify the position of canals in black and oscula in green. **d** The mean maximum number of oscula for each treatment. *** indicates *p* < 0.005 (Dunn test); difference between controls was not significant (*p* > 0.05). Bars = standard error

Given that inhibition of GSK-3 protein by lithium chloride and alsterpaullone was shown to result in the formation of ectopic oscula in *E. muelleri* [22], we tested whether knockdown of *GSK-3* RNA by dsRNA would lead to similar changes in the aquiferous system (i.e., production of additional oscula). *GSK-3* dsRNA-treated *E. muelleri* sponges often developed multiple (2–5) oscula, and canals radiated from the center of the sponge in an irregular branching pattern (Fig. 4a). Untreated sponges and sponges treated with control dsRNA (for *silicatein M2,* a gene involved in spicule formation) developed a normal canal system with bifurcating canals and typically only one osculum (Fig. 4b, c), although occasionally two oscula were observed. The osculum rises away from the sponge roughly 100 μm or more and so diagrams are used to illustrate both the location of oscula and the branching pattern of canals attached to each (Fig. 4a-c).

As sponges hatched at different times, and because previous experiments had shown that extra oscula generated by lithium chloride treatment are eventually resorbed [22], we counted oscula several times over the 48 h treatment and recorded the maximum number of oscula observed (Fig. 4d). Overall, the data showed significant differences in the number of oscula (Kruskal-Wallis test: test statistic = 19.430, df = 2, *p* < 0.0005; Chi-Square test: critical value = 23.73, χ^2^ = 9.488, α = 0.05, v = 4, *p* < 0.01) and the maximum number of oscula observed in *GSK-3* dsRNA treated sponges was significantly higher than in sponges untreated or in *silicatein M2* dsRNA treated sponges (Dunn test: *n* = 85, *p* ≤ 0.005, k = 3). The number of oscula in untreated (*n* = 76) and *silicatein M2* dsRNA treated (*n* = 33) sponges was not significantly different (Dunn test: *p* > 0.05). A 28% knockdown of *EmuGSK-3* for this experiment was confirmed with two internal replicates by qPCR, using *EmuEf1-α* as the housekeeping reference gene (Additional file 9). Though the RNAi technique as currently used in *E. muelleri* only results in knockdown of ~30% (range of ~20–50% depending on the gene tested), phenotypes of *EmuGSK-3* knockdown sponges were distinct from those observed in other knockdown experiments targeting different genes [25–27].

### Pharmacological inhibition of Wnt pathway components

We treated hatching and regenerating *S. lacustris* with Wnt pathway inhibitors to test whether they would inhibit the formation of oscula. If inhibition of the negative Wnt signaling regulator GSK-3 causes the formation of ectopic oscula, then inhibiting positive regulators of Wnt signaling should prevent oscular development. Niclosamide has been shown to impede Wnt signaling by causing internalization of the Frizzled receptor, a reduction of dishevelled protein expression, loss of β-catenin stabilization and a reduction in transcription of Wnt target genes by TCF [28–30]. Park et al. [30] showed that quercetin treatment decreased TCF transcriptional activity, either by acting directly on TCF or on β-catenin to inhibit their binding, thereby reducing Wnt target gene expression.

**Fig. 5:**
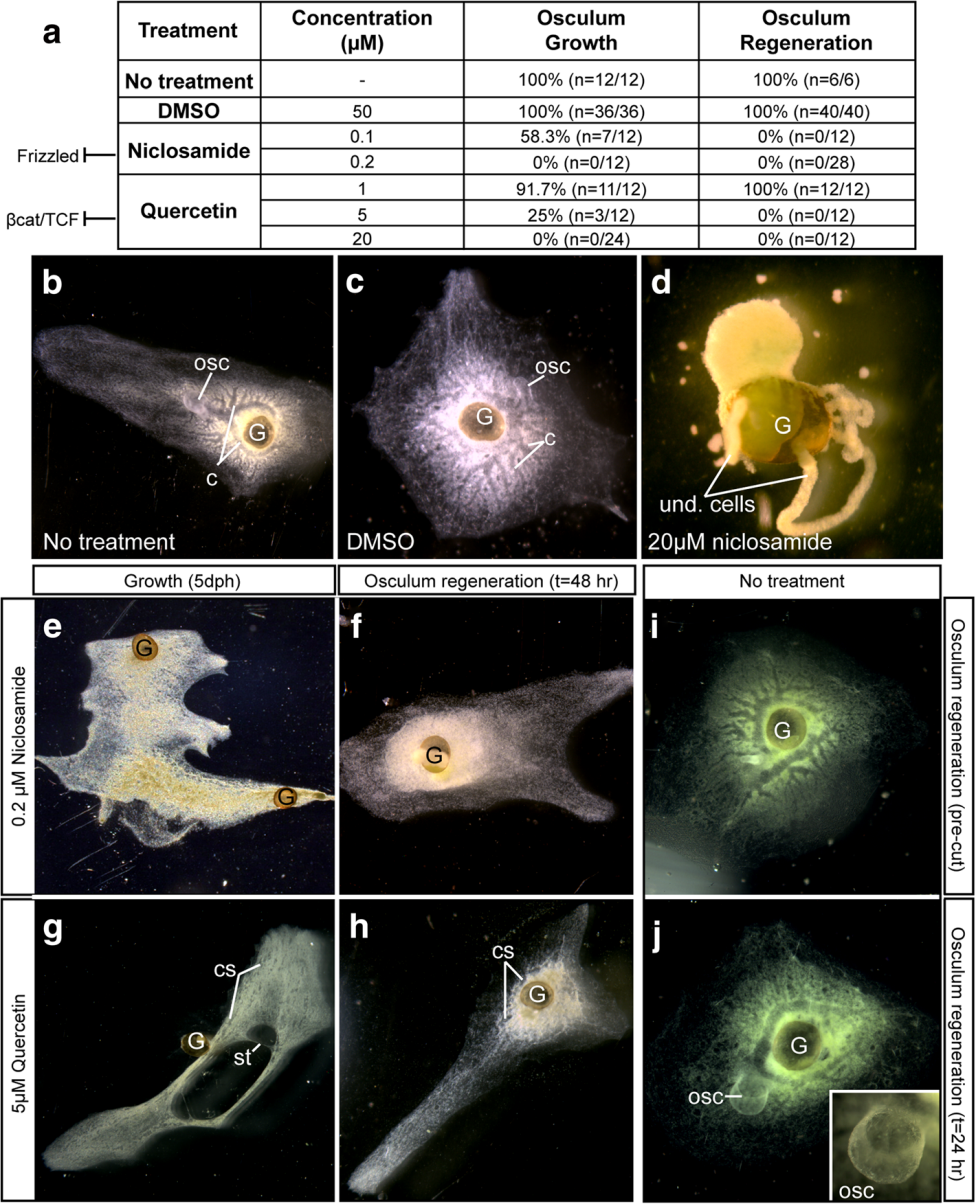
Pharmacological inhibition of Wnt signaling prevents development and regeneration of oscula in *Spongilla lacustris*. **a**) Table showing a summary of treatment results for osculum growth experiments and osculum regeneration experiments. **b**), **c**) No treatment and DMSO controls, respectively, showing normal gross morphology of a hatchling, with the gemmule husk (G), an osculum (osc) and canals (c). **d**) Catastrophic release of undifferentiated cells (und. Cells) after hatching in a high dose of niclosamide (20 μM). **e**) and **f**) Treatment with 0.2 μM niclosamide. **e**) When gemmules are hatched in niclosamide, oscula and canals do not form by 5 dph. **f**) When the osculum is removed from a normal sponge with subsequent niclosamide treatment, sponges fail to regenerate the osculum within 48 h, and also lose canals. **g**) and **h**) treatment with 5 μM quercetin. **g**) After 5 days of growth, quercetin also prevents osculum development, and canals do not form fully – instead they form canal spaces (cs) that appear distinct from one another in the tissue. These animals also grow and then recede, leaving behind a strand of tissue (st). **h**) Quercetin-treated animals fail to regrow oscula within 48 h and canals begin to degenerate into canal spaces (cs). **i**) and **j**) Osculum regeneration before removal (**i**) and after 24 h (**j**) with no treatment

Both niclosamide and quercetin blocked the formation of oscula during hatching and development and both drugs also prevented regeneration of oscular tissue after removal of the osculum (Fig. 5a). Effective concentrations of each drug were determined empirically and narrowed to 0.1–0.2 μM niclosamide and 5–10 μM quercetin; at these concentrations oscula were not formed. Control sponges (untreated or treated with 50 μM DMSO) developed and regenerated oscula normally (Fig. 5b–j). At high concentrations niclosamide treatment resulted in extreme phenotypes, and often the tissue simply formed a long tube of cells that did not appear to differentiate (Fig. 5d), however at 0.2 μM niclosamide sponges lacked oscula and canals but retained a more normal growth pattern (Fig. 5e). When we removed the osculum and treated the sponges with niclosamide, not only did the osculum fail to regenerate, but canal system organization was lost after 48 h (Fig. 5f).

The phenotype of sponges grown in quercetin was less severe than those grown in niclosamide, but at most doses sponges spread out to form mats containing lacunae but no fully-developed, branching canals. Often the sponges formed strands of tissue suggesting growth followed by tissue regression (Fig. 5g). At the highest concentration of quercetin, the osculum did not regenerate and organization of the aquiferous system was lost (Fig. 5h). For comparison, Fig. 5i and j show an untreated sponge at *t* = 0 and *t* = 24 h after osculum removal, respectively. The osculum grew back fully and in the same location (Fig. 5i, inset).

### The fate of larval polarity at metamorphosis

**Fig. 6:**
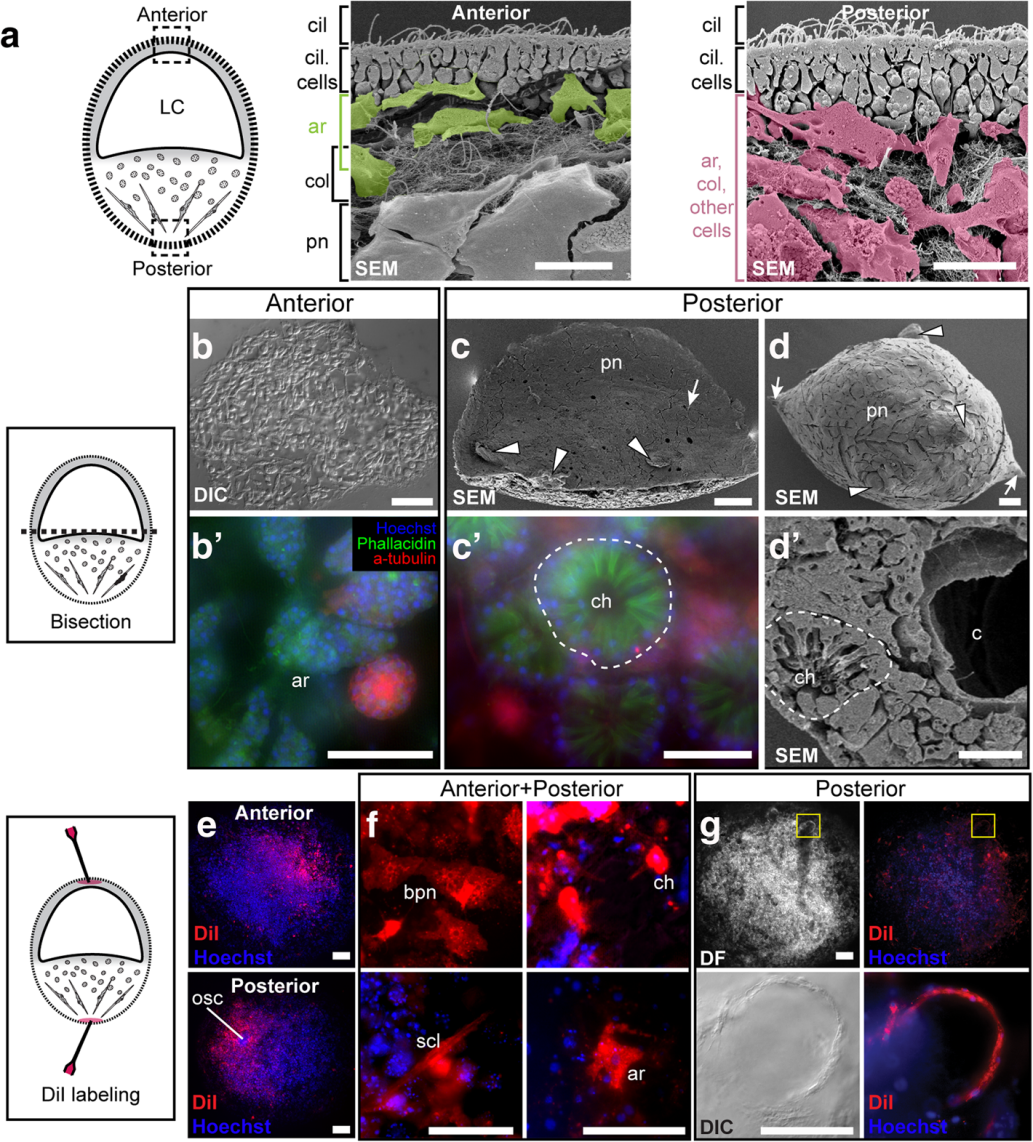
Bisection and diI labeling of *Eunapius fragilis* larvae: fate restrictions of anterior and posterior cells. **a** Body plan of a freshwater sponge larva. The anterior half of the larva is characterized by a larval cavity, and the posterior half contains a mixture of differentiated and undifferentiated cells. Boxed areas, insets: scanning electron micrographs (SEM, scales: 10 μm) of fractures from the anterior half (left) and posterior (right), showing archaeocytes (ar, pseudo-coloured green or pink), collagen (col) and other cells present throughout the larva. The surface of the larva is covered in small ciliated cells (~2 × 6 μm; cells with cilia, cil). The larval cavity is lined by pinacocytes (pn). **b** A settled anterior hemisphere lacking an osculum, choanocytes and canals. **b**′) These settled anterior halves contained only archaeocytes (ar). **c**) A fractured settled posterior hemisphere showing a normal pinacoderm (pn) and ostia (arrow) with multiple oscula (arrowheads). **c**′) Normal chaonocyte chambers (ch) in a settled posterior hemisphere. **d**) Posterior halves also failed to settle in 50% of cases and remained floating; these had a normal pinacoderm (pn), multiple oscula (arrowheads), and a spicule skeleton (arrows). **d**′) Fractured specimen showing canals (c) and choanocyte chambers (ch). **b**′) and **c**′) nuclei = blue (Hoechst), actin = green (Bodipy fluorescein-phallacidin), tubulin = red (anti-tubulin, Alexa 594 2° antibody). **e** Overview of labelled cells from anterior or posterior diI tattoos. Cell lineage tracer dye (DiI) is shown in red, nuclei are blue (Hoechst). **f**) Cells from posterior and anterior poles of larvae are fated to become various cell types including basopinacocytes (bpn), choanocytes (ch), sclerocytes (scl) and archaeocytes (ar). **g**) Cells at the posterior pole give rise to the osculum (bottom panels show boxed areas; DF = dark field). Scales: **b**), **c**), **e**) and **g**) 100 μm; **b**′) and **c**′) and **d**) 25 μm; **d**′) 10 μm; and **f**) 50 μm. Diagrams are modified from versions appearing in [61]

Going into this work our hypothesis was that Wnt signaling was involved in establishing axial polarity and development of the osculum. Our attempts, however, at detecting expression of *wnt* genes in larvae via in situ hybridization were not successful. Therefore, in order to understand whether larval polarity was fixed and retained in the adult or whether tissues were entirely malleable and could reorganize themselves at metamorphosis, we carried out experiments to examine the fate of the poles of the freshwater sponge larvae. Larvae are not produced simultaneously by all colonies of freshwater sponges, and so we used larvae obtained from those individuals and species that were spawning in different years. Following release from adults, swimming larvae of *Eunapius fragilis* (*n* ≥ 100 each over two years) were bisected perpendicular to their anterior-posterior swimming axis and the halves were cultured separately. Of these, at least half did not settle on coverslips and so could not be followed by microscopy or were lost during processing; here we show the typical results. In *E. fragilis* anterior halves settled 20–30 min after cutting but failed to develop choanocytes and an aquiferous system (Fig. 6b, b′). In contrast, the posterior halves of *E. fragilis* larvae consistently developed multiple oscula with choanocyte chambers and canals, but only 50% of them settled and metamorphosed (Fig. 6c, c′, d, d′). Those that failed to attach continued swimming – with oscula – for up to 3 days (Fig. 6d). We did not test whether any of the oscula were functional. The same experiments another year with *S. lacustris* swimming larvae (*n* ≤ 10) gave the opposite results: anterior halves failed to settle and instead continued swimming without further differentiation, while posterior halves settled and grew into normal sponges (Additional file 10). Due to low numbers of available larvae, we were not able to further process or examine bisected larvae from *Spongilla lacustris*.

We injected the anterior and posterior poles of *E. fragilis* larvae (*n* ≥ 100) with the fluorescent cell marker diI and tracked the fates of labelled cells through metamorphosis. Only a portion of these were followed through metamorphosis (anterior *n* = 18; posterior *n* = 20) for the same reasons as listed above for bisection experiments. The bulk of diI labelled cells – anterior and posterior – typically remained in a diffuse area following larval settlement, but individual cells labelled with diI were found scattered throughout the settler in both cases (Fig. 6e). In both anteriorly and posteriorly labelled larvae, labelled cells differentiated into several different cell types including sclerocytes, choanocytes, archaeocytes and basopinacocytes in the settled sponge (Fig. 6f). However, we only observed cells from the posterior pole becoming cells of the osculum (Fig. 6g, posterior *n* = 3/20; anterior *n* = 0/18).

## Discussion

*Wnt* genes and the Wnt signaling pathway were key to the innovations in multicellular metazoan body plan organization [7]. However, several lines of evidence suggest that while individual Wnt pathway components originated in unicells (e.g. [7, 31]), canonical Wnt signaling as we understand it – both mechanistically and for its role in axial polarity – did not arise until after sponges split from the other animals.

Wnt pathway components are present in sponges, but *wnt* genes are conspicuously absent from glass sponges – class Hexactinellida, which have uniquely syncytial tissues – and sponge Wnts form subfamilies separate from those of non-sponge animal lineages. Although other work has shown localization of *wnt* mRNAs to oscula in some sponges, in our study of freshwater sponges, *wnt* expression showed individual cells expressing *wnt* genes throughout the mesohyl of the sponge. Our pharmacological and knockdown experiments are consistent with a role for Wnt signaling in formation of the aquiferous canal system, one identifier of body polarity in a sponge, however our larval manipulation experiments show that conservation of tissue polarity during ontogeny might differ even between sponge genera. Overall the data describe a picture of diverse function of Wnt signaling in sponges.

### Sponge Wnt relationships

The presence and absence of Wnts in sponges is an interesting study. We found that three out of four classes of sponges have wnt genes and most of the other major components of the pathway, with major duplications in some groups and probable losses in others.

First, we did not find any Wnts in our transcriptome of *Aphrocallistes vastus* (Hexactinellida). This result is echoed in a recent study using new transcriptome data from a second hexactinellid, *Oopsacas minuta*, which also lacks any *wnt* genes [32]. An interesting probability is that being syncytial, glass sponges may use signaling pathways such as Wnt independently of a ligand; if so, glass sponges could provide a unique model for studying body plan development. This interpretation also suggests that glass sponges may have lost Wnts after they diverged from other sponges, and a next important step is to confirm presence/absence of Wnts and Wnt pathway components in other glass sponges by way of a complete genome.

Second, each of the remaining sponge classes has its own complement of Wnts that have diversified within each lineage. Calcareous sponges have more than 20 Wnts [20]. We identified 13 Wnts in the *Sycon coactum* transcriptome and we were able to assign 11 of these to existing Calcareous sponge Wnts as defined in the above study. The number of Wnts in this class of sponges is surprising only because we tend to associate Wnt signaling with body plan complexity and sponges lack an obviously modular body with complex organs (reviewed in [33]). It is clear from existing data that the Calcarea may have undergone genome duplication events at some time in their evolutionary history causing the appearance of several paralogues of both signaling genes and transcription factors [20, 34]. The function of each of these paralogues or redundancy between them is presently unknown.

### Expression and function of Wnt in the freshwater sponge

No regional expression of Wnt pathway genes could be detected in *Ephydatia muelleri*; rather all genes showed a cellular expression pattern. The three *wnt* genes and *β-catenin* are expressed in archaeocytes in the mesohyl at the periphery of the sponge. Some of these archaeocytes appeared to be actively crawling with cytoplasmic extensions reaching out in many different directions. Similar expression was shown for *wnt* in *Ephydatia fluviatilis* suggesting involvement in spicule organization and arrangement [35], where it was suggested the cells expressing *wnt* are involved in organizing the positions of spicules. We found no specific co-localization of *wnt* expression with spicules in our observations. Alternatively, these archaeocytes may be involved in seeking out directions in which the sponge can grow and spread, as this species is an encrusting sponge. Wintermann [36] described two types of crawling archaeocytes, those with and those without filopodia, and these two types respectively express *wnt* and *β-catenin* in *Ephydatia muelleri* (e.g. [23]).

Based on existing functional and expression work we expected to find *wnt* expressed in or around the osculum, or perhaps even the ostia [17–22], but we did not find osculum or ostium expression of any *wnt* or Wnt pathway gene in our sponges. Although it is possible that this is due to a true absence of expression, it is also possible that the tissue of the osculum did not withstand the physical processing of in situ hybridization. Specimens often lost their oscula during the procedure or were heavily damaged despite our best efforts to minimize forces. However, our data is supported by a recent study using a *β-catenin* antibody in *Ephydatia muelleri* that did not reveal staining in the osculum or ostia, but did localize to amoeboid cells as we observed, as well as to some focal adhesion-like structures and substrate-attachment epithelium [23].

Importantly, our work highlights the need to use several controls when applying well-known tools from model systems onto non-models. Our finding that all probes highlight a region adjacent to choanocyte chambers in 5 dph sponges suggests that some cells with inclusions, possibly algal symbionts here, may sequester RNA probes in sponges. Since identification of cell types is difficult in all sponges, and most sponges have cells with inclusions and many also have algal or cyanobacterial symbionts, care must be taken when interpreting regions showing strong labelling in cells with inclusions in sponges. Furthermore, different sponge species have distinct biologies and this should also be taken into account. Clearly a better understanding of sponge cell biology and function will be critical in interpreting how genes work in the sponge.

### ‘Polarity’: A role for Wnts in establishment of sponge axis

In earlier work we reasoned that the osculum was a definitive marker for sponge ‘polarity’ [22]. We found that chemicals that activated Wnt signaling in other animals caused the formation of multiple oscula in the freshwater sponge *Ephydatia muelleri*. We followed this up here using *GSK-3* knockdown experiments, and found more oscula formed, as well as pharmacological inhibition of Wnt signaling using quercetin and niclosamide, both of which inhibit Wnt/Frizzled signaling, and found a concentration-dependent inhibition of oscula and aquiferous system development. These results suggest to us that some aspects of the canonical Wnt pathway are involved in formation of the aquiferous system in sponges, but it is not clear to us that this sort of polarity can be properly compared to anterior-posterior polarity in other metazoans.

An important caveat to our pharmacological treatment data is that the actions of these two drugs are not specific to Wnt pathway proteins. Quercetin has many targets including regulators of cell cycle genes and apoptosis, which may explain its role in reducing the growth of cancer cells in rats and cancer cell lines [37, 38]. Similarly, niclosamide does not exclusively work on Frizzled proteins; it has been shown to inhibit other cell signaling molecules in various types of cancers and other diseases (ERK, Notch, JAK-STAT, NF-κβ etc.) (Reviewed in [39]). Further studies are required to confirm that the drugs affect the Wnt pathway in sponges.

Sponge larvae have overt anterior-posterior (A-P) swimming polarity [40, 41], and regional expression of some *wnt* genes along the A-P axis in *Sycon ciliatum* [20] and at the posterior pole of *Amphimedon* [18] and the osculum of *Halisarca dujardini* [21] strongly hints at a role for those Wnts in polarity. However, different *wnts* are expressed in the posterior pole of the *S. ciliatum* larva than in the osculum of the juvenile sponge, and such a range of expression patterns is seen (in other cells and other regions of the sponge) that it is difficult to know whether polarity is carried over during metamorphosis or re-established de-novo in the juvenile and adult. Our experiments using injection of dye into cells at the posterior and anterior poles of the larva show that in *Eunapius fragilis* certain elements of larval A-P polarity carries through to the juvenile stage, and more specifically cells at the posterior pole of the larva form the osculum of the juvenile. This is the first confirmation of the conservation of polarity through metamorphosis in a demosponge using cell tracking methods and the finding is significant because the posterior pole of the *Amphimedon* sponge larvae also expresses *wnt* genes, suggesting that Wnts are involved in determining polarity in sponges as they are in other animals [17, 18, 20]. But we still do not know if gene expression is maintained through metamorphosis. We did not have larvae from *Ephydatia muelleri* to study and our ISH experiments with larvae from *Eunapius fragilis* showed no expression, therefore no conclusions regarding the involvement of Wnt signaling and freshwater sponge larval polarity can be drawn.

## Methods

### Collection and culture of freshwater sponges

Adult sponges were collected from lakes near Bamfield, BC for experiments at the Bamfield Marine Sciences Centre and at the University of Alberta. For the RNAi experiment, gemmules were collected in Helena, MT from the site described in [42] and stored at the University of Richmond (Richmond, VA, USA). Specimens at the University of Richmond were imaged on an Olympus SZX12 Stereomicroscope, using ProgRes Camera and the ProgRes Capture software.

Gemmules of *S. lacustris* and *E. muelleri* were collected in December and stored at 4 °C in lake water until use. Gemmules were cleaned of the adult skeleton, treated with hydrogen peroxide and hatched in 1× M-medium consisting of 0.1 mM CaCl_2_, 0.05 mM MgSO_4_, 0.05 mM NaHCO_3_, 0.005 mM KCl and 0.025 mM Na_2_SiO_3_ pH 7.0 [43, 44].

Adult specimens of *E. fragilis* were collected in July 2012 and 2013 from Frederick Lake, near Bamfield, BC. Two to five sponges were kept in 20 L of lake water at 15–18 °C and refreshed every 1–2 days. Larvae released from the adult sponge were collected from the surface of the water using a flashlight and a glass Pasteur pipette, and transferred to dishes containing 0.22 μm filtered lake water.

General observations of live animals were recorded using a Nikon Coolpix 995 digital camera or a QI-cam mounted on a Zeiss SZX12 Axioskop stereomicroscope. Images were cropped, resized and assembled using Adobe Photoshop and Illustrator CS6.

### Transcriptome and phylogenetic analysis

We used BLAST+ [45] to search our own transcriptomes from 10 species of sponges representing all 4 poriferan classes (from [3] and unpublished data) for Wnt pathway components. The *Aphrocallistes vastus*, *Ephydatia muelleri* and *Eunapius fragilis* transcriptomes were sequenced with a HiSeq2000 at LC Sciences (http://www.lcsciences.com; Houston, TX), while those for *Spongilla lacustris*, *Petrosia ficiformis*, *Pseudospongosorites suberitoides*, *Ircinia fasciculata*, *Chondrilla nucula*, *Sycon coactum* and *Corticium candelabrum* were sequenced using the Illumina GAII and HiSeq2000 at the Harvard University FAS Center for Systems Biology (Cambridge, MA). Libraries were prepared using the TruSeq RNA sample preparation kit (Illumina, Inc.) and sequences were assembled in CLC Genomics Workbench 5.1 (CLC Bio) (CLC Bio, Aarhaus, Denmark). Other sequence data included in alignments were obtained from online repositories, as listed in Additional file 1.

We constructed phylogenetic trees for both sponge Wnt proteins alone and several metazoan Wnts; sequence accession numbers for phylogenetic analysis are provided in Additional file 11. Only sequences deemed to be full-length or almost full-length were used in phylogenetic analysis. Sequences were aligned using Mafft [46] and trimmed using TrimAL with the ‘Automated 1’ setting [47]. We constructed 3 types of maximum likelihood trees using PhyML (http://www.atgc-montpellier.fr/phyml/; [48], RAxML Blackbox (http://embnet.vital-it.ch/raxml-bb/index.php; [49] and IQ-TREE (http://iqtree.cibiv.univie.ac.at/; [50]. The final alignment was analyzed using ProtTest 3.4.2 and LG + G + I was determined to be the model that gave the best log likelihood value (−lnL = 34,070.97) and thus was used in all analyses.

For the PhyML tree, we created a starting BIONJ tree with SPR tree improvement from 5 random starting trees. Support was measured with 1000 bootstrap replicates. This method was considered the most rigorous one. A RAxMLtree was done with 100 bootstrap replicates and the IQ-TREE with 1000 ultrafast bootstrap replicates [51]. A consensus tree was created including bootstrap support values for each analysis. Nodes with support values below 50 were collapsed into polytomies.

### Gene expression

Expression of Wnt pathway genes was studied in 2 and 5 days post hatch (dph) sponges grown from gemmules of *Ephydatia muelleri* using in situ hybridization. Sponges were grown in M medium and fixed in 4% paraformaldehyde (PFA) in ¼ Holtfreter’s solution (HS) (15 mM NaCl, 0.2 mM KCl, 0.2 mM CaCl_2_, 0.6 mM NaHCO_3_; [52]) with 0.03% glutaraldehyde overnight at 4 °C. Sponges were rinsed in ¼ HS, dehydrated to 100% ethanol and stored at −80 °C until ready to use. Tissue was rehydrated and permeabilized with 5 μg/mL Proteinase K for 1–2 min, and post fixed in 4% PFA in PBS with 0.1% Tween-20 before hybridization.

Genes were cloned into the pGEM-T vector (Promega); primers are listed in Additional file 12. Using a PCR-generated template, reverse transcription was carried out using 10× RNA-DIG or 10× RNA-biotin labelling mix and T7 RNA Polymerase (Roche) to generate antisense and sense probes. The products were precipitated with lithium chloride and ethanol and stored at −20 °C containing 1 unit RNaseOUT (Invitrogen). Concentration ranges for probes were determined empirically using blots.

Probes were hybridized to sponge tissue for 16–72 h at 55 °C in hybridization buffer (50% formamide, 5× SSC, 50 μg/mL heparin salts, 100 μg/mL Torula yeast tRNA, 5× Denhardt’s Solution and 0.1% Tween-20). Post-hybridization washes of 20 min each were performed at hybridization temperature using post-hybridization solution (50% formamide, 5× SSC and 0.1% Tween-20) in 2× SSC, pH 4.5, at a ratio of 3:1, 1:1 and 1:3 respectively, with a final set of 3 × 20-min wash in 2× SSC pH 4.5. Detection of probes was performed using alkaline phosphatase (AP) conjugated anti-DIG antibody (1:200; Roche), POD conjugated anti-DIG antibody (1:200; Roche), or streptavidin (1:1000; Roche) with Alexa 488 or 594 tyramide reactions. All antibody incubations were performed overnight at 4 °C on a shaker. Tissue was rinsed in maleic acid buffer (10 × 30 min), and colour reactions were performed in the dark at room temperature from 3 h to overnight depending on probes used. Specimens were labelled with Hoechst 33,342 for nuclei and mounted in Mowiol.

Slides were viewed and imaged on a Zeiss Axioskop 2 Plus compound microscope with a QImaging camera running Northern Eclipse (Empix).

### Antibody preparation and labeling

An antibody to *S. lacustris* was prepared by injecting rabbits with a fusion protein composed of a intein-chitin tag and residues 1–183 of the *S. lacustris* β-catenin protein, which showed 88.4% sequence identity (97.4% similarity) with the *E. muelleri* β-catenin protein. The sequence encoding the fusion protein was constructed using the IMPACT kit (New England Biolabs). After expression in *Escherichia coli* the fusion protein was purified on a chitin column according to the manufacturer’s instructions and dialyzed against PBS containing 1× cOmplete EDTA-free proteinase inhibitor cocktail (Sigma). Peptides were analyzed by mass spectroscopy to confirm sequence identity and injected into rabbits without further processing. The first injection was done in Freunds Complete Adjuvant, and a subsequent three injections in Freunds Incomplete Adjuvant (Sigma).

Serum samples were analyzed by a combination of ELISA and Western blotting. Sponges were fixed and labelled with antibody as described previously [53].

### RNA interference

Double stranded RNA (dsRNA) was generated according to the method used in [25] for *EmuGSK-3* and *EmuSilicateinM2.* Primers are listed in Additional file 11. Gemmules from *E. muelleri* were hatched in 1× M-medium in 12-well dishes as described above. Shortly after hatching 10 μg/mL dsRNA was added to cultures and fresh solution was exchanged every 24 h throughout the course of the experiments.

Phenotypes were observed by stereomicroscopy and oscula were scored every 24 h over 2 days, and confirmed with blind counting of oscula number by a second individual. Imaging was carried out simultaneously. Ostia were not counted as this would require electron microscopy, and inhibition of GSK-3 protein by lithium chloride in this species did not reveal additional ostia [22]. Osculum count data were not normally distributed and had unequal variances, so we used the non-parametric method of Kruskal-Wallis to test for significant differences in the datasets and also confirmed using a Chi Square test. A Dunn test was used to determine difference between treatments [54].

Sponges were stored in RNA Later (Invitrogen) and frozen at −80 °C. RNA was extracted using the RNeasy kit (Qiagen). Knockdown of GSK-3 was confirmed with qPCR, using *EmuEf1-α* as a control as described in [25].

### Pharmacological experiments

We treated *Spongilla lacustris* with niclosamide and quercetin (Sigma-Aldrich) during hatching and growth as well as in osculum regeneration experiments. Each chemical was dissolved in dimethyl sulfoxide (DMSO) to make 10 mM stock solutions. Effective concentrations were determined empirically at a minimum of 0.1 μM for niclosamide and 10 μM for quercetin in 1× M-medium. DMSO controls were conducted at up to 50 μM in 1× M-medium.

The presence or absence of an osculum and the number of oscula per dish were recorded for a given number of sponges. In osculum regeneration experiments, sponges were grown in 1× M-medium until oscula developed (5–6 dph). Oscula were manually removed at their bases, and sponges were placed into the respective treatments. Oscula were counted at 7 h and 1 day post osculum removal.

### Larval bisection experiments

Swimming larvae of *Eunapius fragilis* were collected before settlement and immobilized by capillary action along the edge of a microscope slide in a Petri dish. Larvae were bisected at their midpoint, perpendicular to the anterior-posterior axis using a 15°, 5 mm restricted depth, straight stab knife (Fine Science Tools) into anterior and posterior hemispheres. These were fixed for electron and fluorescence microscopy immediately or 48 h after bisection.

To label anterior and posterior poles of the larva 75–100 nL of diI prepared at a concentration of 2 mg/mL in DMSO was injected using an Eppendorf Femtojet into each larva (after [55]). Sponges were cultured to the juvenile stage (24–48 h) and fixed for fluorescence microscopy.

## Conclusions

The cellular expression of *wnt* and Wnt pathway genes in freshwater sponges initially puzzled us, but the diversity of experiments we have carried out now force us to conclude that Wnt signaling does occur in sponges but its role is far more complex than simply designating regionalization of structures. Wnt is likely expressed by cells that act only within a localized region to direct sets of cells for a particular role. Whether those cells will form the osculum, ostia, or pattern canal formation may depend on the lineage of sponge. The absence of Wnts in the glass sponge *Aphrocallistes vastus* is noteworthy. Glass sponges are syncytial, an unusual tissue construction in which nuclei move through giant syncytia, and in which cellular regions (including archaeocytes) are non-motile and anchored against flagellated chambers. Glass sponges are highly polarized as larvae and as adults and yet if Wnts are truly absent, this polarity is formed and maintained either with a substitute ligand or without Wnt signaling. In either case, it implies that canonical Wnt signaling is not required for polarized larval or adult tissue and body organization in sponges.

A fruitful line of study will be identifying the aspects of sponge body organization that Wnt pathway components are required for, possibly organizing the position of skeletal components, regions of the canal system, or simply choreographing the function of specific sets of cells (e.g., cell adhesion, cell motility). The recent description for a role of the non-canonical Rho-Rock Wnt signaling module in the establishment of the *Ephydatia muelleri* aquiferous system supports this notion [56]. It is likely that some of these roles are used in other metazoans, reflecting a deep homology of the Wnt signaling pathway. It would be particularly interesting to know whether key interactions between fundamental components of the Wnt pathway pre-date and formed the first steps in metazoan evolution.

## Additional files

Additional file 1: List of sources of sequence data obtained for phylogenetic analysis. (PDF 81 kb)

Additional file 2: Mafft alignment of sponge Wnt protein sequences prepared in Boxshade (http://www.ch.embnet.org/software/BOX_form.html). Conserved cysteine residues are marked with a red asterisk, and the conserved RWNC motif is indicated with a green bracket. (PDF 9686 kb)

Additional file 3: Raw phylogenetic trees used to create the consensus tree presented in Fig. 1. A) PhyML tree with support values from 1000 bootstrap replicates. B) RAxML tree showing bootstrap support from 100 replicates. C) IQ-TREE with support values from 1000 SH-aLRT replicates/aBayes/1000 ultrafast bootstrap replicates. (PDF 820 kb)

Additional file 4: Assignment of sponge Wnts to each sponge-specific Wnt subfamily. Groupings were decided by several phylogenetic analyses and are taken from the consensus tree shown in Fig. 1, and subfamily names were retained from [18–20]. (PDF 65 kb)

Additional file 5: Raw phylogenetic PhyML tree with sponge and bilaterian Wnt sequences. Values displayed are bootstrap support from 100 replicates. Species codes: Nve = *Nematostella vectensis*, Dre = *Danio rerio*, Hsa = *Homo sapiens*, sponge species codes are as listed in the legend of Fig. 1. (PDF 585 kb)

Additional file 6: Western blot validation of our custom antibody to a β-catenin fusion protein from *Spongilla lacustris*. (PDF 349 kb)

Additional file 7: Fluorescent in situ hybridization and antibody images showing separate channels for images shown in Fig. 2. A) *wntB*, B) *wntA*/*wntC*, C) *wntA*/*wntB* and D) β-catenin antibody. (TIFF 6439 kb)

Additional file 8: In situ hybridization control experiments. A) A dual probe from *Danio rerio* against *hemoglobin* (*hb*) and *engrailed* (*en*) shows a brightly labelled region next to choanocyte chambers (arrowhead). B) Dual probe from *D. rerio* co-labelled with a *dsh* probe, showing the same regions labelled next to chaonocyte chambers (arrowhead). Co-staining indicates a lack of full specificity of the sponge *dsh* probe. C) Low magnification view of a sense probe control for *silicatein M2* (*silcM2*). The boxed area is shown to the right at higher magnification showing diffuse, low-level staining of many cells and structures, including spicules. D) Fluorescent *silcM2* label for comparison showing brightly labelled sclerocytes surrounding spicules, and no background staining. Boxed area is shown to the right at a higher magnification. E) Nuclei labelled with hoechst (left), tissue autofluorescence when no probe is applied (middle), and a DIC overview of the region pictured (right). Scales = 50 μm. (TIFF 7044 kb)

Additional file 9: Confirmation of *gsk3* knockdown by qPCR. Relative expression levels of *gsk3* in untreated versus dsRNA treated sponges (dsRNA *EmuGSK3*). Expression levels normalized to *Ef1-α*. (TIFF 319 kb)

Additional file 10: Bisection experiments in *Spongilla lacustris* larvae have the opposite result of that seen in *E. fragilis*. Larval appearance is very similar to *E. fragilis*. However, in *S. lacustris* the posterior hemisphere settles and forms a normal sponge while the anterior half remains undifferentiated and continues swimming in the water column for up to 3 days. (TIFF 2871 kb)

Additional file 11: Accession numbers of previously published sequences used for phylogenetic analysis in Fig.1 and Additional files 3 and 5. (PDF 64 kb)

Additional file 12: Primers used to amplify sequences for RNAi and mRNA injection experiments. (PDF 66 kb)

## Abbreviations

*Aar*: *Aardvark*
AP: Alkaline phosphatase
A-P: Anterior posterior
BIO: 6-bromoindirubin-3′-oxime
BLAST: Basic Local Alignment Search Tool
DIG: Digoxigenin
diI: 3H–Indolium,2-[3-(1,3-dihydro-3,3-dimethyl-1-octadecyl-2H-indol-2-ylidene)-1-propenyl]-3,3-dimethyl-1-octadecyl-,perchlorate
dkk: dickkopf
DMSO: Dimethylsulfoxide
dph: days post hatch
dsh: dishevelled
dsRNA: double-stranded RNA
Ef1α: Elongation factor 1-alpha
ERK: Extracellular-signal regulated kinase
FISH: Fluorescent in situ hybridization
Fz: Frizzled
GSK-3: Glycogen synthase kinase 3
HS: Holtfreter’s Solution
JAK-STAT: Janus Kinase-Signal Transducer and Activator of Transcription
mRNA: messenger RNA
NBT/BCIP: Nitroblue tetrazolium salts/5-bromo-4-chloro-3-indolyl-phosphate
NF-κβ: Nuclear factor kappa-light-chain-enhancer of activated B cells
PBS: Phosphate buffered saline
PCR: Polymerase chain reaction
PFA: Paraformaldehyde
POD: Peroxidase
qPCR: quantitative PCR
RNA: Ribonucleic acid
RNA: RNA sequencing
RNAi: RNA interference
SSC: Saline-sodium citrate
TCF-LEF: Transcription factor/lymphoid enhancing-binding factor proteins
tRNA: transfer RNA
Wnt: Wingless/integrated superfamily proteins
β-cat: β-catenin
