# Supplementary material for: Wnt signaling and polarity in freshwater sponges
Source: BMC Evol Biol. 2018 Feb 2;18:12. doi: 10.1186/s12862-018-1118-0 (PMC5797367; doi:10.1186/s12862-018-1118-0)
Supplement: Supplementary file 1 — List of sources of sequence data obtained for phylogenetic analysis. (PDF 81 kb) [file 12862_2018_1118_MOESM1_ESM.pdf]

Additional File 1

Class	Species	Data type	Reference	Source
**Hexactinellida**	*Aphrocallistes vastus*	Transcriptome	Riesgo et al 2014	https://doi.org/10.7939/R3S000
**Demospongiae**	*Ephydatia muelleri*	Transcriptome	n/a	Unpublished
	Spongilla lacustris	Transcriptome	Riesgo et al 2014	GenBank BioProject PRJNA225591
	Eunapius fragilis	Transcriptome	n/a	Unpublished
	Petrosia ficiformis	Transcriptome	Riesgo et al 2014	GenBank BioProject PRJNA162901
	Chondrilla nucula	Transcriptome	Riesgo et al 2014	GenBank BioProject PRJNA225590
	Ircinia fasciculata	Transcriptome	Riesgo et al 2014	GenBank BioProject PRJNA225586
	Pseudospongosorites suberitoides	Transcriptome	Riesgo et al 2014	GenBank BioProject PRJNA225580
	Crella elegans	Transcriptome	Perez-Pollo et al 2013	http://dx.doi.org/10.5061/dryad.50dc6/4
	Halisarca dujardini	Transcriptome	Borisienko et al 2016	http://www.ebi.ac.uk/ena
	Amphimedon queenslandica	Accessioned sequences	Adamska et al 2007, 2010	https://www.ncbi.nlm.nih.gov/
**Homoscleromorpha**	*Corticium candelabrum*	Transcriptome	Riesgo et al 2014	GenBank BioProject PRJNA162903
	Oscarella carmela	Transcriptome	n/a	http://www.compagen.org/
	Oscarella lobularis	Accessioned sequence	Lapebie et al 2009	https://www.ncbi.nlm.nih.gov/
**Calcarea**	*Sycon ciliatum*	Accessioned sequences	Leininger et al 2014	https://www.ncbi.nlm.nih.gov/
	Leucosolenia complicata	Transcriptome	Fortunato et al 2012	http://www.compagen.org/
	Sycon coactum	Transcriptome	Riesgo et al 2014	GenBank BioProject PRJNA162899
