# Supplementary material for: Wnt signaling and polarity in freshwater sponges
Source: BMC Evol Biol. 2018 Feb 2;18:12. doi: 10.1186/s12862-018-1118-0 (PMC5797367; doi:10.1186/s12862-018-1118-0)
Supplement: Supplementary file 2 — Mafft alignment of sponge Wnt protein sequences prepared in Boxshade (http://www.ch.embnet.org/software/BOX_form.html). Conserved cysteine residues are marked with a red asterisk, and the conserved RWNC motif is indicated with a green bracket. (PDF 9686 kb) [file 12862_2018_1118_MOESM2_ESM.pdf]

# Additional File 2

```
AquWntC      1 MKIFS-------------------------------------------RHYC--------
PfiWntC      1 MRISIAKIM-----------------------------------------TC--------
HdujWntC     1 MT----------------------------------------------------------
AquWntB      1 MLCRG-------------------------------------------------------
PfiWntB      1 M-----------------------------------------------------------
EmuWntB      1 MRMCECT-----------------------------------------------------
SlaWntB      1 MRMSESI-----------------------------------------------------
EfrWntB      1 MRMSELV-----------------------------------------------------
AquWntA      1 MAFTS-------------------------------------------------------
EmuWntA      1 ------------------------------------------------------------
SlaWntA      1 MDKRAVQ-----------------------------------------------------
EfrWntA      1 MDKKAVQ-----------------------------------------------------
CreWntA      1 MSLCT-------------------------------------------------------
PsuWntA      1 MA----------------------------------------------------------
CcaWntX1     1 M-----------------------------------------------------------
HdujWntL     1 MLLSDMDRGRT-------------------------------------------------
OloWntI      1 TRPA--------------------------------------------------------
OcaWntI      1 MRV---------------------------------------------------------
CcaWntI      1 MPYSV----------------------------------------------------P--
LcoWntA      1 MNM---------------------------------------------------------
SciWntA      1 MDSC--------------------------------------------------------
ScoWnt-i     1 MEVCLVTF----------------------------------------------------
SciWntN      1 MTFVECTV----------------------------------------------------
ScoWnt-ii    1 MAYVSQAA----------------------------------------------------
SciWntT      1 MASSL----HTSSHLQ----------------------------------GC--------
LcoWntE      1 MYSLSMLAW---------------------------------------------------
SciWntE      1 MLLA--------------------------------------------------------
ScoWnt-iii   1 RVPL--------------------------------------------------------
LcoWntR      1 MSRSR---G---------------------------------------------------
SciWntR      1 MEQAQHVC----------------------------------------------------
LcoWntC      1 MAIR--------------------------------------------------------
SciWntC      1 M-----------------------------------------------------------
ScoWnt-v     1 M-----------------------------------------------------------
LcoWntI      1 MASSK--------------------------------------------FTC--------
SciWntI      1 MADG--------------------------------------------------------
ScoWnt-iv    1 MS----------------------------------------------------------
LcoWntJ      1 NTSGR-------------------------------------------------------
SciWntJ      1 MA----------------------------------------------------------
LcoWntG      1 MALLNM------------------------------------------------------
SciWntG      1 MANVG-------------------------------------------------------
ScoWnt-vi    1 MATMP-------------------------------------------------------
OcaWnt2X     1 ISVHSCLCMISRQ----------------------------------TRFLCF--VNP-A
EmuWntC      1 M-----------------------------------------------------------
SlaWntC      1 ------------------------------------------------------------
EfrWntC      1 MEGSASTAYSSLTAKI-------------------------------FSKLV--------
CreWntC      1 MKEGMSFSSSSLQRIA-----------------LKEK--------E-QARFC--------
CcaWntX2     1 MLVTHQSSDEQTD-----------------------------------------------
LcoWntD      1 MMAWTSLQRFT-------------------------------------------------
SciWntD      1 M-----------------------------------------------------------
ScoWntX1     1 MAQCPQGRR-------------W-------------------------------------
LcoWntF      1 MARNL-------------------------------------------------------
SciWntF      1 MH----------------------------------------------------------
LcoWntQ      1 FARFREANFLKPQAIG--------H-FVGCTKRSP----------S-FSSSC--------
SciWntQ      1 ------------------------------------------------------------
HdujWntD     1 MTPTARDAD---------------------------------------------------
HdujWntE     1 ------------------------------------------------------------
HdujWntF     1 MKSFLTEVN---------------------------------------------------
HdujWntH     1 MTSLF----------------------------------------------C--------
HdujWntI     1 MSRISPMSL---------------------------------------------------
HdujWntG     1 MTATA-------------------------------------------------------
OloWntII     1 TRPNRRSSN---------------------------------------------------
OcaWntIIa    1 MH----------------------------------------------------------
OcaWntIIb    1 SAPNSQQ-----------------------------------------------------
OcaWntIIc    1 MFIKR-------------------------------------------------------
OcaWnt5bX    1 M-----------------------------------------------------------
OcaWnt6X     1 MGGAPAATI---------------------------------------------------
LcoWntK      1 MIA---------------------------------------------------------
SciWntK      1 MSHSTMRSWYAVSEVRFLLL----------------------------------------
LcoWntU      1 ------------------------------------------------------------
SciWntU      1 MALSP-------------------------------------------------------
SciWntM      1 MRQQANRSS------------------------------------------CRE------
ScoWntX3     1 MS----------------------------------------------------------
LcoWntH      1 MALLLMTVG---------------------------------------------------
SciWntH      1 MGP---------------------------------------------------------
ScoWntX4     1 MSSRLFHNP---------------------------------------------------
LcoWntB      1 MAA---------------------------------------------------------
SciWntB      1 MAFVSYRTI---------------------------------------------------
SciWntO      1 ------------------------------------------------------------
HdujWntK     1 MKRLLFSSF---------------------------------------------------
HdujWntJ     1 MMSTEFISC------------------------------------------CKRS-----
LcoWntP      1 MCCINNSATPRGE------------IMH-------------------AKSSC--------
SciWntP      1 M-----------------------------------------------------------
ScoWntX2     1 MFKLR--------------------VM---------------------------------
LcoWntL      1 MSNVERILPCGPRESG------DECCFASV---------------KVSLARCSRSISPEE
SciWntL      1 MGQ---------------------------------------------------------
ScoWntX5     1 MTELA----------------------------------------------CTE------
LcoWntS      1 MA----------------------------------------------------------
SciWntS      1 MVCCLDERLSSLNLTRPLQADPWPTAMAPASPPAPEQAVPPNVEPSLLATTC--------
```

```
AquWntC      10 ------------------------------------------------------------
PfiWntC      12 ----------------------------------------------------LLTREEYG
HdujWntC      3 ------------------------------------------------------------
AquWntB       6 -----------------L-----------------------SLRMS----DV--------
PfiWntB       2 ------------------------------------------------------------
EmuWntB       8 ------------------------------------------------------------
SlaWntB       8 ------------------------------------------------------------
EfrWntB       8 ------------------------------------------------------------
AquWntA       6 ------------------------------------------------------------
EmuWntA       1 ------------------------------------------------------------
SlaWntA       8 ------------------------------------------------------------
EfrWntA       8 ------------------------------------------------------------
CreWntA       6 ------------------------------------------------------------
PsuWntA       3 ------------------------------------------------------------
CcaWntX1      2 ------------------------------------------------------------
HdujWntL     12 ------------------------------------------------------------
OloWntI       5 ------------------------------------------------------------
OcaWntI       4 ------------------------------------------------------------
CcaWntI       7 ------------------------------------------------------------
LcoWntA       4 ------------------------------------------------------------
SciWntA       5 ------------------------------------------------------------
ScoWnt-i      9 ------------------------------------------------------------
SciWntN       9 ------------------------------------------------------------
ScoWnt-ii     9 ------------------------------------------------------------
SciWntT      15 --------------------------------------------------------SALI
LcoWntE      10 ------------------------------------------------------------
SciWntE       5 ------------------------------------------------------------
ScoWnt-iii    5 ------------------------------------------------------------
LcoWntR       7 ------------------------------------------------------------
SciWntR       9 ------------------------------------------------------------
LcoWntC       5 ------------------------------------------------------------
SciWntC       2 ------------------------------------------------------------
ScoWnt-v      2 ------------------------------------------------------------
LcoWntI       9 ------------------------------------------------------------
SciWntI       5 ------------------------------------------------------------
ScoWnt-iv     3 ------------------------------------------------------------
LcoWntJ       6 ------------------------------------------------------------
SciWntJ       3 ------------------------------------------------------------
LcoWntG       7 ------------------------------------------------------------
SciWntG       6 ------------------------------------------------------------
ScoWnt-vi     6 ------------------------------------------------------------
OcaWnt2X     24 ISSVGIIPHQTY--------ETSNDHGT---------------------------NAKYI
EmuWntC       2 ------------------------------------------------------------
SlaWntC       1 ------------------------------------------------------------
EfrWntC      22 ---------------------------------------------------SAPVRSNSA
CreWntC      27 -------------------------------------------------------RSGAV
CcaWntX2     14 ----------------------TNSHDIT------------------KRSN---------
LcoWntD      12 ------------------------------------------------------------
SciWntD       2 ------------------------------------------------------------
ScoWntX1     11 ------------------------------------------------------------
LcoWntF       6 ------------------------------------------------------------
SciWntF       3 ------------------------------------------------------------
LcoWntQ      33 ---TNTIYGMMFALNIQQPKAEGNVHPQGKL-----GPSPC----------VMSLLTTRF
SciWntQ       1 ------------------------------------------------------------
HdujWntD     10 ------------------------------------------------------------
HdujWntE      1 ------------------------------------------------------------
HdujWntF     10 ------------------------------------------------------------
HdujWntH      7 ------------------------------------------------------------
HdujWntI     10 ------------------------------------------------------------
HdujWntG      6 ------------------------------------------------------------
OloWntII     10 -----------------------------------------------------------S
OcaWntIIa     3 ------------------------------------------------------------
OcaWntIIb     8 ------------------------------------------------------------
OcaWntIIc     6 ------------------------------------------------------------
OcaWnt5bX     2 ------------------------------------------------------------
OcaWnt6X     10 ----SLVPPL-----------------------------------------IAYKASARF
LcoWntK       4 ------------------------------------------------------------
SciWntK      21 ------------------------------------------------------------
LcoWntU       1 ------------------------------------------------------------
SciWntU       6 ------------------------------------------------------------
SciWntM      13 ------------------------------------------------------------
ScoWntX3      3 ------------------------------------------------------------
LcoWntH      10 ------------------------------------------------R---VPKGPGGA
SciWntH       4 ------------------------------------------------------------
ScoWntX4     10 -----------------------------------------------SRG-----GSSAL
LcoWntB       4 ------------------------------------------------------------
SciWntB      10 ------------------------------------------------------------
SciWntO       1 ------------------------------------------------------------
HdujWntK     10 -------------------------------------------------------LLSAS
HdujWntJ     14 -----------------------------------------------SRH--RIQVLPRR
LcoWntP      22 ----------------------------------------------------RPQKVLYS
SciWntP       2 ------------------------------------------------------------
ScoWntX2      8 ------------------------------------------------------------
LcoWntL      40 TQGTRAQPTSSLACRSTCSSSEANSSSP------------SQPAMSVRRG--AGWGHASH
SciWntL       4 ------------------------------------------------------------
ScoWntX5      9 ----RHIPLPSLAARPKGALNTSTSRTPRCMTRQPVAEVCAVPAASISRHKRCVFGMMQL
LcoWntS       3 ------------------------------------------------------------
SciWntS      53 ----------------------------------------------------SSCRSARY
```

```
AquWntC      10 --NYVSVLILFYCI----------------IISLNKT----------------------
PfiWntC      20 SLYFKHFKMYLVIL----------------LMVIFQAQAFSP------------------
HdujWntC      3 ------FLVLVAMAL--------------SLFALVVE-----------------------
AquWntB      14 ----GRALLLS-------------------------------------------------
PfiWntB       2 ------------------------------------------------------------
EmuWntB       8 ----DRLLYVSLMCI---------------------------------------------
SlaWntB       8 ----GRLLLLCLVCL---------------------------------------------
EfrWntB       8 ----DRLLLVGLIYI---------------------------------------------
AquWntA       6 --------LATAVC----------------LLMVFNGC----------------------
EmuWntA       1 ------------------------------------------------------------
SlaWntA       8 ----EHAVFLCLLVV--------------AVHLVISSS----------------------
EfrWntA       8 ----EHAVFLCFLVM--------------AVHLVISSSL---------------------
CreWntA       6 -SQWRGYICCAFLLM---------------LTVIINVQGNP-------------------
PsuWntA       3 ----LSVRIVFALV----------------LTVAATKSVFG-------------------
CcaWntX1      2 -------LFFASLVF-------------TFLACVTSV-----------------------
HdujWntL     12 ----YSLVLLKAVF----------------IALLQAPTFSPA------------------
OloWntI       5 ------------------------------IQNVVGNEY---------------------
OcaWntI       4 ----FRVVVAAACL----------------MAVVFSIEY---------------------
CcaWntI       7 ------------------------------------------------------------
LcoWntA       4 ----WQIILSLALC----------------LVLLAQRALS--------------------
SciWntA       5 --------YRLLSIV---------------MAAVLGLLES--------------------
ScoWnt-i      9 ----VKMLSVLAVI----------------LVLDLSFSTT--------------------
SciWntN       9 ----FSAVFLLS------------------VHRLSAQKHLAA------------------
ScoWnt-ii     9 ----GCALLISSLC----------------LVCESQLNQEAS------------------
SciWntT      19 QAS-TRILVVCFLM----------------LPLLQTGTVS--------------------
LcoWntE      10 ----TLVTLLAGIQ------------SRQSLVFGQVVQRQFVE-----------DH----
SciWntE       5 ----VAAFVLALIS------------DRYQVGQCQTAHHYRQLAVEVRPSVEQWDIG-EG
ScoWnt-iii    5 ----ALVLLISTIA------------TSRLLIVCAGQD-------IQVQPS-EEWDL----
LcoWntR       7 ---GRKLAMLLFIS----------------LVMGLASADSSSK----------------L
SciWntR       9 ----RSFLLLTTMVV-------------VALMPELSSSADVPE----------------T
LcoWntC       5 ----RREAVLVGVV----------------IIATFASIESR-------------------
SciWntC       2 ----DRLCVIYFAF----------------LSWMMSGTESR-------------------
ScoWnt-v      2 ----ARLCFGLAA-----------------LLSLTLTVECR-------------------
LcoWntI       9 ----LLQGILAMVS----------------LIWQLEVTNGH-------------------
SciWntI       5 ----KALWTCAVLV----------------SVHLIGTCFGH-------------------
ScoWnt-iv     3 ---GKIAWIVLLVV----------------AAHLICISTCH-------------------
LcoWntJ       6 ----SCFSFVCSCFQS--------------------------------------------
SciWntJ       3 --------ILIALA----------------LVAVQISTKVQL------------------
LcoWntG       7 ----MSCRCVMMMI----------------LLSCCGLS----------------------
SciWntG       6 ----FT--WIAAIL----------------VIACARRS----------------------
ScoWnt-vi     6 ----LNTLWSSAIL----------------LLTYVEMS----------------------
OcaWnt2X     49 VTVEKAMRAVVSLG----------------IVISISTLFSSE------------------
EmuWntC       2 --------------------------------LCFGLSVAQAP-----------------
SlaWntC       1 ------------------------------------------------------------
EfrWntC      31 SQNARTYILQVALT----------------LAFCIGISFAQGA-----------------
CreWntC      32 SSGCYPRWIWFAII----------------LCSCISSSLCDVT-----------------
CcaWntX2     25 --------------------------QFRPRIIRTDNIKQAGGQ-----RHCK--DGSCVM
LcoWntD      12 ----PAACLYVAIVV-------------ILLVQVPGDVSAST------------------
SciWntD       2 -------LLVATLVA-------------SCMVLHEGSTASDSG-----------DS----
ScoWntX1     11 ----VVLLSVLALT----------------LPCSFSQDL---------------------
LcoWntF       6 ----TPSLLLSLTAS---------------LIFLVATARAAAV----------------N
SciWntF       3 ----LPLPLLATFAI---------------IILAVSTVIGGQALL---------DS---N
LcoWntQ      75 SCPNFNYLVTLATF----------------LLACLNATALGD------------------
SciWntQ       1 ------------------------------------------------------------
HdujWntD     10 ------------------------------TFLLMSR-----------------------
HdujWntE      1 ----MSAAMLAVLC---------------AFFPVITA-----------------------
HdujWntF     10 ----CRHILMVCSY----------------LFVCFTANSCSNM-----------------
HdujWntH      7 ----VRATNIVLLA----------------FLSSFVAERSALQ-----PHC---------
HdujWntI     10 ----LPMVPVLALA----------------LVVLAGPDRAVAA-----------------
HdujWntG      6 -TTKTALLVLTVLLL------------GNSLLQQSEA-----------------------
OloWntII     11 PLYLDLKMKQTCIVL---------------IILFCIAV----------------------
OcaWntIIa     3 ------RLSLPSLFL---------------CLVFASA-----------------------
OcaWntIIb     8 ----LMYRLLSWLFS---------------SLVCTSI-----------------------
OcaWntIIc     6 ------------------------------------------------------------
OcaWnt5bX     2 ----FSVFCLCLVF----------------GVTAFPLAAASP------------------
OcaWnt6X     25 IRASVSTLCVSRLLTQQHNTTMPQLFTCLVLAICLSCVRAAC------------------
LcoWntK       4 ----MPMPMWCALFV--------------SLLIRMHT-----------------------
SciWntK      21 ----ASSVALAALLQ-------------LQLFFVATVTAAPPG-----------------
LcoWntU       1 ------------------------------------------------------------
SciWntU       6 -----------------------------TFVVANGTSDAYPT-----------------
SciWntM      13 ----GGCVLTVTLP----------------LLILASVCSRSAV-----------------
ScoWntX3      3 ----------MLLV----------------IVILLSTGLCRKT-----------------
LcoWntH      19 VS--SKAPMTLSLI----------------VLLCLST-----------------------
SciWntH       4 ----SMISLLAPLL----------------LLLTLSA-----------------------
ScoWntX4     18 QVRRAPIGISVVLL----------------LLLALSA-----------------------
LcoWntB       4 ----RMLFLVVAVYLM--------------LQQAVKGDRQEYT-----------------
SciWntB      10 ----SSKFVITIIY----------------LLMAITTIQ---------------------
SciWntO       1 ------------------------------------------------------------
HdujWntK     15 SRKALRLVCLSLLVL-------------FGLLCETSSN----------------------
HdujWntJ     25 GTMWPSLFTLVVLCN-------------LQLVACSLSNY---------------------
LcoWntP      30 GRTSLLAVLVVLML----------------VQAAIGTGEASVK-----------------
SciWntP       2 ASFVRPLALITLLA----------------LCATVCTKR-STD-----------------
ScoWntX2      8 ASVVRPLPLILTLV----------------LSVCVATAELPPN-----------------
LcoWntL      86 SMRQPVLPILFAFL----------------LCISLGSCRLQII-----------------
SciWntL       4 ----RLFLLAASVL----------------VLCDLS------------------------
ScoWntX5     65 SLTSTLLVFLALLV----------------LSADLTA-----------------------
LcoWntS       3 GRTLLLKCPLAALA----------------IIILLNAPYSEGG-----------------
SciWntS      61 GSTSLRLLRYLLVP----------------LLVLLVQQRAHAA-----------------
```

```
AquWntC      29 -----------------------------GF----GLANSSNT-----------------
PfiWntC      46 -DSVVET----------------------------ETTNTTDP-----------------
HdujWntC     20 ------------------------------------------------------------
AquWntB      21 ------------------------------WA---FISFTMDE---VT------------
PfiWntB       2 ------------------------------------------------------------
EmuWntB      19 ------------------------------W---------------VFH-----------
SlaWntB      19 ------------------------------W---------------VFH-----------
EfrWntB      19 ------------------------------W---------------MFH-----------
AquWntA      20 --------------------------LASWW----SLGVYNDL-----------------
EmuWntA       1 ------------------------------------------------------------
SlaWntA      28 -------------------------SSTSWW----SLSVSSLS-----------------
EfrWntA      29 -------------------------SSPSWW----SLSVSSLS-----------------
CreWntA      31 --------------------------STSWW----SVGITDCGK-RVT------------
PsuWntA      24 --------------------------GIGWW----SLGVSECS-----------------
CcaWntX1     19 --------------------------YGSWW----EMARDVAD-----------------
HdujWntL     34 --------------------------TATYW----ALNLATIA-----------------
OloWntI      14 -----------------------------------ELPFFLSL-----------------
OcaWntI      23 -----------------------------------KLPFFISC-----------------
CcaWntI       7 ------------------------------------------------------------
LcoWntA      24 --------------------------TTTGY----SFPYHTAA---AL------------
SciWntA      22 --------------------------GLSNA----EFPYHTSA---AL------------
ScoWnt-i     29 --------------------------MSEGT----LFPYHTSA---AL------------
SciWntN      29 -----------------------TTLLDPDL----RLPYHIAT---AM------------
ScoWnt-ii    31 -----------------------TSLLNSDL----SFPYHIAT---AL------------
SciWntT      42 --------------------------QSLFF----SLPFHTAS---GLASRSQHASRILS
LcoWntE      39 ----VGTAPS-------------------------VTPTAGNV-----------------
SciWntE      48 ESSTTGNATT---------------TDSTSS----SVPSAVGGHDPAE------------
ScoWnt-iii   38 ----QGNAST------------------------------------SA------------
LcoWntR      32 ------TLPQ------------SGLDGLSSW----SVPFQTTV---ALKSINNDH-----
SciWntR      36 TEETIGRISQ------------TG--FLSQW----RVPFQAKV---ALSEINDPP-----
LcoWntC      26 -----------------------------------LLPSTVKS---IIGAL---------
SciWntC      23 -----------------------------------LLPSAIDR---IMGSL---------
ScoWnt-v     22 -----------------------------------LLPSTINT---IMGSLN--------
LcoWntI      30 -----------------------------------FLPSAIEM---VN------------
SciWntI      26 -----------------------------------FLPSAIEA---VT------------
ScoWnt-iv    25 -----------------------------------FLPSAIEL---VK------------
LcoWntJ      18 -----------------------------------RLGFHSMA---AL-----------Q
SciWntJ      21 --------------------------TSGSS----SLSFHSMA---AI-----------Q
LcoWntG      25 --------------------------HQSVW----RTGISISSK----------------
SciWntG      22 --------------------------EQSVW----MTGISISSK----------------
ScoWnt-vi    24 --------------------------HQTVW----RTGISISSK----------------
OcaWnt2X     75 -----------------------------------ALQIKGSS-----------------
EmuWntC      13 ----------------------DGTSGGN------TFSSSIPG-----------------
SlaWntC       1 ------------------------------------------------------------
EfrWntC      58 ----------------------DTTGGTTTA----STPTSTAS-----------------
CreWntC      59 TSNSAGTTSAP------------------------AMTTPITTT-PMT------------
CcaWntX2     53 NDQGSGNQNGFDNDDE------DEEGSADSWLPLEMLSWSVKEV-RVPSN----------
LcoWntD      37 -------------------------NLESWW----TMANKIQL-----------------
SciWntD      27 -------------------PAQNGGDLETWW----TMANRVIL-----------------
ScoWntX1     30 ------------------------RSLETWW----TMANQVRL-----------------
LcoWntF      31 PEGTVLSSSEYEEGLPPLRGV-PSSRRQDWW----SLSRLIDV-----------------
SciWntF      32 SDDRALSTSQPPPALP---GIAAYRGKLQWW----SLSRLIND-----------------
LcoWntQ     101 ------TSMGVEEDSC------DLYQATKWW----SMARLINR-----------------
SciWntQ       1 ------------------------------------------------------------
HdujWntD     17 -----------------------------------SLALQSGLV----------------
HdujWntE     19 ---------------------------QGFW----SLALEELT-----------------
HdujWntF     33 ---------------CS--NDLHEV----------LLSVNPKL---VS------------
HdujWntH     33 -------------DSC---NEFGFV----------KLSVDPDL---VD------------
HdujWntI     33 -------------------------GNTRYW----ALYHEDVQ-----------------
HdujWntG     30 -------------------------ALSTWW----TMANIPNA-----------------
OloWntII     33 --------------------------QATWL----DLGQRLID-----------------
OcaWntIIa    19 --------------------------TGTWL----ELGQKLIE-----------------
OcaWntIIb    26 --------------------------SATWL----ELGQKLDE-----------------
OcaWntIIc     6 -----------------------------------ELGQKLDE-----------------
OcaWnt5bX    24 -------------------------KPPAWW----QFGEYLDK-----------------
OcaWnt6X     67 -------------------------APPAWW----QFGEYMKD-----------------
LcoWntK      23 ------------------------------------------------------------
SciWntK      47 ----------------------------QWW---------------MYG-----------
LcoWntU       1 ------------------------------------------------------------
SciWntU      20 ------------------------------------------------------------
SciWntM      36 --------------------------ASIFWKQ-------------IYE-----------
ScoWntX3     20 -------------------------HAAAWWQE-------------AFR-----------
LcoWntH      38 ----------------------CRTANAIWWEE-D-------------------------
SciWntH      21 ----------------------CNTANGIWWDT-A-------------------------
ScoWntX4     39 ---------------------WCRTTNAIWWNP-E-------------------------
LcoWntB      29 -----------------------------------PLSRNI-------------------
SciWntB      29 -----------------------------------ALPGRRRP-----------------
SciWntO       1 ------------------------------------------------------------
HdujWntK     40 -----------------------------------RLPRTSNP-----------------
HdujWntJ     51 -----------------------------------SLKQSVPA-----------------
LcoWntP      57 -----------------------PRNHHKTF----DLWATLKD-----------------
SciWntP      28 -----------------------GRLGQEPF----DLSRTVQR-----------------
ScoWntX2     35 -----------------------GGIKAERF----DLARTVQK-----------------
LcoWntL     113 ------------------------------------------------------------
SciWntL      20 ------------------------------W-----------------------------
ScoWntX5     86 ------------------------------------------------------------
LcoWntS      30 -----------------------KRYSYYWW----DISLLRSEG----------------
SciWntS      88 -----------------------KTYSFYWW----DISLLRSEG----------------
```

```
                                                    *
AquWntC      39 ---ATP---------------------VYLN-NQTFTCHNLT----------NAN-QRIM
PfiWntC      60 ---SSR----------------------SLS-APLFTCRDLR----------TRS-QRDL
HdujWntC     20 -----------------------------NG-AAIAKCQDVY--------DLTYS-QRQF
AquWntB      33 -----------------------------PF-PNHIICLYIP--------GLNDV-QRDL
PfiWntB       2 ------------------------------------------------------------
EmuWntB      23 ---AAQ-----------------------TF-SPDLICLTIP--------SLNAQ-QKAL
SlaWntB      23 ---IAQ-----------------------PF-SPDLICLTIP--------SLNAQ-QKAL
EfrWntB      23 ---VAQ-----------------------PF-SPDFICLTIP--------NLNAQ-QKAL
AquWntA      33 ---------------------------SERV-VDSTPCESLT-------NILNSS-QQTF
EmuWntA       1 ------------------------------------------------------------
SlaWntA      42 ---NPS-----------------------AD-TSTVDCSNST-------YLASPF-AQAT
EfrWntA      43 ---TPS-----------------------AN-PNSIDCNNST-------FLASPF-AQEV
CreWntA      48 ----------------------------TAN-STCPNCAKLQ-----QDQWFSAE-QASK
PsuWntA      37 ---------------------------LGSK-APCETCAKLT-----KNYNLTRD-QKEA
CcaWntX1     32 -----------------------------RD-MDRLHCDRLE-----YDYGLTKE-QVDM
HdujWntL     47 ----------------------------ATE-GESGVCNR---------SALTQS-QYLQ
OloWntI      22 ---GSK-----------------------QL-KQRVDCVS----------PMTDA-QREW
OcaWntI      31 ---AVD-----------------------DL-KGKELCPR----------TLPDE-QFRL
CcaWntI       7 --------------------------------------------------DVSLR-----
LcoWntA      39 ---STV-ET--------------------GP-IRKSTCKRLP--------GLTDK-QVKW
SciWntA      37 ---ATS-GL--------------------RP-IRRGDCKRLT--------GLTEG-QFQW
ScoWnt-i     44 ---STS-GL--------------------AP-VRKSSCKRLE--------GLTVA-QVKW
SciWntN      47 ---SSA-EL--------------------EP-IRKGNCKRLY--------GLSTS-QVDW
ScoWnt-ii    49 ---SAS-EA--------------------EP-IRKGSCKRLF--------GLTQR-QVEW
SciWntT      69 LSMSSS-IK--------------------KD-ITKETCRQVV--------GLTSK-QRRW
LcoWntE      53 ---NAG--------------------TPEES-DRRAACRFLP--------GLTQK-QRLW
SciWntE      77 ---ETD-GI--------------------EP-DGRAACSFLP--------GLTER-QRLW
ScoWnt-iii   46 ---EHG-GG--------------------EP-DSRAACHFLP--------GLTER-QRLW
LcoWntR      62 ---SDDFDLSQ-------------LAAPVNE-TDRALLKTV---------GLTRREQFTW
SciWntR      70 ---GQDFSLHD-------------LVRTAND-TDDAQLNAL---------GLSRE-QQAW
LcoWntC      39 ---GSE-----------------------AI-TRNAFCNRLQ--------GLSDA-ESKW
SciWntC      36 ---ETK-----------------------AI-TQTRFCDKLN--------GLTQL-QKEW
ScoWnt-v     36 ---DSR-----------------------VI-SHQRFCNKLN--------GLTAA-QATW
LcoWntI      40 ---KQR-YL-------------------NAA-VEKRLCRNMP--------GLTDR-QRSW
SciWntI      36 ---RQR-FL-------------------DET-VQRRLCRNMP--------GLTDR-QRSW
ScoWnt-iv    35 ---KQR-YL-------------------DET-VQRRLCRNMP--------GLSDQ-QRSW
LcoWntJ      29 ASQSMH-VL--------------------DG-LGPAQCQHLP--------GLTES-QRLW
SciWntJ      37 IVQSMR-AL--------------------KT-LGPEQCEHLP--------GLSDH-QKAW
LcoWntG      39 ---TLE-RL--------------------QG-VVTKTCNSIP--------GLTIR-QRAF
SciWntG      36 ---TLD-PL--------------------EG-ALQKTCSSIA--------GLTHR-QRMF
ScoWnt-vi    38 ---SLD-PL--------------------QG-VIEKTCGSVS--------GLTQR-QRMF
OcaWnt2X     83 ---SEE-GL--------------RQTKGGGI-RPADICKFIP--------GLSRS-QVRF
EmuWntC      28 ---SPT----------------------PVQ-INMLECLSAF--------DIQAQ-AVLV
SlaWntC       1 ------------------------------------------------------------
EfrWntC      75 ---SAT----------------------PVQ-INMMECQSAF--------DIQAQ-TVLV
CreWntC      82 ---STSTSL-----------------PTPQR-FNIADCVTSF--------DSSDQ-RRYI
CcaWntX2     96 ---KTKSSLIG-DRNTTVVHLVELDATKQHY-RTVVKCRDVTQLV-----------NSDL
LcoWntD      51 ---PQG-----------------------KF-HVFPGCDKLN--------LMTRN-QKLS
SciWntD      47 ---PQG-----------------------NM-GTFPGCEHLN--------LMTDN-QRRA
ScoWntX1     45 ---PAG----------------------TLGPVFFPGCDQLK--------LLTEN-QRAA
LcoWntF      69 ---NTR-QL-------------------ASA-SSVEQCNSLP---------LSRR-QREL
SciWntF      68 ---RTG-RL-------------------KSA-SSVRACQRLP---------LSKR-QKQL
LcoWntQ     128 ---TSL-----------------------EL-KTFSSCDDLP---------LTDR-QRGE
SciWntQ       1 ---------------------------------------------------MSAG-QASF
HdujWntD     26 ---QPN-----------------------GH-LAPANCSEYL-----IKGVIQPH-QYEV
HdujWntE     31 ---AAD-----------------------GQ-FVRPNCVSYH-----SSGLLTQE-QYQL
HdujWntF     51 ---RSG-SL--------------------VE-RGFKNCFEFN-------GVLTQN-QLNM
HdujWntH     52 ---ANG----------------------IVV-NKLADCMEYD-------EVLSPQ-QMSV
HdujWntI     47 -----------------------------VP-LSHQRCSELQ-----LAGVLAPS-QHLL
HdujWntG     44 -------DL---------------SKPDGSS-LIPLDCKAHQ-------ASMSAA-QFQL
OloWntII     46 ---EEI-RLRGPDFDPDIV-----HIPEEDI-KPTLDCYDVN---------MTTG-QFQI
OcaWntIIa    32 ---EEI-KNRPPGYDPTIV-----HTKENDV-KVTLDCSSVN---------MTSA-QQKM
OcaWntIIb    39 ---ERQ-KLIPPGQDPTII-----RSKENQI-KVALGCSIVN---------MTLP-QHQI
OcaWntIIc    14 ---ERQ-KLIPPGQDPTII-----RSKENQI-KVALGCSIVN---------MTLP-QHQI
OcaWnt5bX    38 ---QLG-KY--------------------QV-LTTIPCSYFN---------LSPR-QREM
OcaWnt6X     81 ---QAS-KY--------------------GI-WTSIPCEKFN---------LPQT-QHDI
LcoWntK      23 ---SSE----------------------SVL-AVKDLCNDTD-----SSQQEDGA-SRVI
SciWntK      53 ---SSQ----------------------SVR-TLREQCSEWN----SSSLPDDIA-SQLI
LcoWntU       1 ------------------------------------------------------------
SciWntU      20 ----------------------------RFS-HFPFRCKVRE--------GAPLE-SHSQ
SciWntM      46 ---GSS-----------------------QA-WQNGRCDPEL---------EVSQ-QSQH
ScoWntX3     31 ---GAG-----------------------QA-WQNGKCDENL---------DVSL-QSPQ
LcoWntH      50 ---AED-NL--------------------FN-LHQRHCAEDL-------AANRTS-PLLI
SciWntH      33 ---ASD-SL--------------------HK-QHAKHCPE---------SAKLNS-SLQL
ScoWntX4     52 ---AAD-SL--------------------HR-LHYKHCPE---------GSKHNS-SLHL
LcoWntB      35 --------------------------------TNWELCATRE----------TAE-GREL
SciWntB      37 ---HNN----------------------VNM-TFMDICSKMD----------TVT-AQVR
SciWntO       1 ------------------------------------------------------------
HdujWntK     48 ----------------------------------YKTCYQYR------NYRPTSY-QLRY
HdujWntJ     59 --------------------------------SLGTNCSAWI-----QRYNLTAQ-QSHL
LcoWntP      73 ---ISG----------------------------------------RRFADQFPH-EVSV
SciWntP      44 ---AVR----------------------------------------RPYFRQRQY-EKSV
ScoWntX2     51 ---IAG----------------------------------------RRYFDTHEN-DKKI
LcoWntL     113 --------------------------------QPSVDCPRNM-SAEDMFQFLTEG-QKRV
SciWntL      21 ----------------------------AVF-VTRSTCDRTDQPIHDMVEALNSE-QRKI
ScoWntX5     86 ----------------------------ASL-IASRRCDSVTRTIDDMVGALNER-QRRF
LcoWntS      47 ----------------------------------------------VTGLDYTGI-QKVL
SciWntS     105 ----------------------------------------------HHHADYTGL-QKQL
```

```
                *                                    *           *
AquWntC      63 CFT-----TP----GLLK-AIVDAEQ--------LARKECSNQLEYE--RWNCSGFA---
PfiWntC      83 CYD-----TP----GLLE-ILIKSEQ--------LAKEECEFWFKDH--QWKCFGFS---
HdujWntC     41 CRV-----NR----ELLP-IIEKAEA--------SAMRECQFLFKSN--RWNCSGFG---
AquWntB      54 CIR-----YP----KLVPIIIQEVPP--------LFYSECREQFKYE--RWNCSETI---
PfiWntB       2 --E-----YP----AAMA-IIQEIAD--------AIHDECIDQFKND--RWNCTEVI---
EmuWntB      47 CRQ-----LP----KAMN-VLVNATL--------AYTDECNWQFRKD--RWNCSVGG---
SlaWntB      47 CRQ-----LP----KAMN-VLVNATL--------EYADECNWQFRRD--RWNCTSGG---
EfrWntB      47 CRQ-----LP----KAMN-VLVNVTL--------AYADECNWQFRRD--RWNCTSGG---
AquWntA      57 CNY-----NR----KIVN-SIAIGTR--------RGIVACQQNFANW--RWNCTTFT---
EmuWntA       1 ------------------------------------------------------------
SlaWntA      67 CAS-----DK----KIVL-AIARGTK--------AAIVQCQMEFANS--RWNCTTFL---
EfrWntA      68 CAK-----DK----NIIL-AVSRGAK--------AAIIQCQSEFGNL--RWNCTTFL---
CreWntA      73 CRS-----EP----AIRD-SLSNGAR--------AAIIDCQRRFNES--RWNCSTLF---
PsuWntA      63 CIQ-----DP----SQVQ-AIARGTR--------KAIIDCQAVFSER--KWNCSTFS---
CcaWntX1     56 CKSFR---YY----PLMS-DVFFAAE--------MALKQCKRQFKHN--KWNCWTAS---
HdujWntL     68 CLS-----NR----DTVY-STALGVE--------LGIAQCRRVFVNN--RWNCSATG---
OloWntI      44 CRW-----ND----DIVG-LIVDGAT--------KGLDECEYRFQKR--RWNCTSSE---
OcaWntI      53 CRW-----ND----VIMA-KFVDGAR--------IGLRECEYQFRNR--RWNCTAAK---
CcaWntI      12 ------------LFGIVS------------------------DFSTN-------------
LcoWntA      65 CKS-----HY----VFLQ-PIVSGTK--------LGLDECKRRFGSR--RWSCPTDL---
SciWntA      63 CKT-----HS----VFMQ-PIVSGAK--------LGLAECQRRFSNR--RWNCPTDQ---
ScoWnt-i     70 CKS-----HH----VFMQ-PIASGAK--------LGLAECKRRFSDR--RWSCPTDR---
SciWntN      73 CKK-----NS----VFFR-PIVIGTR--------LGLNECKRRFARR--RWSCPVDQ---
ScoWnt-ii    75 CKK-----HY----DFLQ-PIVIGTR--------LGLDECKRRFALR--LWSCPIDR---
SciWntT      98 CRK-----NY----ELLA-AIGDGAR--------LGLAECQRRFEKR--RWSCPIAQ---
LcoWntE      80 CLD-----NY----IFLE-PIASGAQ--------IAQNQCRKQFSDR--RWNCPTNR---
SciWntE     103 CRD-----NY----VFLE-PIASGAQ--------IALAQCRKQFADR--RWNCPTDN---
ScoWnt-iii   72 CLD-----NY----VFLE-PIATGAQ--------LALGQCRKQFADR--RWSCPTHN---
LcoWntR      96 AQR-----YN----KLLQ-PMSEGAI--------LALRECHRRFKDE--RWNCPIHK---
SciWntR     103 AKN-----NT----LLVG-PVADGAV--------LGLRECHRRFKYE--RWNCPIHR---
LcoWntC      63 CRK-----YT----PFLP-AIGMGAD--------LALKECQKRFQYD--RWNCQLDD---
SciWntC      60 CHK-----YT----PFVP-AIGMGAN--------LALKECRKAFQYE--RWNCTMDD---
ScoWnt-v     60 CHK-----YT----AFLP-AIGLGAN--------LAMKECRKAFQYE--RWNCSMDD---
LcoWntI      67 CRS-----HL----PFVG-PIAQGAT--------LGLSECARQFEYE--RWNCPLNY---
SciWntI      63 CRT-----HL----PFVG-PIAQGAR--------LGLRECRRQFMFE--RWNCPIND---
ScoWnt-iv    62 CRA-----HL----PFVR-PIAQGAN--------LGLSECRRQFMFE--RWNCPIND---
LcoWntJ      58 CRH-----HH----VFMQ-PIARGTL--------LGLDECSRQFEFQ--RWNCPVND---
SciWntJ      66 CRQ-----HH----VFMQ-PIAQGTR--------LGLEECSRQFHYQ--RWNCPMNN---
LcoWntG      65 CMN-----HS----DLMD-PIDQGAK--------LGREECSRRMSGR--RWNCPKNN---
SciWntG      62 CLT-----RS----ELMD-PIDQGAN--------LGRLECSRRMSKK--RWNCPSDD---
ScoWnt-vi    64 CLA-----RS----QLMD-PINQGAN--------LGQEECHRRMSGR--RWNCPKNN---
OcaWnt2X    115 CRR-----RP----WLMT-SLGNGFK--------AARNECRELFARADVRWNCSTSN---
EmuWntC      53 CNN-----YP----DLYA-VLKFAEQ--------VGRDECQKAFQGS--KWNCSTFS---
SlaWntC       1 ------------------------------------------------------------
EfrWntC     100 CNN-----YP----DLYP-VLKFAEQ--------VGRDECQKTFQGS--KWNCSSFS---
CreWntC     112 CLN-----HK----ELFP-LLKFAEQ--------VGKSTCEEDFEHE--HWNCSSFSL--
CcaWntX2    140 CER-----YP----TVLP-AIIQGVQ--------LAVEETIHLLKDR--RWDGYALK---
LcoWntD      75 CDN-----GSAQGVNRMT-VLGRAAL--------ATKIECQKQFASK--RWNCSSFE---
SciWntD      71 CDG-----GSDLGVRRMT-ILARASL--------ATTLECQKQFAAQ--RWNCPKFA---
ScoWntX1     70 CNQ-----GSEIGIARMT-VLARASL--------ATTMECEKQFTGQ--RWNCETFL---
LcoWntF      95 CNSTRVGEGS----ALMT-VAARASL--------AAVRECQRQFADN--RWRCQHFE---
SciWntF      94 CTSRRSGYGP----ALVT-IAARASL--------AAVRECQRQFQDR--LWTCEHFE---
LcoWntQ     151 CRQ-----DASQGYGRMR-VMARASL--------ATARECSRQFQDR--RWNCSSFQ---
SciWntQ       9 CSD-----DARLGFERTR-VMARASL--------ATTRECARQFADR--RWNCPSFL---
HdujWntD     53 CMN-----NS----YLVS-AITKGLR--------RAIASCQSTFKNN--IWNCTAYK---
HdujWntE     58 CQE-----DG----LVLA-AVSKGLR--------RAISDCQSNFAHQ--RWNCSAFE---
HdujWntF     78 CTR-----DP----DYLS-CFALGMR--------RAVQWCGRVFNQS--KWNCSHAV---
HdujWntH     78 CLG----KGS----RYLS-CFALGMR--------KAVNWCGRVFNQS--KWNCSHAV---
HdujWntI     71 CVN-----RP----QVLR-AVVRGLQ--------RAVHYCQHTFRDS--PWDCPLDA---
HdujWntG     73 CSD-----NP----HLVT-AVVLGIK--------RAIHFCQQQFKGQ--CWDCASFV---
OloWntII     86 CEK----SEK----GLLV-AIAQGIN--------AAVYTCKRDFENR--QWNCTDP----
OcaWntIIa    72 CQS----AEK----GLIV-AIAQGIN--------AAVYSCRRNMANH--SWNCPVPG---
OcaWntIIb    79 CVN----AEK----GFPA-AVAKGIN--------AATYVCRRDMKDF--PWNCPIPG---
OcaWntIIc    54 CVN----AEK----GFPA-AVAKGIN--------AATYVCRRDMKDF--PWNCPIPG---
OcaWnt5bX    63 CENP---RNR----DLMI-KLGLAVN--------LAVQVSNETFANR--RWRSHYTG---
OcaWnt6X    106 CANP---PNR----ELII-RLAVAVN--------VAEQACTQTFAGR--RWRCHYTG---
LcoWntK      51 CPL-----AN----DTVW-QISRSSY--------EAVENCQRLFRNH--RWNCSTSS---
SciWntK      82 CPL-----SE----RTVE-EIEKSAY--------QAVWQCRVAFAKH--RWNCPNTT---
LcoWntU       1 --------------RLWT-TLQRGLD--------QGIEECQKVMKTG-TAWTCPVTR---
SciWntU      42 CLR----RDE----PLWT-TIQRGRT--------EGIAQCKKVMKDN--TWSCAVEQ---
SciWntM      69 CIL-----SH----PLLKPVLRESLR--------IGIDLCKLRFRGK--RFNCSINP---
ScoWntX3     54 CIL-----SH----PLLRPVLQYSLQ--------EAVIQCQLKFHDQ--RFNCSVNA---
LcoWntH      77 CRL-----SG----DEMT-SLIHGAR--------EGLRICQFQLNNE--RWDCTSSD---
SciWntH      58 CRL-----SG----EEIT-SLMKGAA--------GGIFDCQMLMASQ--RWNCSSSD---
ScoWntX4     77 CKL-----SG----GEIS-SLMRGAA--------KGIYECQMQASTQ--RWNCSSSN---
LcoWntB      52 C------------------FARNGVV--------KAMALCKNQFKNE--RWNCPESS---
SciWntB      60 C------------------FARRGAV--------LAVNTCQDAFFQE--KWNCPESS---
SciWntO       1 ----------------------------------MAVQECRTRFAQR--QWNCVVKP---
HdujWntK     67 CTN-----DH----ALFENVASVGVKLKPSSPTPLFEEECQYWMAVE--RWNCSGIK---
HdujWntJ     81 CRT-----NH----SLFESVATIGISSDVEAPS-LFERECQYWMEDE--RWNCSGIK---
LcoWntP      89 VRRYT--SSE----SWKA-VFRNASK--------FTIEQCRCAMGKS--KWNGTTSS---
SciWntP      60 LEHHM---TE----SWLE-ILKEASE--------WLSSHCACQFGRN--MWNCTTSS---
ScoWntX2     67 LLHHM---SE----QWLA-ILRESSE--------WVTGHCMCQFAKS--RWNGTSTS---
LcoWntL     139 LQD-----SR----NSTL-LFSDGES--------CALQACKKWTKGT--RWGCHMNE---
SciWntL      51 LAQ-----HA-IN-ATTP-LMYRGEY--------CAVHVCQELMQGT--RWACHLNH---
ScoWntX5    116 LLV-----NANLT-SCTP-IISRGER--------CAVSLCQEIMQGT--RWGCYLED---
LcoWntS      60 IDA-----GA----VNTT-LLDRARV--------LFLSNCLTRMTNR--RWNCSRNMPTQ
SciWntS     118 VDG-----GA----VNRS-LLHTARE--------YFLKECVIRMHPM--QWNCSRTTQRQ
```

```
                                                                    *            *
AquWntC      100 ----------VITPSN---VTKYATAETAAIHSLMSAALAHVVTRDCRFNG--M----QC
PfiWntC      120 ----------MLTPSN---VTKRASKESSFIYAIISATLTHTITGACKDEI--I----DC
HdujWntC      78 ----------VLRSSY---TKEVATAETAFQLALMSAVLAKNIAQACKEGS-----TSLC
AquWntB       92 --------PPI-AGDLS-KDLKRLSKETAFTYALTSAIMVRVITKACSDGR-----LQNC
PfiWntB       37 --------PPI-IGDPY-SDLKRSTKESAFMHALTSAATVHVITKACSDGR-----IINC
EmuWntB       84 --------IPI-FASK---IAFNRSREAAFTYALVSAITAHSITSACANSL--L--GSAC
SlaWntB       84 --------IPV-FASK---LAFNKSRETAFTYALTSAVTVHAITTACSNNI--L--GAAC
EfrWntB       84 --------IPV-FASR---VAYNKSRETAFTYALTSAITAHSITTACANGL--L--GSAC
AquWntA       94 --------GENLFGA----FVKNNTRETAVINALLTAGAERQIALDCRDEK-----LPNC
EmuWntA        1 ---------------------------------------------RCRS--------LNC
SlaWntA      104 --------GQYLFGK----FLTLGTAESAAVYSFMSAGAAHELAQACRTGA-----VSNC
EfrWntA      105 --------GQHLFGK----FISTGTIESAAVYSFMSAGAAHELAGACRTGA-----VVNC
CreWntA      110 --------GNHLFGSF---VATGKTRETGVLNAYFAAGAVSAIAEDCHNQR-----IASC
PsuWntA      100 --------GENLFGRF---VTESRTRETAVLFAFLSAGAIQEVAEACHEQR-----LLNC
CcaWntX1      95 --------TGPLFGGA---LKNGTTREAAFVQALFTAVLSAKVTKKCSNPR--FHEPLTC
HdujWntL     105 -------PHERSFAD----ATSRGTPETAFVQAILSAGMMSLVSRYCKEGR-----LLRC
OloWntI       81 --------RDH-FRA----ALSRGTRESAFTYAVTSAAIAWSVSRQCALRE--D--LTQC
OcaWntI       90 --------ADH-FEK----VMAKGSREAAFTYAVASAAITWSISRECALSD-----LPEC
CcaWntI       23 --------VVHVLKLI---VLLSATRETAFVFAMTVAGIIYSVSRECALGD-----LREC
LcoWntA      102 --------PDI-FAK----ALTTANPEAAFVHGIESAGITLAIARTCSRGD--A--LSHC
SciWntA      100 --------PEV-FAK----AIETANPEASFVHSIQSAGITLAIARTCSRGD--A--LKFC
ScoWnt-i     107 --------QSV-FRK----ALITANPEAAFVHGIQSAGITLAIARTCSRGD--A--LSHC
SciWntN      110 --------PNI-FSK----VLNTATPEAAFVHGIESAGITLAIARTCSRGD--A--LSHC
ScoWnt-ii    112 --------PNV-FRR----VLDTANPEAAFVRGIESAGITLAIARTCSRGN--T--VKHC
SciWntT      135 --------PSI-FSK----ILEAASKETAYVHSIQSAGITLAIARSCSRGE--A--STLC
LcoWntE      117 --------PAV-FSK----IYDRASRETAFVHAIQSAGVTLTVARTCSRGQ--A--PGWC
SciWntE      140 --------PAV-FSK----IYDKGSQETAFVHSIQSAGATLAIARTCSRGH--A--PRWC
ScoWnt-iii   109 --------PAV-FGK----IYDRGSRETAFVHAIQSAGATLAIARTCSRGH--T--PRWC
LcoWntR      133 --------PAS-FVK----IFKIGGPEAALVYAMQAAGMSLAVARTCSQGD--T--RGAC
SciWntR      140 --------SET-FQK----VFMIGGREAAFLYAIQAAGISLAVARTCSKGK--T--GGSC
LcoWntC      100 --------ER--YRH----LASFESRETAFVDSIVSAGVMMAISRTCSKGN--L--TKYC
SciWntC       97 --------SS--FQH----VSSFQSKETAFVDAIVSAGVMMAISRTCSRGK--L--TKYC
ScoWnt-v      97 --------AS--FTH----VSSFESKETAFVDAIVSAGVMMAISRMCTKGQ--L--VRYC
LcoWntI      104 --------STANFNF----IMSTGSREAAFVSSIQAAGITLAIARTCSSGNEST--ADFC
SciWntI      100 --------STTNYHF----IMSTGSREASFVSSIQAAGITLAIARTCSAGN--V--TRFC
ScoWnt-iv     99 --------STANFQY----IMATASREAAFVASIQAAGVTLAIARTCSGGN--V--TRFC
LcoWntJ       95 --------SRA-IET----ALNLRTAETAFLQSIQSAGIMQAIARTCSRGN--I--TKWC
SciWntJ      103 --------SMA-IQN----ALRLDTAEAAFLQSIQSAGMMQAVARVCSRGN--V--TEWC
LcoWntG      102 --------TDV-LKA----ALVKGYKESAFLQAIQSSGVVQGIARICSAGK--A--NKHC
SciWntG       99 --------SVL-LKA----ALVKGYRESAFLQAIQSSGVVQAVARVCSSGK--A--EKHC
ScoWnt-vi    101 --------TVL-LKA----ALVKGYIESAFLQAIQSSGVVQALARVCSSGK--V--SKHC
OcaWnt2X     154 --------GAFVFSP----ILRSSSKEAAFAWAISAAGVVCAVARDCKMGR-----LTNC
EmuWntC       90 ----------ILKSSN---IVKKDIIETAYIRALQVAVIAHTVAKACGTQT-----LVSC
SlaWntC        1 -----------------------------YIRALQSAVIAHTVAKACRTGT-----LVSC
EfrWntC      137 ----------ILKPPS---IVRKDIVETAYIRALQVAVIAHTVAKACRTRT-----LASC
CreWntC      150 ------------LKQPSITKGDYIYKESAYVYSLSMAVIAHTVAMGCVEE------IFNC
CcaWntX2     177 --------KKQ-VGEN---LLRQATKESAFTHAIISAGITHVVTKSCSKHL-----IEGC
LcoWntD      116 --------RGPLFGRH---ILYNSSKESAFVHTLMAASVAHEVAKACSEGR--L--EGDC
SciWntD      112 --------SGPLFGRH---ILHNSTREAAFVHALMAASVAHESAKACSEGR--L--GRDC
ScoWntX1     111 --------EGPLFGRH---ILYNGTREAAFVHALMAASVAHESAKACSEGR--L--LGDC
LcoWntF      137 --------SGPLFGRA---IKHRGTKEAAFVHSLMAASFAHESTVACKKNT-----LGDC
SciWntF      136 --------QGPLFGRA---IKQKGSREAAFVHALMAASLAHEAVVACSRGN-----LEDC
LcoWntQ      192 -SDN--HKSKPLFGQH---IIQSHTREAAFLHALMAASAAHEMAQACKAGK-----VQSC
SciWntQ       50 -SAE--HANKPLFGEA---VTRLHTREAAFLHALMAASSTHETAQACSQGK-----LSSC
HdujWntD      90 --------GAYLLGRA---IETKESKESAYARTLISAAITYDIATACRENI-----IPNC
HdujWntE      95 --------GAYILGRG---VETIDSREGAYVRSLISAAILFDVAVACREGL-----LPQC
HdujWntF     115 --------RQLVFGNF---VHTMSVRERAAVVSMVSGAVMHDLTTCCREKK-----LVDC
HdujWntH     116 --------RQLVFGNI---VHTMNLRERAAVLMMISASVMMELTTCCREAL-----LQDC
HdujWntI     108 ---G--SNRGYLFEQ----LQKSSTPESGYLRAAVASSIIYEIAADCRQGF-----IPNC
HdujWntG     110 --------ESFLLGQE---VESTANKEREYVRVLLAASITYDVAEQCRKEH--I--PACP
OloWntII     123 ------------FGN----AFTAGSRQAAYVRSLVGVAVTYSITIACSYGS--L--PLTC
OcaWntIIa    110 --------GSHLFGP----VYNSGTREAAYLRGILNVAVSYSIVMACADGS--M--PWTC
OcaWntIIb    117 --------GPYLFGP----VYRAGTRETGYLRALLGAAGTYAMVVACADGA--M--PWTC
OcaWntIIc     92 --------GPYLFGP----VYRAGTRETGYLRALLGAAGTYAMVVACADGA--M--PWTC
OcaWnt5bX    102 --------GKDVFGP----IVNQDGKESAAIHAIISAMAAYVITRECARGN--I--PLLC
OcaWnt6X     145 --------GRDVFGP----IVRQGVKESSAIYALVSAMAMYTITRECAMEA--I--PRLC
LcoWntK       88 --------LPY--------MASRTFRETAFLQALLAATLTSTLVRSCRDGS--T--DESC
SciWntK      119 --------LPF--------LSRKATRESAYLNALMAAALASGISHACHRGT--A--AVPC
LcoWntU       34 -------NTSRVFGKA---VLKGTTRESAFLHSLMTAAVMRSIARSCGQGL--L--GDRC
SciWntU       80 -------NSSKLFGDA---VLAGTTREGAFLHALMTASLIQSIARSCGKGY--L--ADRC
SciWntM      107 -------STPFLFGKG---ISKGSHRESAFIHALISASVTHGVARACSAGR--L--GRLC
ScoWntX3      92 -------LDTTLFGRG---AVKGTHPESAFIHALLAASVTHGVATACSQGK--L--GNLC
LcoWntH      114 --------WNV--------FLSKIHKEAAYLHAVISASVASQIILDCKKGN--L--PSVP
SciWntH       95 --------WKV--------FLGKTHKEAAFVHAVISIAVSAQLVHDCNNGR--L--PSTT
ScoWntX4     114 --------WNI--------FLGKLHKEAAYVHAIISIAVSAQLVKDCNNGL--L--PSTT
LcoWntB       81 --------FVPLL------MSSATTREGAYLKALTSVMVAYEIAWACTSGY--L--QDVC
SciWntB       89 --------LVPMLKD------TTNMPQAAFMKALTSAMVAYEIAQGCYYGY--T--KLNC
SciWntO       22 --------RSM-LRN----ALRQGSPEAGFVAASQMAGVAYSLLEACKREQ--FA-GGYC
HdujWntK     113 --------APLFLGQMQ-PFVHHLTKETAFTQALLSALSFIKVARGCTSET-----FTSC
HdujWntJ     126 --------APLFPGHIQ-PFMSHLTRETAFVQALLSAMYFSRLAKG----------FRSC
LcoWntP      129 ------------WKR----FLSRAHPEAAYFRALLSSNLVFQAARHCAIKQ-----DALC
SciWntP       99 ------------WKK----FLLRAHPEAGYFRAALSASVVFHTARHCAMGK--I----TC
ScoWntX2     106 ------------WKK----YLLRAHPEAGYFRAALSASLVFHTARHCAMGK--I----SC
LcoWntL      176 -------NRYPFFGQHSPPRRLAGTKEQAFLSALEAAGSVVKLARKCSDGSH-L--GREC
SciWntL       90 -------SRYPFFGVDNPPKLLKGTKERAILSALDSAGVLVKLSRECNKGG--P-PLRAC
ScoWntX5     156 -------SRYPFFGKNMPPKRLRGTRERAILSALDSAGALVKLSRECSRGT--TDSFAAC
LcoWntS      100 SSAD--GIPPPYFGR----FIDATTRQAAFIHALSTAVLLQEMVRS----------EAPC
SciWntS      158 WSSDVPALPPPYFGK----FLDLSTRQAAFIHALSTAVLLQEMISK----------EVPC
```

```
                  *
AquWntC      141 ECGKNTT-----------------------------------------------------
PfiWntC      161 ESQTTTG-----------------------------------------------------
HdujWntC     120 SCGRTAT-----------------------------------------------------
AquWntB      137 SCDTSRQ-----------------------------------------------------
PfiWntB       82 GCDTRFN-----------------------------------------------------
EmuWntB      128 GCDTSMS-----------------------------------------------------
SlaWntB      128 GCDTSMA-----------------------------------------------------
EfrWntB      128 GCDTSMG-----------------------------------------------------
AquWntA      137 TCQINGD-----------------------------------------------------
EmuWntA        8 KCETVGX-----------------------------------------------------
SlaWntA      147 QCEIVGD-----------------------------------------------------
EfrWntA      148 ICETIGD-----------------------------------------------------
CreWntA      154 QCSIDAP-----------------------------------------------------
PsuWntA      144 PCLRGFL-----------------------------------------------------
CcaWntX1     142 GCDRSNAFRSY-------------------------------------------------
HdujWntL     149 SCDQTVR-----------------------------------------------------
OloWntI      124 GCGREDD-----------------------------------------------------
OcaWntI      132 GCGRVRG-----------------------------------------------------
CcaWntI       67 GCDRSWI-----------------------------------------------------
LcoWntA      145 GCDKDWT-----------------------------------------------------
SciWntA      143 QCDQTLT-----------------------------------------------------
ScoWnt-i     150 GCDKTLT-----------------------------------------------------
SciWntN      153 ACNKSQQ-----------------------------------------------------
ScoWnt-ii    155 GCDKLSM-----------------------------------------------------
SciWntT      178 GCATGSE-----------------------------------------------------
LcoWntE      160 GCDTTWT-----------------------------------------------------
SciWntE      183 GCDTTWT-----------------------------------------------------
ScoWnt-iii   152 GCDTTWT-----------------------------------------------------
LcoWntR      176 GCDQLWR-----------------------------------------------------
SciWntR      183 GCDKIWK-----------------------------------------------------
LcoWntC      142 GCDKSVL-----------------------------------------------------
SciWntC      139 GCDKSVL-----------------------------------------------------
ScoWnt-v     139 GCDKSVL-----------------------------------------------------
LcoWntI      150 SCDTSVK-----------------------------------------------------
SciWntI      144 SCDTSVH-----------------------------------------------------
ScoWnt-iv    143 SCDTSVH-----------------------------------------------------
LcoWntJ      138 GCDDSPRYRTG------LVGNTNGATLL-----AIDPANP---------------------
SciWntJ      146 GCDDSPQYRAG------QRGSSSGATSLS---SANRPAQG--------------------
LcoWntG      145 GCDLTYR-----------NYGEEQRNKFQHRSTRLHGRGE--TPTDSSDGTLDLEYDTVT
SciWntG      142 GCDSSFK-----------QYGREQHEKFQQRSSRIRAEAESSSPGGSGEVNLDLDGDII-
ScoWnt-vi    144 GCDSTYR-----------NYGKEQRDKFQQRSAKLRSAGESDK---GDQVDMDLDGDII-
OcaWnt2X     197 GCRKISS-----------------------------------------------------
EmuWntC      132 GCSQFNT-----------------------------------------------------
SlaWntC       27 GCSAFNT-----------------------------------------------------
EfrWntC      179 GCATFNS-----------------------------------------------------
CreWntC      192 SCPEKEG-----------------------------------------------------
CcaWntX2     220 GCASNRI-----------------------------------------------------
LcoWntD      161 RCKTNHHEAHLLLGLI--------------------------------------------
SciWntD      157 NCVTNYHEEDQLRGLV--------------------------------------------
ScoWntX1     156 NCVTNYHEDTDLKAIV--------------------------------------------
LcoWntF      181 GCQRPDS-----PTPMLLEKSAEELQY-----ALKAPLL---------------------
SciWntF      180 GCVRPQL-----PAPTQKDIEQYELTQFGQSGAPVAPRA---------------------
LcoWntQ      241 TCGHFELVEIH--MDGKKNVTSAPT-------TTALPATASA------------------
SciWntQ       99 GCSMPDQAELSALYPNTSSSSSGPSIHDVITASSQRPSSAVT------------------
HdujWntD     134 PCAHDFP-----------------------------------------------------
HdujWntE     139 PCHADSP-----------------------------------------------------
HdujWntF     159 SCNFSLP-----------------------------------------------------
HdujWntH     160 SCHFPPD---------------------------------------------------I-
HdujWntI     154 PCGSVHK-----------------------------------------------------
HdujWntG     155 SCSIHGG-----------------------------------------------------
OloWntII     163 GCLSRSK-----------------------------------------------------
OcaWntIIa    154 GCLARTK-----------------------------------------------------
OcaWntIIb    161 GCLSRSK-----------------------------------------------------
OcaWntIIc    136 GCLSRSK-----------------------------------------------------
OcaWnt5bX    146 GCARTRQ-----------ELSKDQW-REIL------------------------------
OcaWnt6X     189 HCASTPK-----------SISQDEW-RKTV------------------------------
LcoWntK      128 SCEAEHRRRIA-----MVEAGRDNST----------------------------------
SciWntK      159 SCLAVHEDRKA-----EADGERDNST----------------------------------
LcoWntU       80 PCGQYEG-----------------------------------------------------
SciWntU      126 PCGQDAG-----------------------------------------------------
SciWntM      153 TCAVGGL-----------SVSRDDP-----------------------------------
ScoWntX3     138 TCSAGVL-----------------------------------------------------
LcoWntH      154 ICGSGSL-----------------------------------------------------
SciWntH      135 ICGTNPS-----------------------------------------------------
ScoWntX4     154 ICGGSPS-----------------------------------------------------
LcoWntB      123 GCSQGQG-----------------------------------------------------
SciWntB      131 YCPVVAG-----------------------------------------------------
SciWntO       66 GGNATTL--SD-------------------------------------------------
HdujWntK     159 KCMAGSS-----------------------------------------------------
HdujWntJ     167 DGERVEN-----------------------------------------------------
LcoWntP      168 YADNRTAQEMFA------------------------------------------------
SciWntP      137 YMGNTTNSKNPS------------------------------------------------
ScoWntX2     144 YLGNTTEGGHPS------------------------------------------------
LcoWntL      226 SCPGVFG-----------------------------------------------------
SciWntL      140 QCLGSTT-----------------------------------------------------
ScoWntX5     207 SCIQSAT-----------------------------------------------------
LcoWntS      144 GAHQAS------------------------------------------------------
SciWntS      204 GAHCSILNRE--------------------------------------------------
```

```
                                   *
AquWntC     148 -------ISSAGNQVMY-GCSSN----WEFGMEMSA--KFMDGKEKHGVV----------
PfiWntC     168 -------ITVNETHSVT-SDLHD----VTYSAILAE--KFLDSIENGGKS----------
HdujWntC    127 ------LPTGAGQWYFA-SCSEN----IGFGQEVSR--RFLDEAEHDRG-----------
AquWntB     144 ------GQTSPQGWQWG-GCSDD----VGFGVMLTR--AFLDTRNNATNK----------
PfiWntB      89 ------GQETQQGWQWG-GCSDD----VEFGANFAH--MFLDVRETENIE----------
EmuWntB     135 -------ALTQLGWDWG-GCSHD----VNYGVQYAQ--SFLDARETTNQTT---------
SlaWntB     135 -------ALTQSGWDWG-GCSHD----VDYGVQYAQ--SFLDARETINQTT---------
EfrWntB     135 -------TPSQSGWDWG-GCSHD----INYGVQYAQ--MFLDAREATNQTA---------
AquWntA     144 ------NGVVNSTFFLY-ECSFD----IAKAHDIMS--KFLETPSNDDTA----------
EmuWntA      15 ---GVRNSGCPGKHHLN-DCSDN----TGYATDTMN--QFIRDNSTN-------------
SlaWntA     154 ----VRTQDAQGNIIFN-DCSDN----IQYASNTVL--QFIKDNSTN-------------
EfrWntA     155 ----VRTQDAQGNIIFN-ECSDN----IKFASDIVR--QLTRENSTN-------------
CreWntA     161 -----RTVDDENNIIFE-TCKAD----FNFSSEYFG--EFIAAQIS--------------
PsuWntA     151 -----VNTTANGDTIFS-DCAAN----FEWAANFFS--SFVTSVYEQL------------
CcaWntX1    153 -VSSGKNPRWDFDFDWD-DCSDN----IKFGNEFAS--EFLRQPNKG-------------
HdujWntL    156 ------SPPTDGSFLWS-GCGDN----YEYGYYFSQ--RFSNSAYQLTDTS---------
OloWntI     131 --------EAGDDWDWG-GCGDN----LDQGRESSA--RFLRDDVKSP------------
OcaWntI     139 -------EKEGETWEWG-GCGDN----LEYGIDYTT--RFISNRERVP------------
CcaWntI      74 -----KKTHQYGDWFWG-GCGDN----IEYGVEFSR--NFILARTPDK------------
LcoWntA     152 -------SKKPVGYSWG-SCSDN----FVKGELFAR--EFLSARKNE-------------
SciWntA     150 -------KNKAKDWTWG-PCTDN----YHKGKQYAK--EFLDAGKNE-------------
ScoWnt-i    157 -------AKESPDWTWG-SCSDN----FHKGEEFAK--QFLDGSRND-------------
SciWntN     160 --------AVGQNWTWG-SCSDD----FQSGDKFAG--EFLSGMVKA-------------
ScoWnt-ii   162 -------EKKPGNWTWG-SCSDN----FNKGSQYAA--EFLSDVYMANR-----------
SciWntT     185 ------LGNPEGNWTWA-SCSDN----IADGIRFSR--EFLNGREDG-------------
LcoWntE     167 -------KHEAVNWTWG-SCSDN----FVKGYELGK--QFVDASETDV------------
SciWntE     190 -------RNEAVNWTWG-SCSDN----YVKGYELSK--QFLDAGETDT------------
ScoWnt-iii  159 -------QTEAVNWTWG-SCSDN----YVKGYELSK--QFLDAGETDA------------
LcoWntR     183 -DGGKSGVQTNSTWSWG-ACSDN----MHYAYKKTF--QFLTSGVHPAR-----------
SciWntR     190 -QGGGSDIKPNASWSWG-ACSDN----IHFAHTQTL--NLLAGPNEENGD----------
LcoWntC     149 -----DDDSNVGNWEWG-GCGDD----FEKGRQYSR--MFLDIGAKETLPASPSPPVTPM
SciWntC     146 -----DEETTEEEFEWG-GCGDN----FLKGQQYSQ--QFLDLAAKDLPA-------PSE
ScoWnt-v    146 -----DDDTNAGEFEWG-GCGDN----FQKGKQYSQ--QFLDLAAKETPA-------TVE
LcoWntI     157 --DSTIEHNIKGKFSWG-GCGDN----FAKGYQFAK--EFLDVAHLSQQ-----------
SciWntI     151 --DENIEHNIKGKFDWG-GCGDN----FAKGYQYSK--EFLDVAHTEEEQ----------
ScoWnt-iv   150 --NEDVEHNIKGKFDWG-GCGDN----FAKGYQYSK--EFLDVAHTADQG----------
LcoWntJ     167 -----RTDKTGGAFVWG-GCGDN----VNTGYSYGK--EFLGDERTFSKS----------
SciWntJ     177 -EPTGAADATAGKFVWG-GCGDN----VDTGYAYGR--QFLGDGQVFSPT----------
LcoWntG     192 DPGEVLLQKEDEAWRWG-GCGDN----WEAGMRVAA--EFLDSGAISDLR----------
SciWntG     190 --NSVPLTEDDQSWVWG-GCGDN----WEAGMRFAA--EFLDTGAVLNRSK---------
ScoWnt-vi   189 --KAVPLTGKDQSWSWG-GCGDN----WEAGMRYAA--EFLDAGTITNRT----------
OcaWnt2X    204 --------DDKGVTEWE-GCDDD----VKVGSKFSR--DFVVASLRG-------------
EmuWntC     139 ----NNMAQVSGNTYSG-NCSDN----LDFGYRFAM--NFTTSGVTS-------------
SlaWntC      34 ----NTMAQVAGDSYSG-NCSDN----MEFGYQFAL--NFTTSGITS-------------
EfrWntC     186 ----NNMPQGDGNTYSG-DCSDN----FEFGYQFAL--NFTTSGITS-------------
CreWntC     199 ------------------GCPDP----VTYGLHIAA--TFLNMRYTSSG-----------
CcaWntX2    227 -----PETHSDDNFQWG-SCSDD----VYFGANVSA--MFLNSQEHKR------------
LcoWntD     177 ---NATEDIALAEYRWK-QCDND----MAHGLRTAK--KFQSVSPAPGVW----------
SciWntD     173 DREQEAARWATADYRWK-NCDNN----IAYGLRMAK--KFQSVEPRPGVW----------
ScoWntX1    172 DSEQEGFYLTRSEYRWK-NCDNN----ILYGLRMAK--KFQSVEPRDGIW----------
LcoWntF     210 -TKQKFDPEKNYRLEWTTGCFDN----TKYGSHKAE--EFVVRSSAFVK-----------
SciWntF     214 GETHLDDKKLNISYVWSAKCFDN----ILYGMSKAD--EFVQKSSRYVK-----------
LcoWntQ     274 AIHPSARTSMKVKQLRA-GCSDN----IEYGVENTV--DFQLRSEHAQAAVQSG------
SciWntQ     141 SAMVTERSLAKHKRVRP-RCGDN----IEYGVRASR--EFHRTIEVEQSD----------
HdujWntD    141 -----FVTFEGDIVKFG-GCSDN----YGYAIEVNK--AFTGGEEEAHKN----------
HdujWntE    146 ----YIRKLSDGTILLN-GCGAN----PDYAKRTAD--VFLDSPGPLPLQ----------
HdujWntF    166 GIIQQSFVEGKLVAVYG-GCSDH----LATPKARTM--TLLKMDPRSTSS----------
HdujWntH    168 LRFERIVVNGNATYIYA-GCSDR----LDIPAERTK--QLLDVNSNVED-----------
HdujWntI    161 ----FIELLPNGTRIIG-GCDVN----IHHAATVAE--PFLGAGLGA-------------
HdujWntG    162 ---LPFVEPISGGVKLR-TCSVN----LQWAAQVAR--LFMGQDSAD-------------
OloWntII    170 -----LPPFGNRTYEWG-ECSHD----VSRAATLAS--NFIIAGEEGEDE----------
OcaWntIIa   161 -----LPQFDNATYEWG-ECSHD----VNLGVNLGS--AFTSAGEVKEEQ----------
OcaWntIIb   168 -----LPPFDNYTHEWG-ECSHD----VRRGIDLAC--SFIRAGEVETEQ----------
OcaWntIIc   143 -----LPPFDNYTHEWG-ECSHD----VRRGIDLAC--SFIRAGEVETEQ----------
OcaWnt5bX   164 -PNDFFQRHNDPDFSWG-GCTED----VDFGYKTTK--EFLDSSYDAWLSTVPPP-----
OcaWnt6X    207 -PAEILSRDENSAFSWG-GCTEN----LDFGYKVSK--RFLDLSHKDC------------
LcoWntK     149 -LLSMWENNELEDWLKT-ECSDN----IKYATRFSR--DFTNAQEDA-------------
SciWntK     180 -VAAMQDDARVQRWLNG-KCNDN----IAHARKFSQ--DFMDRQVDT-------------
LcoWntU      87 -----STIPGNRDWHWG-NCSVN----LVYGQKMVR--KISNPINARRQN----------
SciWntU     133 ---ASFHNGQAQNWEWG-NCSVN----MVYGRNLVR--RLANPPNARRQN----------
SciWntM     167 -SGTQGRSSAAFDWAWS-RCGDD----TDYGVRIAK--ALASKPRES-------------
ScoWntX3    145 ---NRNKNNTPVDWQWS-QCSLD----TAYGIRIAK--ALASRSRTA-------------
LcoWntH     161 -------------CNSQ-GCAVEV---ISYGYNTAKILDWLHSPAQLPHM----------
SciWntH     142 -------------CQGS-SCTKEI---ITYGYSTAKILDWLNSPDQLQSM----------
ScoWntX4    161 -------------CQGS-SCTKEI---ISYGYNTAKILDWIHSPEQLDSM----------
LcoWntB     130 ---------TYVNFSYGRVCRNI----ETTAHRVTK--RLLDDNTDAILQ----P-----
SciWntB     138 -------KSEKGVNNAF-ECAQV----MGSGVSGAK--LYLDTDHIAELLQPF-------
SciWntO      75 ---RARGAAGSAAARWG-Q--------LQHTLQTLRVYMLLDDEAENDLA----------
HdujWntK    166 ------LGHLNRNEKWD-LCMDN----TVAANQATQ--RFLDEMWQDTRF----------
HdujWntJ    174 --------TAENLRRWV-HCQEA----QSSSV------DLLDGMWSTNQ-----------
LcoWntP     180 -GLVRRPVFSDVEKGES-FCVYH----FKRGLQVAKVLDIVDKLSDFSIN----------
SciWntP     149 -HLEYKLFFSDFAKKQT-FCDYH----FTEGLRAAKILDILDGFFFHAMS----------
ScoWntX2    156 -QIEYTMFFNDFAKKQS-FCDYH----FTEGLRAAKILDVVDKYFFAMMN----------
LcoWntL     233 ---------RQERFEYS-MCTDRSRPGPQTIYGVAA--SFADAHEEEICH----------
SciWntL     147 ---SAGTTQEEFVRHTV-ECSRN-EDASSGPMAIAK--NFVDANEHEICK----------
ScoWntX5    214 ------NRQDSFAQAAE-LCAYS-EKTMFGPMFIAK--NFVDHNEDEICH----------
LcoWntS     150 ------------------RCMPDN---FECCAELVS--RFVRGGELKGRKI---------
SciWntS     214 ------------PCEMT-RCTTQN---FKCCQDLVE--IFVSAGDRKGRKM---------
```

```
AquWntC     184 ---------------IGDRQ---LINLQNNQVGRTVFL-------------DVN--HKKE
PfiWntC     204 ---------------LSDRQ---HINLHNNRLGRMVVQ-------------N-----SVK
HdujWntC    163 ---------------LGLRQ---LSRLQDLAVGRAVLA-------------QLA--AAMG
AquWntB     181 ------------TGNELEAS---LVNLHNNAVGRTVVS-------------D-----NMQ
PfiWntB     126 ----------EKSHGDLGLS---LVNLHNNAAGRKEVQ-------------D-----EMD
EmuWntB     172 ---------------DIGTAPVEVVNLHNNAVGRQTVQ-------------D-----YMQ
SlaWntB     172 ---------------DIGTA---LVNLHNNAVGRQTVQ-------------N-----YMQ
EfrWntB     172 ---------------GIGTA---LVNLHNNAVGRQTVQ-------------D-----YMQ
AquWntA     181 -----------------------IIAEHNHNVGSNLVG-------------------QRY
EmuWntA      52 ---------------TSDLD---LVNTHNYQVGLKMIS-------------------QRN
SlaWntA     190 ---------------ISDVD---VVNYHNYQVGLQVLS-------------------QRN
EfrWntA     191 ---------------ITDVD---LVNNHNYEVGLRIIA-------------------NRN
CreWntA     195 ---------------DSFEG---RIDQHNIDLGKEAIN-------------------HET
PsuWntA     187 ---------------DLVGV---KSDLHNVNAGILSVK-------------------KIR
CcaWntX1    192 ---------------QLARE---LMNKHNEQAGLEVIS-------------S-----AAN
HdujWntL    194 --------GGEGGQEISDYE---LMAQHNAEVGRLVVK-------------S-----NLQ
OloWntI     164 ---------------SPERR---LMDDHNIKAGIEIAV-------------R-----ETK
OcaWntI     173 ---------------DKARA---LMNDHNIAAGTQIAL-------------N-----TVK
CcaWntI     110 ---------------DLARE---NMDKHNVIAGGKITQ-------------D-----SAV
LcoWntA     185 ---------------SSPQS---VVTDHNYQTGREVVR-------------Q-----SMQ
SciWntA     183 ---------------SSPIS---MVASFNHETGQTVVR-------------Q-----SMQ
ScoWnt-i    190 ---------------SSPVS---LVENYNHQTGRLVVG-------------Q-----SLQ
SciWntN     192 ---------------NDSTS---TIAGLNHRFGRKVVG-------------K-----SMQ
ScoWnt-ii   197 ---------------SSPLS---AVTRHNYDIGRKIVG-------------K-----SMQ
SciWntT     219 ---------------RSPSS---LMSIHDTEAGRKIVR-------------S-----SML
LcoWntE     201 ---------------SSPQS---KMTLWNYEAGRLAVR-------------R-----LMR
SciWntE     224 ---------------STPKS---KSTLWNNEAGRLAVR-------------R-----LMR
ScoWnt-iii  193 ---------------TTPKA---LTTLWNNEAGRLAVR-------------R-----LMR
LcoWntR     224 ---------------KTERE---LMAVHDYKLGLEVAK-------------E-----KMQ
SciWntR     232 ---------------RAHQQ---MMERHNYRMGLEIAK-------------D-----RMQ
LcoWntC     197 STASARRPPADTSPRSRLSE---LIHKWNSRQGRLTVA-------------D-----TLV
SciWntC     187 TTTET----VAGTPQSSVVE---EYHKWNNRRGRLAVA-------------D-----NLA
ScoWnt-v    187 PTATA----AAPSTRPQLSE---EVHKWNNRRGRMAVK-------------N-----SLA
LcoWntI     197 -------------VNQTRRD---ILELHNNEAGRVVVR-------------S-----TLQ
SciWntI     192 -------------GNQTALD---VMELHNNEAGRVVVE-------------S-----TLQ
ScoWnt-iv   191 -------------LNQTKFD---VLELHNNEAGRAVVR-------------S-----TLQ
LcoWntJ     205 ---------SSLPSGEISSQ---LMAQQNHEAGRQAVK-------------S-----TVR
SciWntJ     219 ---------TSQPN-EVASQ---LMAQQNHEAGRQAVR-------------A-----SIR
LcoWntG     235 -KEKL----GGMYGKSLLVN---RMALHNNKAGRLAVR-------------R-----LLD
SciWntG     232 -VDKH----GGMFGKSPLVN---RMALHNNKAGRLAVK-------------R-----LLD
ScoWnt-vi   230 -TDRY----GGMFGKSPVVN---RMALHNNKAGRLAVK-------------R-----LLD
OcaWnt2X    236 ---------------ESLRN---IITEHNSNVGRRTVK-------------R-----LMN
EmuWntC     175 ---------------TTVQA---KTDLHNFKAGINAVK-------------DVM--AATP
SlaWntC      70 ---------------ANVQA---KTDLRNFNAGINAMR-------------DVM--AATP
EfrWntC     222 ---------------TTVQA---KTDIHNFNAGINAVR-------------DIMVPTVTP
CreWntC     224 ---------------GGLKQ---ELVIRNFRATEIIIQ-------------SVM--SSTF
CcaWntX2    263 ---------------RDLRT---QVNLHNNKAGRLTVI-------------S-----ERR
LcoWntD     217 ---------------DRKRQ---LINAHNEKLGRQLIK-------------M-G--FDER
SciWntD     216 ---------------SRQRK---LINLHNEKLGRTIVR-------------K-G--FERA
ScoWntX1    215 ---------------SEERK---LMNLHNEKLGRSLIK-------------A-G--FHRA
LcoWntF     252 ---------------RRARQ---LMNLHNHRAGLKAVE-------------------KTR
SciWntF     257 ---------------RRARQ---LMNLHNHQAGILTLK-------------------ETK
LcoWntQ     321 EISSD----SPTCASHKRRS---IMDAHNEEAGRQILL-------------R-----HMK
SciWntQ     184 ---SS----SPRCTARFSRT---LMDRHNEEAGRQIVK-------------R-----NTK
HdujWntD    179 ---------------VELSA---FINSHNNELGRKVVS-------------------ESY
HdujWntE    185 ---------------DGLAV---KTLDHNRLVSEQVID-------------------SPH
HdujWntF    209 -------------SSEKENCT---SADVHNAHVGVQMAS-------------------QTK
HdujWntH    210 ---------------EEPCD---LADRHNVEAGIKMAS-------------------VTT
HdujWntI    197 ---------------MATRE---AVDHHNTELGKLIGS-------------------STE
HdujWntG    199 -----------------------GTARHNIDYGIAWVN-------------K-----SLS
OloWntII    208 ----------ETSADDRLNS---LANVHNYEAGTKIAT-------------T-----AVK
OcaWntIIa   199 ---------------TKLNY---LSNLHNRDVGQKVAR-------------E-----GAK
OcaWntIIb   206 ---------------TRLNY---LSNLHNHDVGTKVAR-------------D-----GVR
OcaWntIIc   181 ---------------TRLNY---LSNLHNHDVGTKVRKSRGSLFGRTPWIQS-----ATA
OcaWnt5bX   211 EMPFSWPKAIEPAPAYTPSQ---LTDLHNEEAGRLVVT-------------K-----SRK
OcaWnt6X    247 ---------------SICNR---SVHLRNEEVGRAIVK-------------E-----GLK
LcoWntK     188 ---------------SRKSG---AFAIYNYNVGREVFN-------------S-----LVE
SciWntK     219 ---------------SKKNG---VFALYNYKVGREVFN-------------S-----RVK
LcoWntU     125 --------------QLSPRS---LLQVLNNAAGELALE-------------S-----VQR
SciWntU     173 --------------QLRPRP---LMQVLNNAAGELALE-------------S-----VQR
SciWntM     206 ---------------SSRRK---RLHHHNYVAGYQVPV-------------E-----MEQ
ScoWntX3    182 ---------------TSRKR---KVHLHNYAAGYHVTT-------------G-----QEY
LcoWntH     194 ---------------KRRQA---IVHNHNRDVGRFIVK-------------K-----HIK
SciWntH     175 ---------------KWRQA---LIHNHNRDVGRVVVK-------------K-----QMK
ScoWntX4    194 ---------------KWRQA---LVHNHNRDAGRFIVK-------------K-----QIR
LcoWntB     166 WIANQ----PSFRAKYRKHR---GVKLHNNRVGRKLFA-------------R-----EAV
SciWntB     177 -NKIY----PSGGAIHSTQE---QVRFHNNRKGREVFK-------------R-----MAR
SciWntO     113 -----------------------KARRLNSDIGLQVVL-------------N-----MMS
HdujWntK    203 ---------------PDEER---LVQIHNLEAGRRAVMA-----------PE-----NYR
HdujWntJ    204 ---------------DDEQR---LVHIHNLEAGRRAVTA-----------PE-----NVM
LcoWntP     224 ---------RRRKHTKKFDL---VAHYHNNAVGRQIIN-------------SL----RPK
SciWntP     193 -------KSKKRRWSRSRDL---HTNYYNNAVGREKIK-------------DI----RPH
ScoWntX2    200 ---------KKRRWSKKRDL---LTHYHNNAVGREKIK-------------DI----RPH
LcoWntL     271 --------GSKRVGADPRLC---KVNLHNNNVGRTILI-------------K-----NFV
SciWntL     190 --------GSARAGRDETQC---MTNLHNNLVGRRLVV-------------R-----HMR
ScoWntX5    254 --------GTQTHGPDITYC---MTNLHNNMVGRKMVV-------------R-----SVD
LcoWntS     178 -----------SDETHRNRR---MVNEHNRAVGMAAFR-------------TW----GLK
SciWntS     247 -----------TNETYTNRV---MINQHNRMAGIKAML-------------SR----GLK
```

```
                     *  *      *    *
AquWntC       211 PTCKCVGVSASCSAKTCQRGLE---AFSVVAASIK-DKYKKSCKVSV-----------K-
PfiWntC       228 YGCRCEGLSASCSLQTCQTTLA---NLRTVSAKIY-QAYDNSCKVRT-----------N-
HdujWntC      190 PQCRCQGLSGACSIRTCRTMTP---PLGAMGQQLL-SRYGQACRVTL-----------E-
AquWntB       208 VKCRCHGASGSCATRTCYSQLP---TVRDISTDMK-IKYNHSVKVTA-----------H-
PfiWntB       155 VECTCHGISGSCSVRTCWRKLP---ELRSVSKNIK-QKYDQSIKVSL-----------Q-
EmuWntB       199 TSCSCHGISGSCTVQTCWRQLP---EVGAIGDVLR-QKYEAAAMVRV-----------D-
SlaWntB       196 TSCSCHGISGSCTIQTCWRQLP---EVGVVGDVLR-QKYNAAAMVRV-----------G-
EfrWntB       196 TSCSCHGISGSCTIQTCWRQLP---EVGAVGDALR-QKYDTAVMVKV-----------D-
AquWntA       199 RKCRCTGFSGSCSVQTCYFASP---DIDTIGQRVR-EKYGSSVEVTV-----------N-
EmuWntA        75 TTCLCHGVSGTCTVQTCYQQVP---DVATIGGTLR-QKYTNAVKVTR-----------V-
SlaWntA       213 STCLCHGVSGTCTVQTCYQQVP---DVSSFGDTLS-WKYINAKQVTR-----------V-
EfrWntA       214 ASCVCHGVSGTCTVQTCYQQVP---DVATFGDIIR-WKYINAVKVSR-----------V-
CreWntA       218 TNCRCHGISGTCTVQTCYKRVP---TVPEIGEQLF-IRYGGAIHVVD-----------S-
PsuWntA       210 KSCNCHGISGTCTVQTCYDQVI---SVSEVRDSLA-ISYNGATKVEL-----------V-
CcaWntX1      216 LSCFCHGFSGSCALRTCWLEAP---SMNRIGSELK-KAYNKAVKVKR-----------V-
HdujWntL      225 VQCRCFGLSGTCSIKICHAALP---NFDLVGQKLY-GSYGNAKKVVR-----------S-
OloWntI       188 RNCRCHGLCGACAIKSCWKELPR--NFHQIGAVVE-NKFDGSVKMKL-----------N-
OcaWntI       197 KNCRCHGLCGGCSFETCWKELPG--KFHNVGEKVK-SKFNGASIMKF-----------N-
CcaWntI       134 MECQCHGFSGSCTVKSCWKQLP---SMSKIGLLVK-REFDGAVKVTL-----------N-
LcoWntA       209 LVCKCHGLTGTCAQKTCWYSLP---KVQVVGKTML-QKYDIPVRVKW-----------S-
SciWntA       207 LVCKCHGLTGTCAQKTCWYSLP---KVQVIGKKVL-NKYTRGLRVKW-----------E-
ScoWnt-i      214 LVCKCHGLTGTCAHRTCWYSLP---KVQVIGEKLL-TKYDAAMRVKW-----------E-
SciWntN       216 FLCKCHGFSGSCVQKTCWYSLP---TIGVVAEQLL-RKYDKIFRVVW-----------N-
ScoWnt-ii     221 FLCKCHGVTGSCSYKTCWYGLP---KIQLIGEQLA-KKYDKIFQVVW-----------N-
SciWntT       243 RVCKCHGLTGTCAHRTCWYSLP---KIHVVGTSLL-DKYDKSIKVIP-----------R-
LcoWntE       225 LQCKCHGLTGSCAHQTCWYSLP---LVRVVGSQLL-KKYEDSTIVRL-----------D-
SciWntE       248 LQCKCHGLTGTCAHQTCWYSLP---LVRVVGNLLM-AKYESSTAVKM-----------S-
ScoWnt-iii    217 LQCKCHGLTGTCAHQTCWYSLP---VIRVVGKLLM-KKYEASTTVKS-----------A-
LcoWntR       248 FLCKCHGLTGSCVHKTCWYSVS---KIQIIGRALM-RKHAKSVKVKA-----------N-
SciWntR       256 FLCKCHGLTGSCVHKTCWFSVS---KIQIIGRALM-RRYGRSIKVAP-----------D-
LcoWntC       236 TRCKCHGLCGACTVKICWKEVP---SVTKIGKVLK-DSYRDAKPVRL-----------A-
SciWntC       222 TVCKCHGLCGDCTVKICWKEVR---SVQ-IGEVLK-ESYREAKQVRL-----------A-
ScoWnt-v      222 RVCKCHGLCGDCTVKICWKEVR---SIK-IGKVLK-EAYHDAKLVHL-----------A-
LcoWntI       223 TICKCHGLTGACNVKICWRSLP---KVQVVGLILK-DKYRNAMQVSV-----------T-
SciWntI       218 TICKCHGLTGACSVKICWRSLP---KVQVVGLILK-DKYRNALQMGI-----------S-
ScoWnt-iv     217 TICKCHGLTGACSVKICWRSLP---KVQVVGFILK-EKYRNAMEVAV-----------P-
LcoWntJ       235 RTCKCIGLTGSCTKKICWRSLP---TIQVVGELLM-ESYRNARQVKL-----------K-
SciWntJ       248 RTCKCIGLTGACTSKICWRSLP---TIQIVGEILM-ENYRNARQVKV-----------K-
LcoWntG       269 IRCRCIGLSGSCSEKLCWRVLP---SIQRVGERLK-KKFFSAVQVEM-----------N-
SciWntG       266 IRCRCMGLSGSCSEKVCWRVLP---SIQRVGDRLQ-KKFTAAVKVRM-----------A-
ScoWnt-vi     264 IRCRCMGLSGSCSDKVCWRVLP---SIQRVGERLK-KKFSVAVKVRL-----------D-
OcaWnt2X      260 KQCKCHGASGSCTIQSCWNEVP---SMEKVGILLE-KKYRNAVNVEV-----------V-
EmuWntC       202 PKCKCLGLSGSCTTQVCWQEAP---DFSVVGSSIK-KLFDSACVVSW-----------N-
SlaWntC        97 PNCKCVGLSGSCTTQVCWQEAP---DFSVVGGSIK-KLFDSACLVTW-----------N-
EfrWntC       251 PKCKCIGTSGSCTTQICWQEAP---DFSVVGSSIK-KLFDSACQVSW-----------N-
CreWntC       251 KKCSCHGISGSCTFSVCHSELP---PFSTLAKRVK-QAYNDSCLVLP-----------N-
CcaWntX2      287 LQCKCHGTSGSCALRTCWMSLQ---RFHEVGRHIV-HQYDQAVQVVS-----------N-
LcoWntD       243 PKCKCLGFTGSCATKICWREVP---NFRDIGNKIS-ELYTSAKQVQL-----------S-
SciWntD       242 PVCKCLGFTGSCLTRMCWREVP---NFRAVGNALF-SLYRSPVETTL-----------N-
ScoWntX1      241 PVCTCLGFTGSCATRVCWREVP---NFRQVGNTLF-RLYSRAQRVQI-----------S-
LcoWntF       275 LKCRCHGLTSSCTVLLCWNEVQ---HFTAIGNALK-EKYEQAIRMRLV--IEP-KVPVSA
SciWntF       280 MKCQCLGVTSSCSFRVCWNEVE---HFTKISKQLL-QMYERAIPMKLVAIVDPEKLPPNV
LcoWntQ       356 LKCRCMGISNACNIEVCWREVP---SFTKIGNLIM-ERYKNATQLKA-----------D-
SciWntQ       216 LRCRCLGISNSCSTQVCWKQLP---VFTKVGNLLM-SRYRNASLMKL-----------D-
HdujWntD      202 TDCRCHGQTGSCNIRICYKRLK---PFTGADALTY-DKYRGAVRVTL-----------A-
HdujWntE      208 VVCKCHGLTNDCSIKTCVRHLR---PFKQTSESML-QKYKSAIKVHA-----------G-
HdujWntF      235 IHCHCHGLTASCNVKTCKHRML---PYTEVASSML-MKYEGAVKVVS-----------S-
HdujWntH      233 RSCHCHGLTAACNIQTCKHRIL---RYSNVANQML-EKYSGAVKVQK-----------E-
HdujWntI      220 KYCRCHGLSASCSIKVCHQKLS---SFKTTHPEILHTKYRQSQLVKS-------------
HdujWntG      218 ERCHRRNIFTTPRVLICHYGID---------NVLN-PKQPSRIAVAN-----------SV
OloWntII      237 VVCRCLGGTLSCATKVCHRELR---QFSHIGSTLV-DKYNKAVQIKL-----------S-
OcaWntIIa     223 LICRCIGGTFSCATKVCHLELK---VFSYFGTKLK-EKYDSAVQVKL-----------S-
OcaWntIIb     230 VVCRCIGGTVSCATKVCHLELA---DFAYISDELK-KKYLSAMKVKL-----------S-
OcaWntIIc     218 RLCKCV------------------------------------------------------
OcaWnt5bX     250 IHCRCHGACGACNFQTCWEEMK---DWEEVGDDIK-KKYDGAVKVKM-----------N-
OcaWnt6X      271 VNCKCHGPSSSCAVQTCWKEMK---RWEEVSNDIK-EKYDNPVKVTNC----------N-
LcoWntK       212 TKCECYGVSGSCDLRTCWRRAP---TPEEVAREIF-KRYLSALRTKK--QF----LHLS-
SciWntK       243 KKCKSYGVSGSSTLHTCWWQEP---SIEEISAELY-HRYGNAIKTKM--HF----LPTK-
LcoWntU       150 IKCRCHGVSSTCALKTCFMAAP---DLHSTGQTLA-LKYKKSKKVSI-----------Y-
SciWntU       198 ISCRCHGVSSSCIMRTCIMVTP---DLSTTAQTLA-KKYRQAKKVIV-----------A-
SciWntM       230 VICSCYGHSGSCTQKFCHVKQQ---RARTVSLRLM-KQYSRATLVQV-----------R-
ScoWntX3      206 IGCSCYGPSGSCTHKFCHVRLA---SENALAGKLW-KEYRRAVQVEI-----------K-
LcoWntH       218 ENCKCQGISASCIQWHCAHAIP---QPALMIRNLT----RGAFRFLP----------WK-
SciWntH       199 KTCDCNGISASCLEMRCYHRLP---STREMVSGLM----NNAVFLPW-----------K-
ScoWntX4      218 KRCKCNGVSGSCLENRCYHRLP---EMSEMIRGLM----DNKVFLPW-----------K-
LcoWntB       201 DVCKCHGLSGGCELQLCIKQQP---SKRVLTQALS-QLYLNARQISF-----------D-
SciWntB       211 VKCTCHGLSGACNLKTCVRTPA---RPAAIARELR-RMYDNARFMQP-----------Q-
SciWntO       132 EYCGCRPSPGATCSLTCLGSTL---SFSRVSKTIM-SLYYSSVLLET-----------N-
HdujWntK      229 KECKCHGMTGDCISKTCFKKVK---SPRVIAEFIKRDLYHKAEKVALLD---------K-
HdujWntJ      230 NYCRCHGPTTSCTVKTCQWKLN---YRSHIGTYIKETLYPQARKVVAVRA--------G-
LcoWntP       255 LHCVCIGPSAGCSMTTCFYILQT-KLVKDSGHEIY-NQYRSLRDNPS-------------
SciWntP       226 LRCFCSGPSGTCVLKTCSYVETK-RMLTRTADALY-QQYQKFVNGGT-----------A-
ScoWntX2      231 LHCYCTGPSGSCILKTCSYVETK-RMLTRTALALH-EQYTQLLAGGA-----------P-
LcoWntL       302 RKCSCLGMSGACTVQNCITYMPDILKSRELEYDFK-RAFNQAQQIYV-----------K-
SciWntL       221 RKCHCVGTSGSCLDQVCVKYMPDILHEAPLKREFL-RSFNQAKQVYV-----------S-
ScoWntX5      285 RRCQCIGVSGSCLHKSCIGYLPDILNDKSIKKDFG-RSFNQAKQAYV-----------S-
LcoWntS       207 VIKKCFGMSGSCAQKKKKTLV----RRAPVSKPLL-ELYDKAAYVKVVL---------D-
SciWntS       276 EQQNCFGISGVCSFKHTHTII----RAEQAAQYIR-GLYDRADEIKIVK---------D-
```

```
AquWntC     255 ---ASA----------------L------------------------QPHQCNSSSI---
PfiWntC     272 ---IINSDEP-------------------------------------SFISTDCDKI---
HdujWntC    234 ---PAGPHT-----------HTL------------------------ALRPACGGSI---
AquWntB     252 ---VNRG-T-----------TVLRST---------------------SSSNTEAVSP---
PfiWntB     199 ---VQKD-EP----------PSL------------------------KSVGDDPMPP---
EmuWntB     243 ---IPRDGSP----------ASL----------------------YYTDSMQNPVAP---
SlaWntB     240 ---VPPGGGP----------ASL----------------------YYADSGPNPVVP---
EfrWntB     240 ---VPRDGGP----------ASL----------------------YYANSGQNPVAP---
AquWntA     243 ---ASNS-A-------------LQPV-------------------------VQTINN---
EmuWntA     119 ---SGTT-T-------------LRPVY--------------------------SATL---
SlaWntA     257 ---PGTT-T-------------LRPVY--------------------------SASL---
EfrWntA     258 ---PGMT-T-------------LKPVY------------------------NQNSAL---
CreWntA     262 ---DGEF----------------------------------------ASSNPNIDPP---
PsuWntA     254 ---NGELQR-------------I------------------------PIPGANPDNI---
CcaWntX1    260 ---VTDDGK-----------KKL-------------------------VNKLDFQPL---
HdujWntL    269 ---SGTP--------------VLTPV-------------------------DSRTPL---
OloWntI     233 ---SGGG-E-------------L------------------------EVAERNHVPP---
OcaWntI     242 ---KTTV-S-------------L------------------------QVDDRGQVPP---
CcaWntI     178 ---DADR-E-------------L------------------------IPENPRHIPP---
LcoWntA     253 ---QPRT-N-------------IIPY-------------------------SALIDN---
SciWntA     251 ---AQTE-Q-------------IKPY-------------------------SALVTN---
ScoWnt-i    258 ---QPSE-S-------------LVPY-------------------------SALVSN---
SciWntN     260 ---STEM-E-------------VAPFASD------------------PLKQSLKQSR---
ScoWnt-ii   265 ---ATQG-K-------------VESF----------------------TKDPLKKSR---
SciWntT     287 ---ADRR-K-------------LVPL------------------------RATSKRP---
LcoWntE     269 ---LPRK-E-------------LVVK----------------------KAIAASKKL---
SciWntE     292 ---SSGSPR-------------LVVK----------------------RTTATALPL---
ScoWnt-iii  261 ---SVSTRQ-------------LVVK----------------------RISLTRPPI---
LcoWntR     292 ---LART-R-------------L------------------------ERLDMGGKRP---
SciWntR     300 ---SAKQ-K-------------LVPQ-------------------------DTERKV---
LcoWntC     280 ---HPQAPL-------------LLPAG--------------------VSNVNAAQLPL--
SciWntC     265 ---HPLSPA-------------LVPANV-------------------ENNAIAASAPN--
ScoWnt-v    265 ---HPLSPA-------------LAPVGIP------------------VNSDGVATAPD--
LcoWntI     267 ---PPSPSDRTTSTTATPARQRL------------------------RPLDRHAGTP---
SciWntI     262 ---EAPDVDSPPHEEGSREPPRL------------------------VPLDHLAREA---
ScoWnt-iv   261 ---ELSDTPGTDIETT----RRL------------------------VALDPLVPRP---
LcoWntJ     279 ---TKKNGP------------KLLPL---------------------EKDEDGKSKV---
SciWntJ     292 ---AKKNAL------------RLTPL---------------------DRDEGVKTRI---
LcoWntG     313 ---PSVPNG-------------LYSIPS-------------------NPRVTRRKAP---
SciWntG     310 ---PRSRTG-------------LAMVMDAA-----------------IGEESRDVKP---
ScoWnt-vi   308 ---EKSRTG-------------LLVLPEDG-----------------TGLAAAGRRP---
OcaWnt2X    304 ---KSGSNT-----------KNL----------------------FKFIPMDSSKKP---
EmuWntC     246 ---QYLGTNS-------------------------------------NWLSNVCPII---
SlaWntC     141 ---QYLGTNS-------------------------------------NWVSSMCPLI---
EfrWntC     295 ---QYLGTNS-------------------------------------NWISPACPVV---
CreWntC     295 ---GHSR----------------------------------------NDWVAQCDHPI--
CcaWntX2    331 ---QMKHDQ-------------LTSVSR-------------------YTFSSIRSHP---
LcoWntD     287 ---TDGT-R-------------LIKVT--------------------QFADKVNEGP---
SciWntD     286 ---AAGD-G-------------LQSVYMATVDAAAGAE-QPTVVDGGAGDGVTLPAP---
ScoWntX1    285 ---DDAS-E-------------LVKAQQ-------DIS-VPTVAGA-TATPAIADPP---
LcoWntF     328 GE-QHKTENA----------TAIEPLFTSGR---RNLGLRKNVNWYLVPAEQDGKYP---
SciWntF     336 TGIPENSTE-----------AQLAPAVLVKK---LKVS-QKDIRWELRPAQAGAAYP---
LcoWntQ     400 ---KTGS-R-------------L------------------------VLANALSRKP---
SciWntQ     260 ---RHGT-R-------------L------------------------VVRHALSRQP---
HdujWntD    246 ---NHPSPMA-------------------------------------GQVVVQGGSL---
HdujWntE    252 ---VGEA--------------PL--------------------------SSWQGDDP---
HdujWntF    279 ---PDGL---------------L------------------------KPLNPNADPP---
HdujWntH    277 ---AETN-Q-------------L------------------------LPANALADPP---
HdujWntI    264 --FQQGT---------------L--------------------------ISTNQDAV---
HdujWntG    257 --FQNGV-Q-------------L-------------------------------------
OloWntII    281 ---KNGE-R-------------L------------------------KSADSTTGTF---
OcaWntIIa   267 ---KNGE-R-------------I------------------------KNSDSATKPF---
OcaWntIIb   274 ---KNGE-R-------------L------------------------KNEDSTAKAF---
OcaWntIIc   224 ----------------------V-------------------------------------
OcaWnt5bX   294 ---HKNT-K-------------L------------------------INSDAKVKKH---
OcaWnt6X    316 ----------------------LAPVPAN-------------------------------
LcoWntK     261 ---RARN-------------RSL-------------------------THRQLSLTP---
SciWntK     292 ---RRQS-------------KTL-------------------------TFRQKIRSP---
LcoWntU     194 ---RGSVDR-----------RPLLHL---------------------ARAVKQGRTL---
SciWntU     242 ---RGSPDR-----------RPLLQL---------------------ARAVKQHARPARQ
SciWntM     274 ---RRSSGG-----------WRL--------------------------KEVDGGKP---
ScoWntX3    250 ---PRPNGG-----------WRL--------------------------KQVGAAKP---
LcoWntH     260 ---NNRD-VV---------HSAL--------------------------KRFRREDPQ--
SciWntH     240 ---EHRN-QV---------HHSL--------------------------KTVRRRRS---
ScoWntX4    259 ---ENRN-RV---------QGSL--------------------------KSVRGKR----
LcoWntB     245 ---TAIN---------------L-----------------------------ENSNV---
SciWntB     255 ---QARN---------------L-----------------------------RHVRP---
SciWntO     176 ---SSTD-------------------------------------------GTSQENT---
HdujWntK    276 ---LHGA-E-------------L------------------------FTVKPIPTKP---
HdujWntJ    278 ---GHGSLR-------------L------------------------IDAETQTRSP---
LcoWntP     300 ---AVGAAR-----------RHF----------------------KRIPESIDHDRE---
SciWntP     272 ---AVESAR------------ALIAEA------------------QAQPRPATVATH---
ScoWntX2    277 ---AIASAR------------KLVKS---------------------IQRGSRTRGQ---
LcoWntL     349 ---SHISYVGK--------KKELKNKRVS------------------LWRSGDNMEV---
SciWntL     268 ---KGQSGK-----------LRL-------------------------SKATDHKKP---
ScoWntX5    332 ---TSHSGV-----------KKL-------------------------VSSATHIPL---
LcoWntS     252 -PYSLRP--------------VIVYKTWERP--------------AVAMQIEPVDTP---
SciWntS     321 --YLNTP--------------QIVKKNAQIS--------------HHRIPIHDDDKP---
```

```
                                        *                                    *
AquWntC      269 ------SNTTLVHTLSS--PDYCHKDIS------KGSFGVQGRLCDPA------------
PfiWntC      289 ------TNNTLIFFDNS--VDYCYRDIS------VGSPGVKGQSC---------------
HdujWntC     253 ------ATDTLIYSDSS--PNYCLPDLT------RGSYGTRGRRCDRR-A----------
AquWntB      273 ------PVDSLVHVKNS--VKYCIRQND------Y----TANRSCIPQ-NILTQIESNEA
PfiWntB      218 ------STSHLVFLKKS--KNMCLYKQN------Y----TLGRSCVPK-NILTEYHSSGI
EmuWntB      265 ------SSTEMVYLEPT--VDYCSQQSNY-----------TLNRYCIPR-SNMTS------
SlaWntB      262 ------MSSEIVYLEPT--VDYCSLQSNY-----------TLNRYCVPR-SNMTS------
EfrWntB      262 ------SRTEIVYLEPI--IDYCSTQSNY-----------TLNRYCIPR-SNLTS------
AquWntA      258 ------HDNELVYLKRS--PTFCNQDTT------YGILGTVGRQCSNN-L----------
EmuWntA      133 ------NESDLAYLADS--PTFCTADNN------MGILGTSGRQCNPT-S----------
SlaWntA      271 ------NQSDLAYIAGS--PDFCTANND------MGILGTSGRKCNPT-S----------
EfrWntA      274 ------NESELAYIADT--PDLCVANNN------LGILGTSGRKCNPS-S----------
CreWntA      276 ------DDNTIIFKDNS--PNFCVENRQ------LGTVGVANRICNPN-S----------
PsuWntA      271 ------NENNLVFLDNT--PDLCKTDLA------KGILGTAHRLCKEE------------
CcaWntX1     278 ------NTPELAYLSNS--QDFCTADKM------TLQPGTIGRECNVSIS----------
HdujWntL     284 ------GQTDLVYLLDP--PSYCDRDDR------YGSLGTQGRYCKVD-S----------
OloWntI      249 ------SNFDLVYLESTDYSRFCVKDSS------VGSHGTHGRLCDPE-S----------
OcaWntI      258 ------TVTDLIFLETTDPTQFCSKNVG------IGSHGTSGRLCDPD-S----------
CcaWntI      194 ------TDSNLVYLTKS--SDYCKYDPS------TGSHGTIGRECNKT-S----------
LcoWntA      268 ------ITTELVYSKGS--PDYCVPNEE------TGSLGTSGRECDPL-K----------
SciWntA      266 ------VTDQLLYSTPS--LDYCEANDG------TGALGTAGRQCDPN-K----------
ScoWnt-i     273 ------LTAQLVFDTPS--VDYCEANAD------IGAPGTQGRVCNPR-L----------
SciWntN      282 ------MTEKLVYQQPS--PDYCEPNKA------IGSLGTHGRECEPR-I----------
ScoWnt-ii    283 ------MNEKLVFKDTS--VDYCQPNMA------IGSLGTHGRECVPK-V----------
SciWntT      303 ------KDDDLIHLQDS--PDYCTSNST------VGSLGTLGRKCSPK-E----------
LcoWntE      287 ------SQEDMVHLRRS--PDYCFSDGS------AGSLGTQGRICDGT-K----------
SciWntE      311 ------TTEDLVYLRRS--PDYCLSDPA------AGSLGTFGRTCKGK-K----------
ScoWnt-iii   280 ------THEDLVHLRRS--PNYCVSDPE------VGSLGTFGRTCQGK-K----------
LcoWntR      308 ------QVKDAVHLHDS--PSYCVRDES------IGSLGTFGRKCGTR-S----------
SciWntR      315 ------DIKDSLHLHQS--PSYCDRDPV------VGSLGTAGRECGQR-R----------
LcoWntC      302 ---ATLATKNLVYMKES--PDFCMANTT------LGIPGTARRQCIKD-S----------
SciWntC      288 ---PILTTNALVYVKES--PDFCVANGT------IGVAGTARRRCKEN-S----------
ScoWnt-v     289 ------ITSALVYVKES--PDFCVANAS------IGVSGTARRRCNKN-S----------
LcoWntI      297 ------KSSELLYLAPS--PDYCKPNAS------IGVNGTAERECRAE-S----------
SciWntI      292 ------EPAELTYLHTS--PDYCRPNAT------IGIKGTGERECNLE-S----------
ScoWnt-iv    287 ------DESELTFLHSS--PDYCKPNAT------IGINGTAARECKVE-S----------
LcoWntJ      300 ------KSEELTYTRPS--PNFCRRNMT------SGVEGTRGRECDEL-S----------
SciWntJ      313 ------VATELSYVRQS--PDYCRPNTT------LSISGTRGRQCQEL-S----------
LcoWntG      335 ------PSKDLVYAETS--PSFCELSQL------RASPGTQGRQCKPW-S----------
SciWntG      334 ------PSKDLVFSETS--PSFCIHDQL------KASPGTTGRKCKPL-S----------
ScoWnt-vi    332 ------PSKELVYSETS--PSFCNANQL------KASPGTKGRRCKPW-S----------
OcaWnt2X     325 ------REDQLVYQSDS--PSYCNDNRR------AFSLGTSGRECNST-A----------
EmuWntC      263 ------TDRTLIYGGQS--PNWCYADPS------VGSMGVTGRQCDPN-S----------
SlaWntC      158 ------TDRVLIYGSQS--PNWCIPDPT------VGSTGVVGRQCDPN-S----------
EfrWntC      312 ------TDKTLIYGGQS--PNWCYPDPT------IGSLGVVGRQCDPN-S----------
CreWntC      310 ------TDSDLLF-TKS--NTWCKYDPE------IGSAGVVGRECDPH-P----------
CcaWntX2     353 ------DSLDLVYYEKS--PRYCVRNKL------VGSLGTKGRKCEQN-S----------
LcoWntD      307 ------APTDLVFSAAS--YSFCDSIPP------LDVFGRTGRVCQKN-S----------
SciWntD      325 ------SENQLVHGSDE--WSYCDANSE------FDIAGTRGRICQTG-S----------
ScoWntX1     316 ------SEFELVQGADT--LSFCDANPS------LDIAGTPGRTCQES-G----------
LcoWntF      371 ------TEDDLIYLDDS--ADYCVENQE------MGIPGTKGRQCHDA-E----------
SciWntF      378 ------SAHELVYIDQS--PDYCKRNDF------LGVQGTGGRQCHRD-D----------
LcoWntQ      416 ------SKEDLVFIDAS--PAFCREQRS------LDVLGTSGRECRNT-A----------
SciWntQ      276 ------SSTDLVYLGES--PSFCSDNPG------LDIVGTSGRECRVS-Q----------
HdujWntD     263 ------QRDDMIYSDQS--PSYCESNQE------MNILGTHGRQCDTS-H----------
HdujWntE     266 ------TPNDLVYTQDS--PNYCKANPA------INILGTSERVCHFD-E----------
HdujWntF     294 ------TDMDLIYFTDS--PDLCAINAT------MDVPGTKDRKCDPN-T----------
HdujWntH     293 ------TSGDLVFFCDS--PDYSVANAS------MDIPGTKDRLCDPS-K----------
HdujWntI     278 ------TAETMAHLDPS--VNSCHMH------------STVGKQCDSI------------
HdujWntG     264 ---WNGNLDRFTYLTPS--VDYCCDNRQLSWGSPTTIPWTRGRECVPRDS----------
OloWntII     297 ------EDTDLVY-ANS--ASLCEPNSA------FGYDGIHGRECISDDP----------
OcaWntIIa    283 ------DDGDFVYGTDG--GSLCEPNPA------IGYEGVHGRECSKD-R----------
OcaWntIIb    290 ------DKSDLVYGTDG--GSPCLVNPE------FGYEGVHGRNCSKD-R----------
OcaWntIIc    225 ------------------------------------------------R-----------
OcaWnt5bX    310 ------IHSDLVFLQDS--PNLCTENGE------LGVEGTQGRKCNGT-S----------
OcaWnt6X     323 ------KIPNLVCQNES--PDLCVKNDA------VGVEGTCNRKCSST-S----------
LcoWntK      277 ------KRDQLVFMKHS--PDFCTSQPT------LGFPGTYGRRCEETFT----------
SciWntK      308 ------RPGQLAYLRSS--PDYCTADAS------RGIPGTRGRRCERTFS----------
LcoWntU      216 ----NLTEYMLHYTDPS--PDFCTKDVR------GGIPGNTGRPCSSH-K----------
SciWntU      267 HVGLNISLYQLHYTNES--PDFCTPDPV------RGIPGTRGEVCSKT-V----------
SciWntM      291 ------SKNSLVYSTPS--PDYCQPDVA------LGYPGTKDRECTND-S----------
ScoWntX3     267 ------SKETFVYSTLS--PNYCEQNVH------LGYPGTQGRKCLDD-E----------
LcoWntH      279 ------GKTTLVYMKKS--PSYCCADNS------QGITGTEGRTCFGK------------
SciWntH      258 ------GKTTLVFMKRS--PSYCCTDRS------QGILGTEGRSCNSH------------
ScoWntX4     276 ------GKTFLVYMKRS--PSYCCSDPS------QGTLGTEGRSCNGQ------------
LcoWntB      255 ------SNKALVYIHDS--PNFCKRRPF------YGHFGVTGRRCNPS-G-------HLA
SciWntB      265 ------APTQLLYTAHP--PSMCQPLPD------LGFPGIQNRQCTMPFS----------
SciWntO      187 ------TNISLVHL-QT--PDYCQESHA------LGSPGTRGRRCEMR-TIAQEV----V
HdujWntK     292 ------SHTSLVFTEDS--PNYCVSNSIE-----YGALNVSGRECVES-Q----------
HdujWntJ     295 ------PHDMLIYTEE---PDYCSESASV-----FGAVDSSNRRCELP-G----------
LcoWntP      321 ------KPPELAAPRAS--PNYCTSSKR------HGILGQHRRSCVAF-QCGTK------
SciWntP      296 ------RNMSVIATRHS--PNYCRRNSN------WGLLGPRGRSCREACG----------
ScoWntX2     298 ------RNITVIASRPS--PSYCRPSKK------WGILGPGNRICYTACG----------
LcoWntL      377 ------TDTQLTYYRPS--PKLCHFHHR------HIAPPVGERNCSLA-P----------
SciWntL      286 ------GNTELVYFRPS--PRLCSHQFR------PIGPPVHHRYCKNITG----------
ScoWntX5     350 ------RSDQLAFFQSS--PILCSHNKR------PIGPPVGERYCNAT-A----------
LcoWntS      280 ------QPTEMIYLKKS--PSFCHGK--------YGLAKPSGRPCEEE-G----------
SciWntS      348 ------RKEQLIYARKS--PNFCRRR--------YGLPGPSGRQCDID-G----------
```

```
                               *   *  *               * *              *   *
AquWntC      303 --------VASKSCETIC--CGRGHIEFTKDVE-GKC-C-KQV---------GC---CGV
PfiWntC          ------------------------------------------------------------
HdujWntC     288 ------PPTHPESCSSLC--CGRGFQLVQTTAA-YHCGC-RFE-------------NFQI
AquWntB      314 NP-THYPGYPLPACESLC--CSGEYETEEYTVS-TTCYC-HFV---------WC---CKI
PfiWntB      259 EP-LTSVDLTLAPCEDLC--CAGEYSLKRTVVV-RSCNC-HFV---------WC---CDI
EmuWntB      300 -----YLGGYYSTCEDLC--CNGQYVTVKTIRT-YSCNC-KFI---------WC---CNV
SlaWntB      297 -----YLSGYYSACEDLC--CNGKYITLQRTRT-YSCNC-KFI---------WC---CNV
EfrWntB      297 -----YLTGYYAACEDIC--CNGRFVTVRTTRT-YSCGC-KFI---------WC---CNV
AquWntA      293 --------SDPDSCDIIC--CGRGHITVTATQP-KQC-C-SFI---------YC---CRI
EmuWntA      168 --------LGLDSCFYLC--CNRGYTAKTRIVP-EEC-C-QFV---------WC---CRI
SlaWntA      306 --------QGLDSCYYLC--CGRGYTTKTTIVP-QQC-C-QFV---------WC---CRI
EfrWntA      309 --------QGLDSCYFLC--CGRGYTAKTTVVP-QEC-C-QFV---------WC---CRI
CreWntA      311 --------NSRNACASTC--CDRGHHTITKHVPIEEC---KFI-----------------
PsuWntA      305 --------PGLLDCANLC--CGRGFYTVTYTVPIEEC---RFV---------WC---CRI
CcaWntX1     314 --------SGEGSCSYLC--CGRGHTMTLILDD-REC-C-RFH---------WC---CEV
HdujWntL     319 --------VGVDSCGHLC--CGRGYRVEYLEVP-ELCNC-RLT---------DCPNSCRF
OloWntI      286 --------SGTEGCAHLC--CGRGYDTFEETDI-EKCNC-KFV---------WC---CRI
OcaWntI      295 --------SGIDRCDHLC--CGRGYTTREEVDR-FDCNC-RFV---------WC---CSI
CcaWntI      229 --------DGIDGCSLMC--CNRGFYSREVTLT-RRCKC-QFI---------WC---CHV
LcoWntA      303 --------TGPGGCQKLC--CNRGHNERRHVET-RKCKC-QFI---------WC---CHV
SciWntA      301 --------TGPGSCNHLC--CARGYNERRRIEE-RKCNC-KFL---------WC---CRV
ScoWnt-i     308 --------SGVGGCTRLC--CGRGHNERRRIET-KKCKC-KFV---------WC---CRV
SciWntN      317 --------AGPTSCDTVC--CQRGYNERRRIAS-QRCRC-RFH---------WC---CRV
ScoWnt-ii    318 --------DGTSSCDEVC--CRRGYNERRRIES-KTCAC-KFL---------WC---CKV
SciWntT      338 --------GAENHCDKMC--CGRGYNVRRRINN-EKCSC-RFE---------WC---CRV
LcoWntE      322 ------SSSGQGSCDHLC--CGRGFNVQRYTEE-EQCRC-KFV---------WC---CHV
SciWntE      346 --------SGYGGCDHLC--CNRGFNVQRYAEK-EKCKC-KFV---------WC---CHV
ScoWnt-iii   315 --------SGYGGCDHLC--CNRGFNVQRYTRK-EQCRC-AFV---------WC---CHV
LcoWntR      343 --------TLMGNCDRLC--CGRGQTTRRFIFQ-EKCDC-HFE---------WC---CRV
SciWntR      350 --------VKQSICPTLC--CDRGSMPRRAVLH-KQCNC-RFE---------WC---CRV
LcoWntC      340 --------KGEDGCELLC--CGQGFYEYRIVKR-EQCRC-KFV---------WC---CDV
SciWntC      326 --------KASDGCELLC--CGNGYFETRAVKR-EQCEC-KFV---------WC---CEV
ScoWnt-v     324 --------QGSDGCELLC--CGNGYFETRDVKR-EQCAC-KFV---------WC---CEV
LcoWntI      332 --------RGDDGCELLC--CGNGNYVKRSVLR-QKCRC-KFI---------FC---CEV
SciWntI      327 --------RGDDGCELLC--CGNGHHPQRSVLR-QQCKC-KFV---------FC---CKV
ScoWnt-iv    322 --------RGDDGCELLC--CGNGFYVKRSVLR-QKCRC-KFI---------FC---CDV
LcoWntJ      335 --------HGDDGCELLC--CGRGYYAKRTVKL-QKCRC-RFL---------WC---CKV
SciWntJ      348 --------TGDDGCELLC--CGRGYYAKRTVTT-KKCKC-RFH---------WC---CRV
LcoWntG      370 --------ADEDSCQSLC--CGRGYRTIEETLQ-ERCRC-KFH---------WC---CRV
SciWntG      369 --------PGDDSCQHLC--CGRGYTTERKRVE-ERCRC-RFH---------WC---CRV
ScoWnt-vi    367 --------KGEDNCEQLC--CGRGYTTEEKRVE-ERCRC-TFH---------WC---CRV
OcaWnt2X     360 ------NDHASTSCNFLC--CGRGYDVITEIRQ-KNCRC-HFN---------SC---CAV
EmuWntC      298 --------SGSNRCSSLC--CDRGYVETQVTQD-TDCNC-KFV---------YC---CSI
SlaWntC      193 --------SGPNQCSSLC--CNHGYVQTQIAQD-TDCNC-KFV---------YC---CSI
EfrWntC      347 --------SGTNKCSSLC--CDHGYVQTQITQN-SDCNC-KFV---------YC---CSI
CreWntC      344 --------EAPNSCNKLCGGCKRPSIQQTVEEV-VQCDC-QFL---------FC---CEI
CcaWntX2     388 --------QSTNSCLHLC--CGRGFRVKYLVEE-YDCEC-SFR---------WC---CRV
LcoWntD      342 --------DDSDSCDNLC--CGGGHKQRTVTTS-TQCSC-RFF---------WC---CSV
SciWntD      360 --------GESDSCRSMC--CYRGYQGQTVTQK-ARCNC-RFV---------FC---CSV
ScoWntX1     351 --------SESDGCGPLC--CFRGYRAATVTQH-SPCGC-RFV---------WC---CNV
LcoWntF      406 ------DDTSPGTCKRLC--CGRGHRTVTRTIR-TSCHC-RFV---------YC---CRV
SciWntF      413 -------DFSPGSCSLLC--CNRRVRTVTRVIR-QSCHC-KFV---------YC---CRV
LcoWntQ      451 -------DGSLDSCDVLC--CGRGHTTTVEHIT-TRCNC-RFI---------FC---CRV
SciWntQ      311 -------DHTPDSCDVLC--CDRGYRTVTDHTT-TSCNC-RFV---------FC---CRV
HdujWntD     298 --------AGLGSCDVLC--CGRGYWRTTKSIPIRKCET-MFD---------STTGRFEV
HdujWntE     301 --------TKPGSCQQLC--CGRGQHETERVVLKQEC---KLV---------AI-RDGNV
HdujWntF     329 --------QGTGSCSQLC--CSRGFYTKTRETNVYYT---DFD---------TT--TFRI
HdujWntH     328 --------HGLGSCDLFC--GGRGYYKRTITTPIYER---HFD---------FN--QFKI
HdujWntI     306 -----------VQCRQTC--CSSQYTERTVIVSMPIC---RLV---------ML---GGV
HdujWntG     309 ------PSGHLLGCGVIC--CGRGYQAKSQPVE----HC-RFV---------YV---SGR
OloWntII     332 --------SAPNYCPSFC--CGYGYFSYIEEKK-RSCRC-KLK---------CC---FEL
OcaWntIIa    318 --------TAENYCGSLC--CSFGYNTFVERKK-KSCRC-RIEVIVG-----CC---FDL
OcaWntIIb    325 --------QEGNYCGTLC--CGYGYKTYIERKK-KSCRC-QIKVVEH-----CC---FKL
OcaWntIIc        ------------------------------------------------------------
OcaWnt5bX    345 -------DLAEDSCSILC--CGRGYKTMTRQEV-YPCNI-RLR-------RKPP---INI
OcaWnt6X     358 -------TQPDSSCRILC--CGRGYITVTKITT-ISCNP-KLR-------RKPP---IGI
LcoWntK      313 --------SDEGSCLSMC--CGGGYNVEEVEE--EVCEC-RHV---------FC---CRR
SciWntK      344 ------TEVGSGSCYNLC--CGGGSHAETVEE--TVCDC-QII---------YC---CYR
LcoWntU      253 --------ASIHSCDVLC--CGREPAVKERRTR-RCC---RRV---------ATIRGGKL
SciWntU      308 --------GHERECNKLC--CGREPLDRKYRRG-RCC---KWG---------ITIGSGKV
SciWntM      326 -------SDDEESCSKLC--CGRGFSARTYVKM-VNCHCTRRR---------FP---WPV
ScoWntX3     302 --------SAIGSCARLC--C-RGHITNTHTRM-ERCEC-SAS---------YF---PRR
LcoWntH      313 -----------ESCKKIC--CSTTVEKVRTG---SRCNC-KYM---------YC---CSL
SciWntH      292 -----------DSCRKIC--CSEEKEEIGL----DKCNC-KFI---------FC---CEL
ScoWntX4     310 -----------ESCRKVC--CSKEVEQMGS----NMCNC-KFH---------FC---CRL
LcoWntB      293 GP---SASKDLPICKDLC--CGAGVRSKRTPV--YKEMC-RFI---------WC---CVK
SciWntB      301 NPVAFTLLPKPGACSALC--CGRNILSKDVSVV-YKSVC-DFK---------WD---TGR
SciWntO      227 NE--T-GPHQDGACQYLC--CGRGQRFETFRSP-YKCLC-SWSI-------------AGF
HdujWntK     328 ---QLYHHHRKGICDEIC--CN-GHRVVVTEEE-YICRC-VMN-----------------
HdujWntJ     330 ---QSYVQNRLGTCGKIC--CNGYVNKPKVRTQ-HRCIA-QYKQ----------------
LcoWntP      360 ---VSVNFQGTPACDDVC--CGTFETRNTS----VPCDC-KYS---------WKF--NKI
SciWntP      332 E--NVEYFTGSPACNEIC--CGRLHTHVTE----YQCDC-KLED--------WDL-----
ScoWntX2     334 RP-NGEHFGDTSHCQDIC--CGRSGSQVTE----YACNC-RMQKKDA-----WVL-----
LcoWntL      412 ------QGTVIGSCKYLC--CGYKPKRVVTQVK-KPCNF-VLQKVAG-GGARYTY-----
SciWntL      322 ------PGIVPGSCEYLC--CGGQFEKEIEKVP-YTCNA-RLRRMPNAEGYRWIF-----
ScoWntX5     385 ------EDGAIGSCHYLC--CGGPVETVTEVTE-RRCNA-TLVK----DGTRYSY-----
LcoWntS      313 -------NPDGITCAGLC--CPGGVVRRRNMNV-VKSQC-VFV---------FC---CRI
SciWntS      381 -------TDDGLTCDILC--CGRGYLTRVVEYHFPQCKPEQQL---------PC---CYT
```

```
                       *   *             *
AquWntC     338 ------QC-NDC----KRTL-TFYACR---------------------
PfiWntC         ------------------------------------------------
HdujWntC    325 ------KC-DEC----QSTV-TLSVCR---------------------
AquWntB     357 ------SC-EEC----EKTL-TRYKCTG--------------------
PfiWntB     302 ------IC-DDC----AVTV-DTYKCTS--------------------
EmuWntB     339 ------VC-STC----TETM-VQYKCTS--------------------
SlaWntB     336 ------VC-STC----TETV-VQYMCTG--------------------
EfrWntB     336 ------VC-NTC----TETV-TQYKCTS--------------------
AquWntA     328 ------EC-QDC---GEETF-TEYFCK---------------------
EmuWntA     203 ------EC-TVC---RNNTV-TDYFCN---------------------
SlaWntA     341 ------EC-TNC---KNVTM-TDYYCN---------------------
EfrWntA     344 ------EC-TYC---KNVTM-TDYYCN---------------------
CreWntA     341 ----------------------------X-------------------
PsuWntA     340 ------DC-AVT---GSKTI-VERRCNP--------------------
CcaWntX1    349 ------RC-TTC----RRQR-EAAICD---------------------
HdujWntL    358 ------RC-DTC----NKLE-PHEFCN---------------------
OloWntI     322 ------EC-EKC----YRKV-KRSYCKE--------------------
OcaWntI     331 ------KC-DKC----EKRV-KRSYCKE--------------------
CcaWntI     265 ------KC-ETC----KERV-IKHFCN---------------------
LcoWntA     339 ------RC-QTC----KGVK-RWYTCK---------------------
SciWntA     337 ------VC-SRC----LTVN-KHYTCR---------------------
ScoWnt-i    344 ------EC-ERC----RTVS-KRYTCV---------------------
SciWntN     353 ------EC-DWC----RVVS-KKYTCW---------------------
ScoWnt-ii   354 ------HC-KQC----RTVT-KKYTCL---------------------
SciWntT     374 ------KC-KRC----RVAK-QSFTCM---------------------
LcoWntE     360 ------QC-KTC----TVHK-ERHTCN---------------------
SciWntE     382 ------RC-RTC----LENK-ERHTCN---------------------
ScoWnt-iii  351 ------RC-KTC----TVNK-ERHSCN---------------------
LcoWntR     379 ------KC-DTC----ERAG-EIYWCE---------------------
SciWntR     386 ------RC-DTC----EEVE-ETYKCKR--------------------
LcoWntC     376 ------KC-DWC----EPVY-QQFYCKTPKRTSA--------------
SciWntC     362 ------KC-QWC----HKVY-RYFHCKMPRPQVA--------------
ScoWnt-v    360 ------KC-QWC----HRVY-QNFHCKQPRLQVT--------------
LcoWntI     368 ------VC-DSC----IVAW-ETHHCNGPRAIE---------------
SciWntI     363 ------VC-DYC----LVAW-ETHHCNDPLQLTARK------------
ScoWnt-iv   358 ------VC-DSC----LVAW-ETHHCNGPLLTSAK-------------
LcoWntJ     371 ------VC-DEC----ITAT-ETFLCN---------------------
SciWntJ     384 ------VC-DQC----ISAE-ETFHCN---------------------
LcoWntG     406 ------TC-DRC----VSRK-KIHVCN---------------------
SciWntG     405 ------TC-DRC----VSVQ-EIHTCN---------------------
ScoWnt-vi   403 ------TC-DRC----VSTQ-EIHTCN---------------------
OcaWnt2X    398 ------KC-ETC----TSTK-EKYICK---------------------
EmuWntC     334 ------QC-SKC----HTVT-TTYVCL---------------------
SlaWntC     229 ------QC-SKC----HTVT-TAYVCL---------------------
EfrWntC     383 ------QC-LKC----HTVK-TTYVCL---------------------
CreWntC     382 ------KC-EIC----TERR-TYFSCS---------------------
CcaWntX2    424 ------EC-KTC----RRKT-PYHVCR---------------------
LcoWntD     378 ------KC-STC----TKVH-EETTCAPE-------------------
SciWntD     396 ------KC-EEC----TTEF-ERNTCN---------------------
ScoWntX1    387 ------KC-DTC----TNSY-KRNTCS---------------------
LcoWntF     444 ------EC-QTC----EHTI-TEHFCN---------------------
SciWntF     450 ------EC-QTC----TRQI-TEHFCADV-------------------
LcoWntQ     488 ------WC-STC----ESVR-RRHFCN---------------------
SciWntQ     348 ------RC-DQC----ETVR-RRHFCR---------------------
HdujWntD    338 ------LC-SVT---GNDIE-NRYFCN---------------------
HdujWntE    338 IKGFSPKC-TDV----PRRI-REYVCN---------------------
HdujWntF    365 VQ-------KLL---RTDTV-REHFCN---------------------
HdujWntH    364 VN-------RIV---RYETN-TKHFCN---------------------
HdujWntI    338 FR-------NTCVVTGTRMA-TQYTCQ---------------------
HdujWntG    344 FQ-------HIC---GRKMM-EKYYCR---------------------
OloWntII    368 ------IC-DVC----IVER-TKYRCK---------------------
OcaWntIIa   358 ------RC-DTC----YDDV-EKYRCK---------------------
OcaWntIIb   365 ------VC-DTC----FDEI-ERYRCK---------------------
OcaWntIIc       ------------------------------------------------
OcaWnt5bX   384 ------VW-DDC----IRTA-TEYFCL---------------------
OcaWnt6X    397 ------EW-GTC----YKLI-TEHFCR----CHST-------------
LcoWntK     348 ------VC-ENC----RTEIKTVHRCN-------------------GL
SciWntK     381 ------VCTKNC----RTVRRTIRKCN-------------------QF
LcoWntU     290 GW----KCVKPC----VSVV-RHYSCR---------------------
SciWntU     345 GS----RCRRYC----IYVY-KSYYCG---------------------
SciWntM     364 ------RC-QSC----REEE-LRYFCK---------------------
ScoWntX3    337 ------VCKKVC----QVQV-HEHFCK---------------------
LcoWntH     344 ------RC-EICQE-------KVFRCKRPTQCGDSTPSIAPG-RRDE-
SciWntH     322 ------KC-EKCLR-------RQYRCKKST-CSSSTSSRS----RDEL
ScoWntX4    340 ------QC-EECRV-------RMYRCMAPT-C--ETNSRS----RDD-
LcoWntB     333 ------ECVNEP----TGTYQTTHHCR---------------------
SciWntB     345 ------KC-VKS----RMTS-AFYYCV---------------------
SciWntO     267 ------KC-QTC----HATR-RLYFCL---------------------
HdujWntK    363 ------DCQTTC----TRKV-VRNFCQ---------------------
HdujWntJ    367 ---------DFCQHSYTRLM-DSYFCK---------------------
LcoWntP     399 ------LCKTTCHR-------QDTVCS-------------------GE
SciWntP     370 ------RC-KIC----SRAV---TVCA---------------------
ScoWntX2    376 ------VC-QSC----SREV---TACLPPE------------------
LcoWntL     456 ---------DMC----NVST-VHYKCPSSNGLSRTRKRG-----R---
SciWntL     367 ---------DTC----YKEV-HKFKCSERKERQRASPSSIA-------
ScoWntX5    426 ------QF-QVC----RDEH-LRTKCKSRRPSNRGRRSSGVTTLR---
LcoWntS     350 ------PC-AMT----TKVFVTQTYCR---------------------
SciWntS     420 ------SC-VQS----SNRR-VMTFCR---------------------
```
