# Supplementary figures and images for: Wnt signaling and polarity in freshwater sponges
Source: BMC Evol Biol. 2018 Feb 2;18:12. doi: 10.1186/s12862-018-1118-0 (PMC5797367; doi:10.1186/s12862-018-1118-0)

A

PhyML raw tree

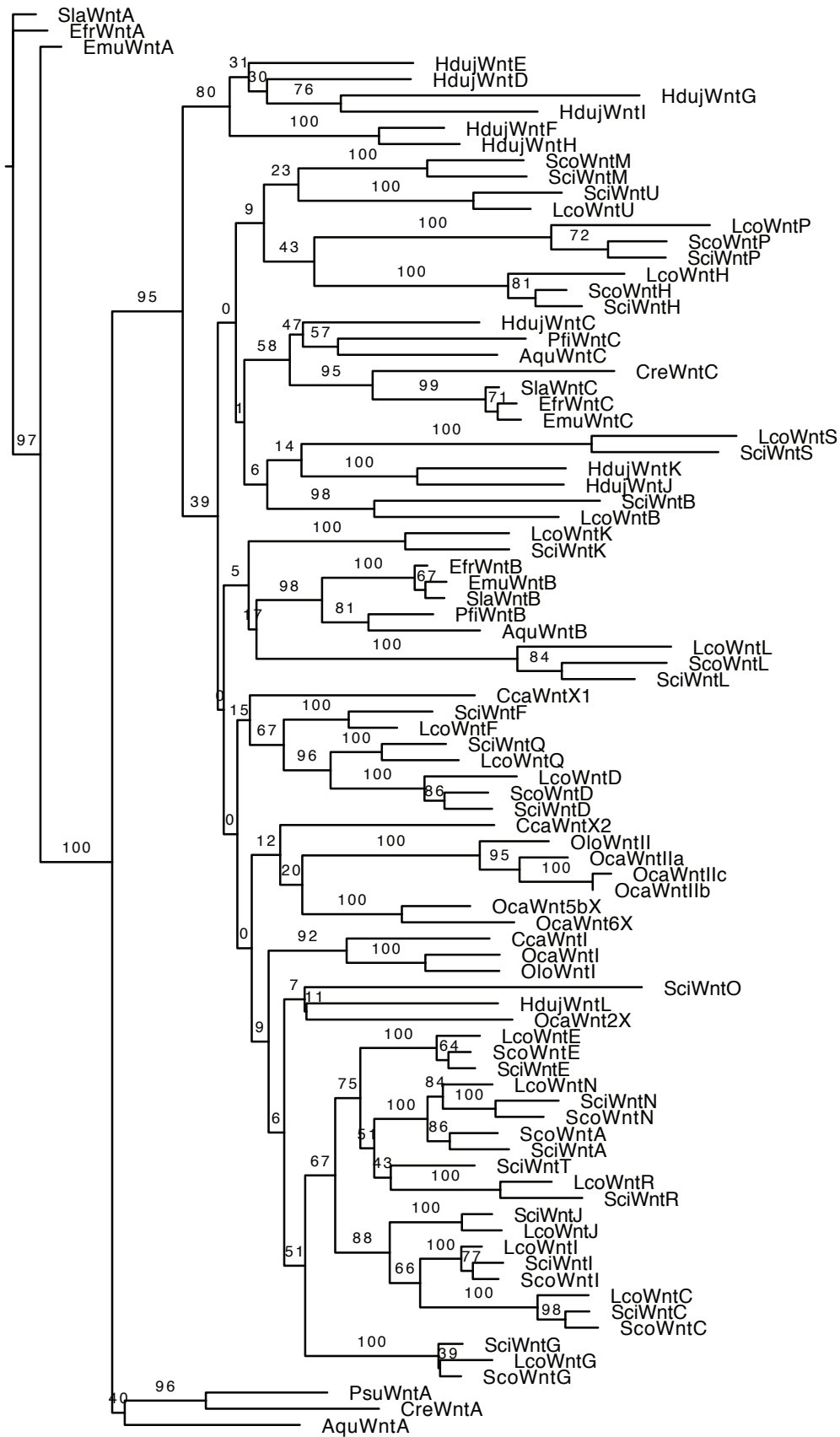

B

## RAxML raw tree

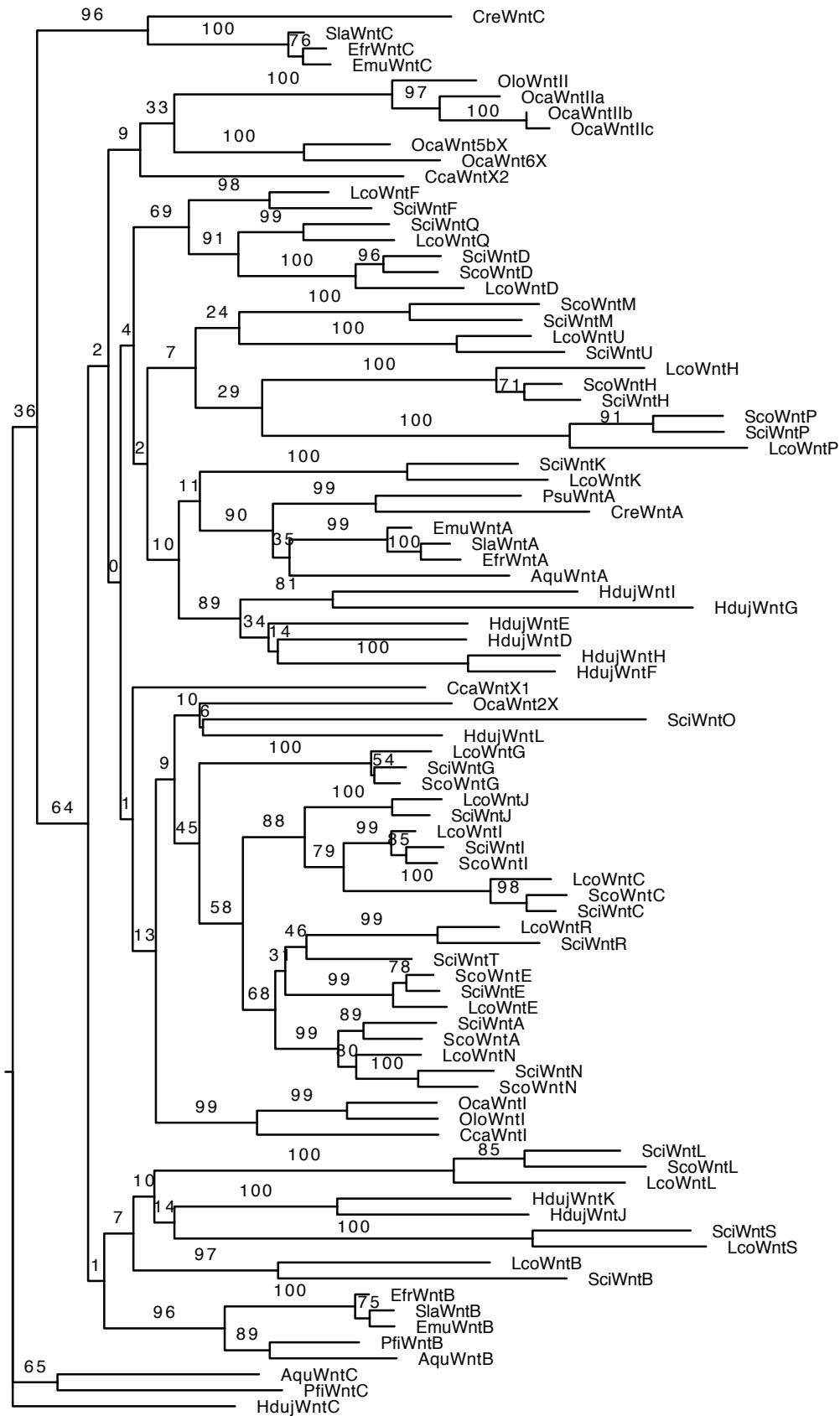

0.3

C

## IQ-TREE raw tree

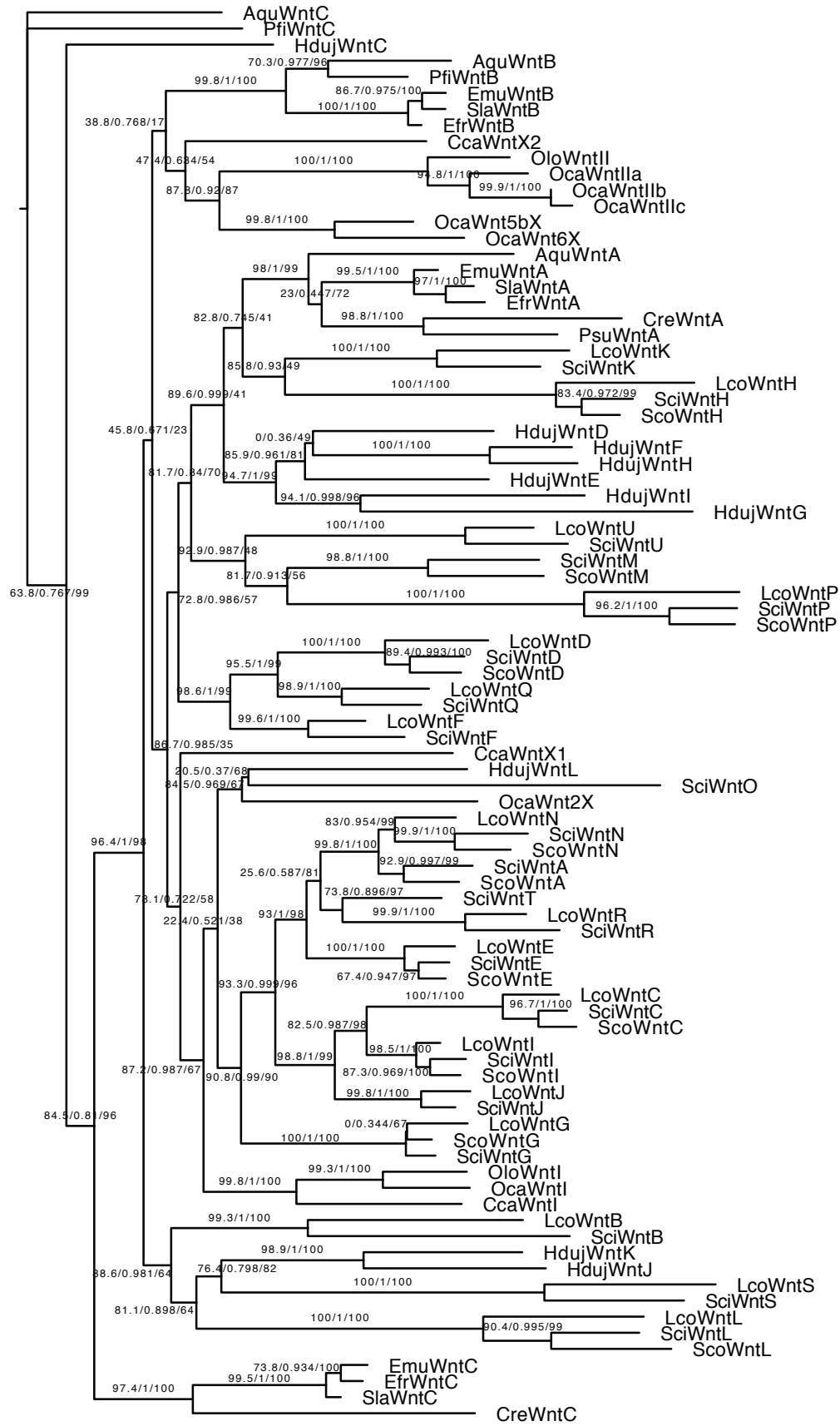

Supplement: Supplementary file 3 — Raw phylogenetic trees used to create the consensus tree presented in Fig. 1. A) PhyML tree with support values from 1000 bootstrap replicates. B) RAxML tree showing bootstrap support from 100 replicates. C) IQ-TREE with support values from 1000 SH-aLRT replicates/aBayes/1000 ultrafast bootstrap replicates. (PDF 820 kb) [file 12862_2018_1118_MOESM3_ESM.pdf]

# Additional File 5

PhyML raw tree  
ALL Wnts

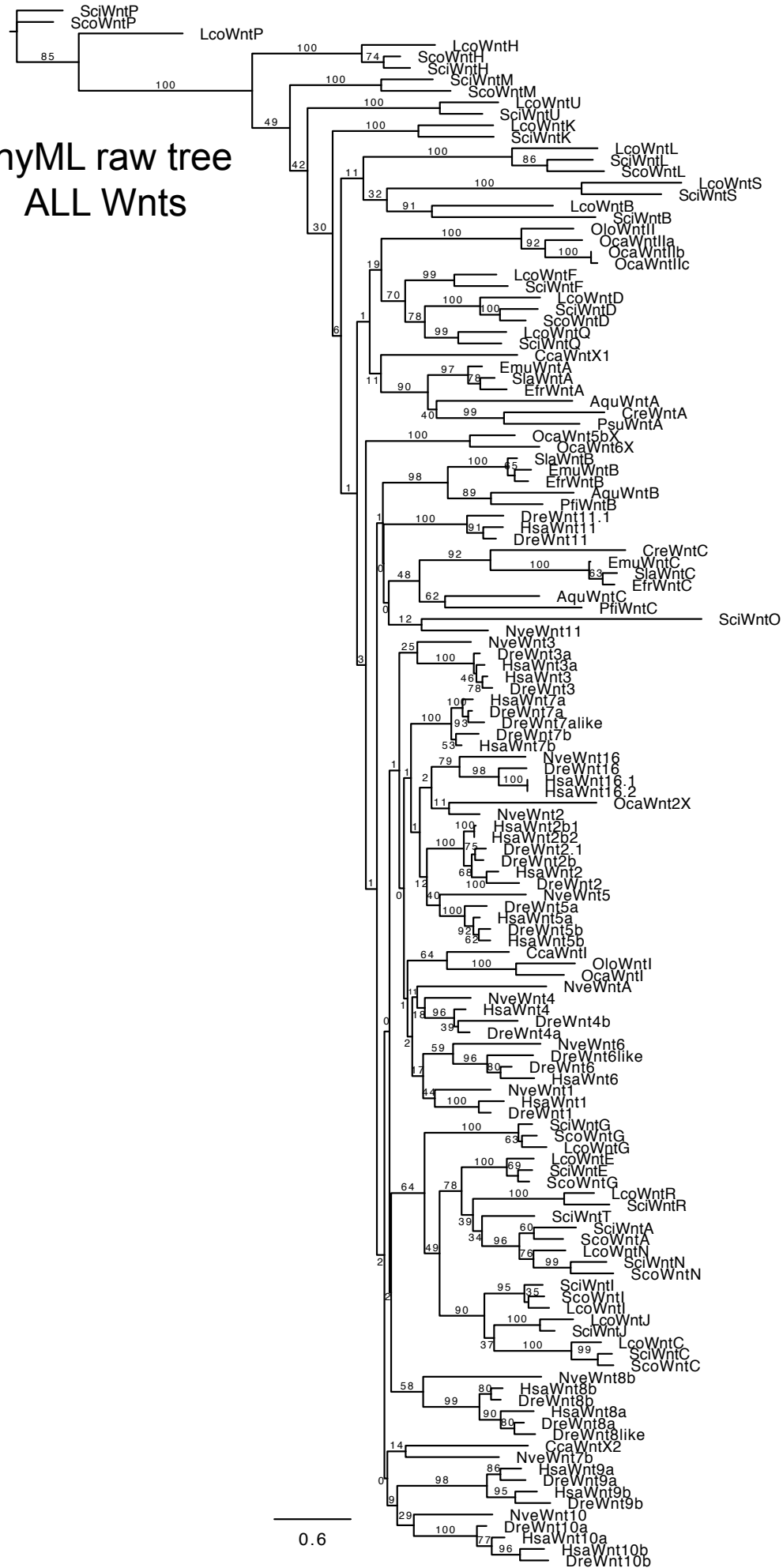

Supplement: Supplementary file 5 — Raw phylogenetic PhyML tree with sponge and bilaterian Wnt sequences. Values displayed are bootstrap support from 100 replicates. Species codes: Nve = Nematostella vectensis, Dre = Danio rerio, Hsa = Homo sapiens, sponge species codes are as listed in the legend of Fig. 1. (PDF 585 kb) [file 12862_2018_1118_MOESM5_ESM.pdf]

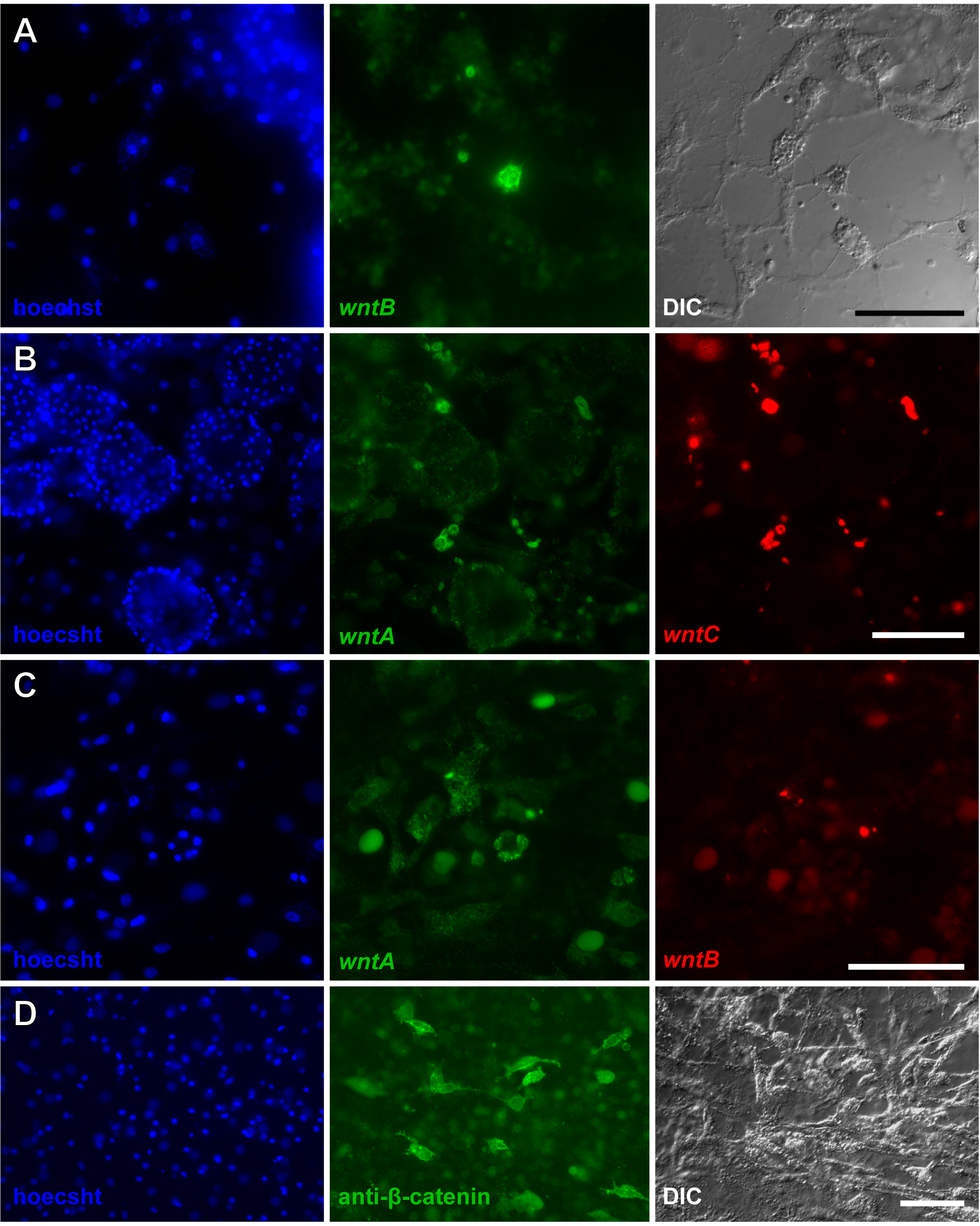

Supplement: Supplementary file 7 — Fluorescent in situ hybridization and antibody images showing separate channels for images shown in Fig. 2. A) wntB, B) wntA/wntC, C) wntA/wntB and D) β-catenin antibody. (TIFF 6439 kb) [file 12862_2018_1118_MOESM7_ESM.tif]

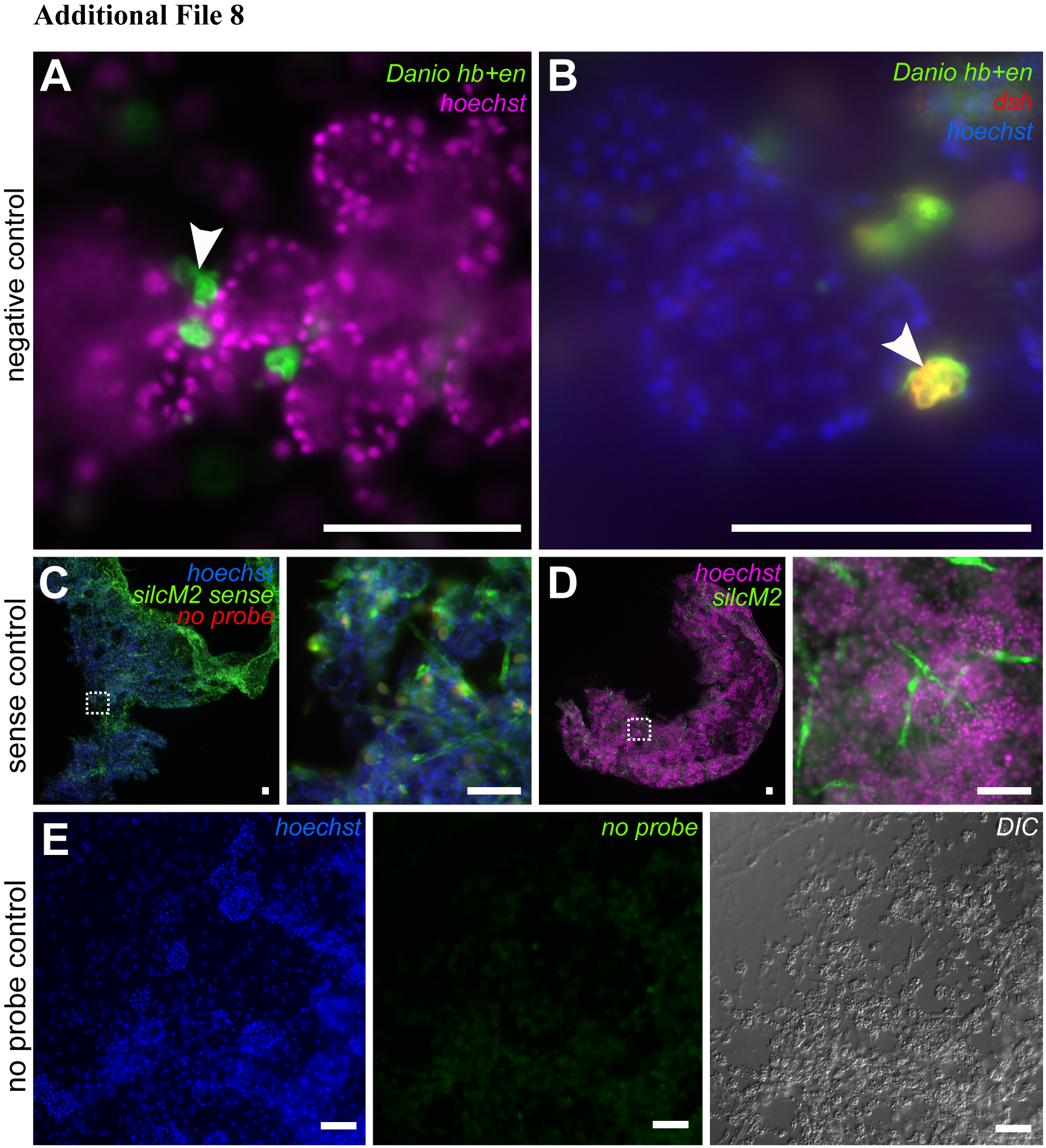

Supplement: Supplementary file 8 — In situ hybridization control experiments. A) A dual probe from Danio rerio against hemoglobin (hb) and engrailed (en) shows a brightly labelled region next to choanocyte chambers (arrowhead). B) Dual probe from D. rerio co-labelled with a dsh probe, showing the same regions labelled next to chaonocyte chambers (arrowhead). Co-staining indicates a lack of full specificity of the sponge dsh probe. C) Low magnification view of a sense probe control for silicatein M2 (silcM2). The boxed area is shown to the right at higher magnification showing diffuse, low-level staining of many cells and structures, including spicules. D) Fluorescent silcM2 label for comparison showing brightly labelled sclerocytes surrounding spicules, and no background staining. Boxed area is shown to the right at a higher magnification. E) Nuclei labelled with hoechst (left), tissue autofluorescence when no probe is applied (middle), and a DIC overview of the region pictured (right). Scales = 50 μm. (TIFF 7044 kb) [file 12862_2018_1118_MOESM8_ESM.tif]

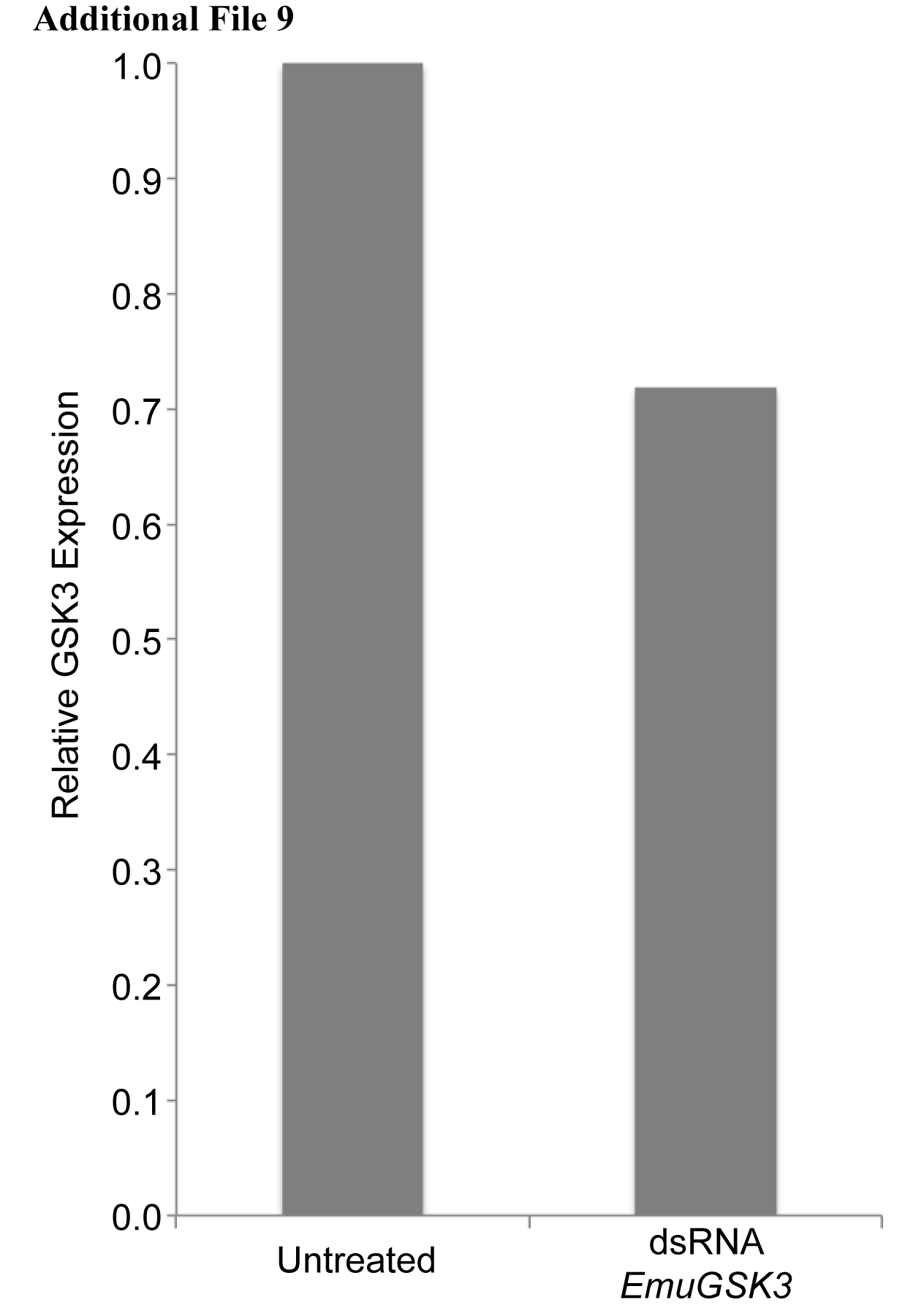

Supplement: Supplementary file 9 — Confirmation of gsk3 knockdown by qPCR. Relative expression levels of gsk3 in untreated versus dsRNA treated sponges (dsRNA EmuGSK3). Expression levels normalized to Ef1-α. (TIFF 319 kb) [file 12862_2018_1118_MOESM9_ESM.tif]

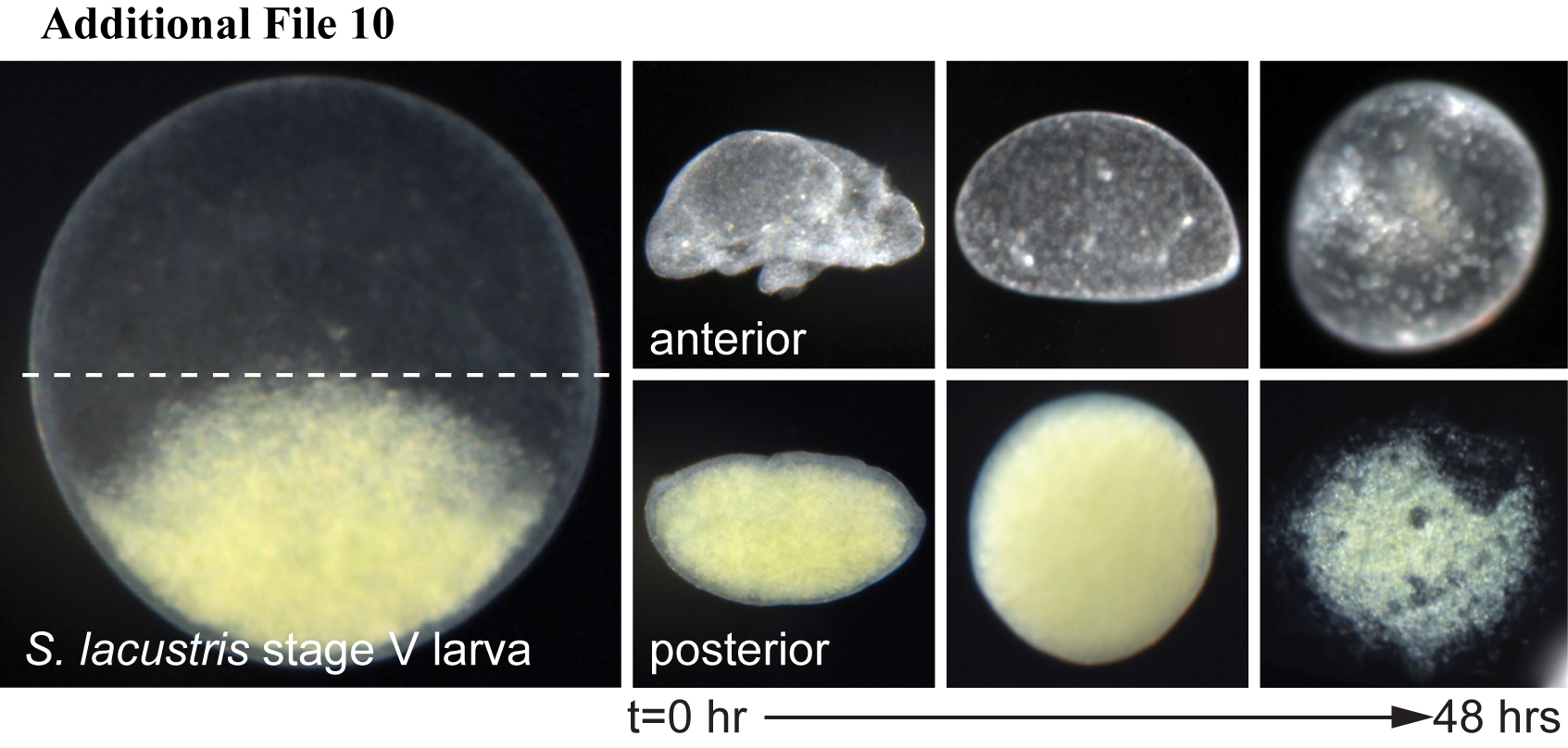

Supplement: Supplementary file 10 — Bisection experiments in Spongilla lacustris larvae have the opposite result of that seen in E. fragilis. Larval appearance is very similar to E. fragilis. However, in S. lacustris the posterior hemisphere settles and forms a normal sponge while the anterior half remains undifferentiated and continues swimming in the water column for up to 3 days. (TIFF 2871 kb) [file 12862_2018_1118_MOESM10_ESM.tif]
