# Supplementary material for: Wnt signaling and polarity in freshwater sponges
Source: BMC Evol Biol. 2018 Feb 2;18:12. doi: 10.1186/s12862-018-1118-0 (PMC5797367; doi:10.1186/s12862-018-1118-0)
Supplement: Supplementary file 4 — Assignment of sponge Wnts to each sponge-specific Wnt subfamily. Groupings were decided by several phylogenetic analyses and are taken from the consensus tree shown in Fig. 1, and subfamily names were retained from [18–20]. (PDF 65 kb) [file 12862_2018_1118_MOESM4_ESM.pdf]

Additional File 4

Wnt Subfamily	Sequence Name	Accession/Contig Number
**PorWntA**	AquWntA	ABX90060
	CreWntA	Contig66998
	PsuWntA	Contig252
	EmuWntA	Contig16045*
	SlaWntA	Contig4731
	EfrWntA	Contig21562
**PorWntB**	AquWntB	ADO16564
	PfiWntB	Contig51648
	EmuWntB	ADM13617
	SlaWntB	Contig12305*
	EfrWntB	Contig28264
**PorWntC**	AquWntC	ADO16565
	PfiWntC	Contig10714
	CreWntC	Contig73781
	HdujWntC	HADA01000006
	EmuWntC	Contig23736*
	SlaWntC	Contig31918
	EfrWntC	Contig14524
**PorWntI**	OcaWntI	m.2356
	OloWntI	ACS36174
	CcaWntI	Contig 9259
**PorWntII**	OcaWntIIa	m.10180
	OcaWntIIb	m.18610
	OcaWntIIc	m.18615
	OloWntII	ACS36175
**CWntA/N**	SciWntA	CDO67888
	ScoWntA	Contig22889*
	SciWntN	CDO67901
	ScoWntN	Contig17541
	LcoWntN	lcpid60387|lcgid45017
**CWntB**	SciWntB	CDO67889
	LcoWntB	lcpid67615|lcgid28365
**CWntC**	SciWntC	CDO67890
	ScoWntC	Contig16518
	LcoWntC	lcpid75928|lcgid35509
**CWntD**	SciWntD	CDO67891
	ScoWntD	Contig5782
	LcoWntD	lcpid87580|lcgid0311
**CWntE**	SciWntE	CDO67892
	ScoWntE	Contig14145
	LcoWntE	lcpid93240|lcgid0654
**CWntF**	SciWntF	CDO67893
	LcoWntF	lcpid61054|lcgid2141

**CWntG**	SciWntG	CDO67894
	ScoWntG	Contig38625
	LcoWntG	lcpid12644|lcgid42850
**CWntH**	SciWntH	CDO67895
	ScoWntH	Contig29445
	LcoWntH	lcpid72229|lcgid34130
**CWntI**	SciWntI	CDO67896
	ScoWntI	Contig12941
	LcoWntI	lcpid41001|lcgid58842
**CWntJ**	SciWntJ	CDO67897
	LcoWntJ	lcpid19806|lcgid65167
**CWntK**	SciWntK	CDO67898
	LcoWntK	lcpid80837|lcgid23822
**CWntL**	SciWntL	CDO67899
	ScoWntL	Contig18533
	LcoWntL	lcpid58524|lcgid12336
**CWntM**	SciWntM	CDO67900
	ScoWntM	Contig57224
**CWntO**	SciWntO	CDO67902
**CWntP**	SciWntP	CDO67903
	ScoWntP	Contig26375
	LcoWntP	lcpid80785|lcgid6025
**CWntQ**	SciWntQ	CDO67904
	LcoWntQ	lcpid74153|lcgid17617
**CWntR**	SciWntR	CDO67905
	LcoWntR	lcpid66521|lcgid3137
**CWntS**	SciWntS	CDO67906
	LcoWntS	lcpid43958|lcgid43546
**CWntT**	SciWntT	CDO67907
**CWntU**	SciWntU	CDO67908
	LcoWntU	lcpid104953|lcgid65306
**Unassigned**	HdujWntD	HADA01000005
	HdujWntE	HADA01000010
	HdujWntF	HADA01000003
	HdujWntG	HADA01000002
	HdujWntH	HADA01000004
	HdujWntI	HADA01000001
	HdujWntJ	HADA01000009
	HdujWntK	HADA01000007
	HdujWntL	HADA01000008
	OcaWnt2X	m.82490
	OcaWnt5bX	m.13224
	OcaWnt6X	m.25362
	CcaWntX1	Contig 400
	CcaWntX2	Contig 9097
