# Supplementary material for: Wnt signaling and polarity in freshwater sponges
Source: BMC Evol Biol. 2018 Feb 2;18:12. doi: 10.1186/s12862-018-1118-0 (PMC5797367; doi:10.1186/s12862-018-1118-0)

Additional File 6

SUPPLEMENTAL METHODS & RESULTS

To test the validity of our antibody against *Spongilla lacustris* β-catenin, we ran our injected antigen (expected size = 20.2 kDa, small black arrowhead) alongside whole sponge lysate on a polyacrylamide gel. We transferred proteins from the gel onto PVDF membrane for 30 minutes at 350mA in transfer buffer (50% methanol, 30%. The membrane was cut to into strips containing each sample (injected antigen and sponge lysate) and blocked in 3% ovalbumin in TBST (Tris-buffered saline + 0.1% Triton X-100) for 1 hour at room temperature.

Each serum sample ("Bleed 4", "Bleed 5", and "Final antibody") was pre-absorbed overnight at 4°C to a distinct *Spongilla lacustris* peptide, a fragment of dishevelled protein derived from the same method as β-catenin peptide (see main text, Methods). Membranes were incubated at 4°C overnight while rocking, in pre-absorbed anti-Slaβcat (1:100 in TBST).

Membranes were subsequently washed in TBST, then incubated in secondary antibody; we used Alexa 488 goat anti-rabbit at 1:1000 in 3% BSA in TBST for 1 hour at room temperature. Membranes were washed again in TBS, and finally imaged on a Fuji FLA-5000 Imager.

The estimated size of endogenous β-catenin is 96 kDa based on sequence alone as estimated on the ProtParam feature of ExPASy (http://www.expasy.org). However, Schippers and Nichols (2017; [23]) found that their custom antibody labeled endogenous *Ephydatia muelleri* β-catenin at roughly 110 kDa, which is similar in size to the band detected by our antibody indicated in the image below (white arrowhead).

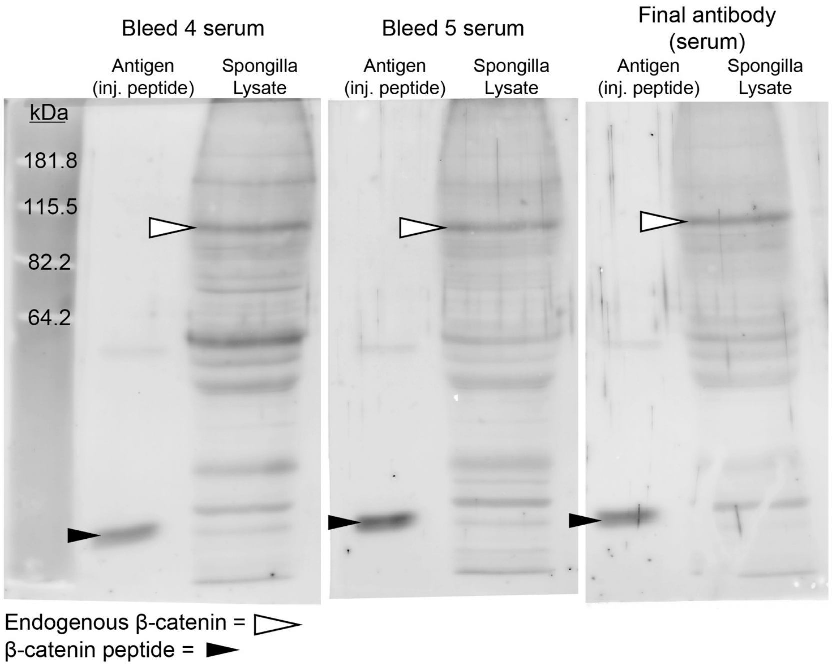

Supplement: Supplementary file 6 — Western blot validation of our custom antibody to a β-catenin fusion protein from Spongilla lacustris. (PDF 349 kb) [file 12862_2018_1118_MOESM6_ESM.pdf]
