# Supplementary material for: Wnt signaling and polarity in freshwater sponges
Source: BMC Evol Biol. 2018 Feb 2;18:12. doi: 10.1186/s12862-018-1118-0 (PMC5797367; doi:10.1186/s12862-018-1118-0)
Supplement: Supplementary file 11 — Accession numbers of previously published sequences used for phylogenetic analysis in Fig.1 and Additional files 3 and 5. (PDF 64 kb) [file 12862_2018_1118_MOESM11_ESM.pdf]

Additional File 11

Species	Sequence Name	NCBI Accession
Amphimedon queenslandica	AquWntA	ABX90060
	AquWntB	ADO16064
	AquWntC	ADO16565
Ephydatia muelleri	EmuWntB	ADM13617
Oscarella lobularis	OloWntI	ACS36174
	OloWntII	ACS36175
Nematostella vectensis	NveWntA	AAT02182
	NveWnt1	AAT00640
	NveWnt2	AAW28132
	NveWnt3	ABF48092
	NveWnt4	AAV87174
	NveWnt5	AAW28133
	NveWnt6	AAW28134
	NveWnt7b	AAW28135
	NveWnt8b	AAW28136
	NveWnt10	AAT00641
	NveWnt11	AAV87175
	NveWnt16	ABF48091
Danio rerio	DreWnt1	NP_001188327
	DreWnt2	NP_571025
	DreWnt2b	NP_001037809
	DreWnt2.1	NP_878296
	DreWnt3	NP_001108024
	DreWnt3a	NP_001007186
	DreWnt4a	NP_001035477
	DreWnt4b	NP_571575
	DreWnt5a	NP_001073303
	DreWnt5b	NP_571012
	DreWnt6	XP_003199237
	DreWnt6like	XP_002662357
	DreWnt7a	NP_001020711
	DreWnt7alike	XP_696514
	DreWnt7b	XP_691878
	DreWnt8a	NP_571021
	DreWnt8b	NP_571034
	DreWnt8like	NP_00108637
	DreWnt9a	NP_001038828
	DreWnt9b	NP_001131132
	DreWnt10a	NP_571055
	DreWnt10b	NP_835737
	DreWnt11	NP_571151
	DreWnt11.1	NP_001138276
	DreWnt16	NP_001093516
Homo sapiens	HsaWnt1	NP_005421
	HsaWnt2	NP_003382
	HsaWnt2b1	NP_004176
	HsaWnt2b2	NP_078613

	HsaWnt3	NP_110380
	HsaWnt3a	NP_149122
	HsaWnt4	NP_110388
	HsaWnt5a	NP_003383
	HsaWnt5b	NP_110402
	HsaWnt6	NP_006513
	HsaWnt7a	NP_004616
	HsaWnt7b	NP_478679
	HsaWnt8a	NP_490645
	HsaWnt8b	NP_003384
	HsaWnt9a	NP_003386
	HsaWnt9b	NP_003387
	HsaWnt10a	NP_079492
	HsaWnt10b	NP_003385
	HsaWnt11	NP_004617
	HsaWnt16.1	NP_476509
	HsaWnt16.2	NP_057171
