# Supplementary material for: Wnt signaling and polarity in freshwater sponges
Source: BMC Evol Biol. 2018 Feb 2;18:12. doi: 10.1186/s12862-018-1118-0 (PMC5797367; doi:10.1186/s12862-018-1118-0)
Supplement: Supplementary file 12 — Primers used to amplify sequences for RNAi and mRNA injection experiments. (PDF 66 kb) [file 12862_2018_1118_MOESM12_ESM.pdf]

Additional File 12

Purpose	Primer Name	Sequence (5′ to 3′)
**Riboprobe Synthesis**	pEmuSilcM2 F	TGG CAA GTT TAG CAA TGG CTG TCC
	pEmuSilcM2 R	CAC CAG CAT GGC ATG ATT GAG CTT
	pEmuWntA F	GGT AAG AAA CTC AGG ATG CC
	pEmuWntA R	CCC AAA GTA AAC GTA GCG TC
	pEmuWntB F	GTG CAT CTG GGT ATT TCA CG
	pEmuWntB R	CCG ACA TAG CTA GTC ATG TTG GAG
	pEmuWntC F	GAT CAA TAT GCT GGA GTG CC
	pEmuWntC R	CCG TAG ATC AGA GTT CTA TCG GTG
	pEmuBcat F	GAG TCA AGA CGA GAT AGT GGA G
	pEmuBcat R	GAT AGG ATG GAG TAT GGG ACT G
	pEmuDsh F	CAA CAG ACC TAG ATG CTA CC
	pEmuDsh R	GGA AGA GGT CCA AGT GTA TC
	pEmuGSK3 F	GTA CAG AGT GAT ACG ACA CC
	pEmuGSK3 R	CCG CCT AAA CTC AAC AGA AG
	pEmuFz1 F	GTC TCC TCT TCA CCT TCT TCA C
	pEmuFz1 R	GAG TAG AGA AAC TAG ACT GCC G
	pEmuFz2/4 F	CGA CTA ACT CTA CAT GTG TCG G
	pEmuFz2/4 R	GAA CTG TCA TGA CCA GAG GTA G
	pEmuFz3 F	GCT AGA GGG CTA TTC TAC CTT TCC
	pEmuFz3 R	CTT AAG TCT GAG GAG GAG GAT G
	pEmuTCF F	GAC GAG TAA TGT CAG CAC AG
	pEmuTCF R	CTT CTT CTT ACG CCT ACA CG
**RNAi**	RNAi-EmuGSK3 F	*CGA CTC ACT ATA GGG* CCG TCA CGT AAC AGG ACT AC*
	RNAi-EmuGSK3 R	*CGA CTC ACT ATA GGG* AGA CAA CCC CAA ATG ATC CA
	RNAi-SilcM2 F	*CGA CTC ACT ATA GGG* GGA GAG ACA TGC CAT TTG GT
	RNAi-SilcM2 R	*CGA CTC ACT ATA GGG* CTG GAC ACC TTT CGG AGC TA
	Full T7 F-R	*ATA GAA TTC TCT AGA AGC TTA ATA CGA CTC ACT ATA GGG*

* Text in italics indicates a T7 universal sequence, regular text is sequence-specific.
